# Identification of Graphene Dispersion Agents through Molecular Fingerprints

**DOI:** 10.1021/acsnano.2c04406

**Published:** 2022-09-27

**Authors:** Stuart J. Goldie, Matteo T. Degiacomi, Shan Jiang, Stewart J. Clark, Valentina Erastova, Karl S. Coleman

**Affiliations:** † Department of Chemistry, Durham University, South Road, Durham, DH1 3LE, United Kingdom; ‡ Department of Physics, Durham University, South Road, Durham, DH1 3LE, United Kingdom; § School of Chemistry, University of Edinburgh, David Brewster Road, Edinburgh, EH9 3FJ, United Kingdom

**Keywords:** graphene, 2D materials, exfoliation, molecular modeling, solvent prediction

## Abstract

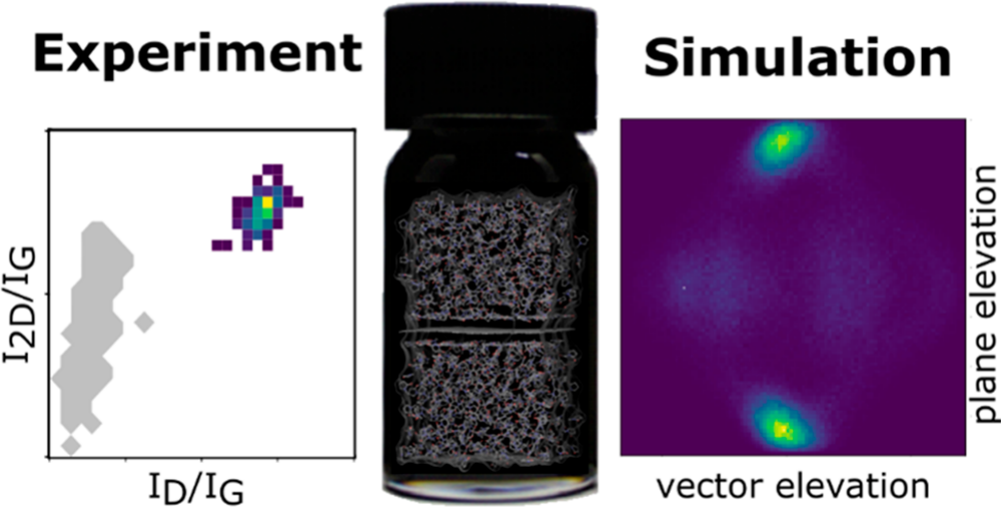

The scalable production and dispersion of 2D materials, like graphene, is critical to enable their use in commercial applications. While liquid exfoliation is commonly used, solvents such as *N*-methyl-pyrrolidone (NMP) are toxic and difficult to scale up. However, the search for alternative solvents is hindered by the intimidating size of the chemical space. Here, we present a computational pipeline informing the identification of effective exfoliation agents. Classical molecular dynamics simulations provide statistical sampling of interactions, enabling the identification of key molecular descriptors for a successful solvent. The statistically representative configurations from these simulations, studied with quantum mechanical calculations, allow us to gain insights onto the chemophysical interactions at the surface–solvent interface. As an exemplar, through this pipeline we identify a potential graphene exfoliation agent 2-pyrrolidone and experimentally demonstrate it to be as effective as NMP. Our workflow can be generalized to any 2D material and solvent system, enabling the screening of a wide range of compounds and solvents to identify safer and cheaper means of producing dispersions.

## 1 Introduction

Graphene has unique chemical and physical properties, such as record tensile strength and flexibility,^1^ gas impermeability,^2^ near-transparency to visible light,^3^ and high thermal and electrical conductivity.^4,5^ To capitalize on these exciting properties for functional materials, it is essential that simple, commercially viable, ecological, and scalable manufacturing techniques of single-layer graphene are in place. Such techniques should also be applicable to other 2D nanomaterials with an even greater range of properties and thereby devices.

The demonstration of solvent exfoliation in 2008 allowed the manufacture of graphene in kilogram batches.^6^ Among solvent exfoliation techniques, high shear mixing and microfluidization are the most commonly used for large-scale graphene production.^7−9^ Exfoliation is a result of mechanically driven tearing and peeling of incrementally smaller flakes away from a graphite crystal; the solvent then stabilizes the freshly formed graphene flakes, preventing aggregation.^7,10,11^ However, graphene produced through solvent exfoliation is often of low quality, featuring inhomogeneity and imperfections.^12,13^ Unfortunately, the best solvents capable of minimizing the damage to graphene during production are also toxic. In particular, *N*-methyl-pyrrolidone (NMP), has been labeled as a substance of very high concern by the European Chemicals Agency with restrictions on use, making it undesirable for larger-scale production (Table S1.3). The aim for scalable manufacture is, therefore, a solvent capable of producing graphene dispersions of high quality while being safe to handle and exploit on a large scale.

In the search for alternative solvents, different metrics and descriptors have been used to predict graphene stabilization performance. Rationalizing that a stable dispersion requires the graphene–solvent interface to be as close in energy as possible to the favorable solvent–solvent interactions, Hernandez et al. proposed that the surface tension of a solvent should match that of graphite.^8^ To account for the different interactions within the system (dispersive, polar, and hydrogen bonding), they derived Hansen solubility parameters for graphene from the values of known effective solvents.^14^ When discussing Hansen parameters for predictions, it is important to consider the large data set that must be collected for any new 2D materials to calculate the material’s parameters before any predictions can be made (see Supporting Information Section 1.2 for the full discussion). These metrics have been used successfully to rationalize the performance of many common laboratory solvents,^15^ usually by exclusion of those with significantly lower (e.g., acetone) or higher (e.g., water) surface tensions than graphene. Nevertheless, many other solvents have comparable surface tensions yet are ineffective exfoliation agents.

Surface tension arises due to an imbalance of cohesive forces at the liquid–vapor interface (i.e., it is of homogeneous nature). The interfacial tension, in contrast, describes heterogeneous interactions, such as between a solvent and a surface. Here, adhesive forces give rise to molecular structuring at the interface, thin-film formation, and wetting phenomena. Therefore, surface tension alone is insufficient to describe the heterogeneous physicochemical interactions, key to understanding the processes at the interface such as aggregation of graphene flakes in a solvent.

To this end, we use molecular simulations to gain atomic-level details on the structures, dynamics, and interactions at the interface. We develop a computational pipeline leveraging molecular dynamics simulations for characterization of the interactions between solvent molecules and 2D materials. Representative molecular states are then automatically selected and interrogated at higher resolution via density functional theory calculations. Applying this pipeline to the case of graphene, we identify a correlation between the surface arrangement of common solvent molecules and their effectiveness at stabilizing graphene dispersions. Using this methodology, and as an exemplar, we were able to predict and experimentally validate, 2-pyrrolidone, a solvent with equal liquid exfoliation performance to NMP, the best performing solvent currently in wide use (see Supporting Information Section 1.3 for a comparison of solvents used in previous studies).

## 2 Results

### 2.1 Modeling Pipeline

**Figure 1:**
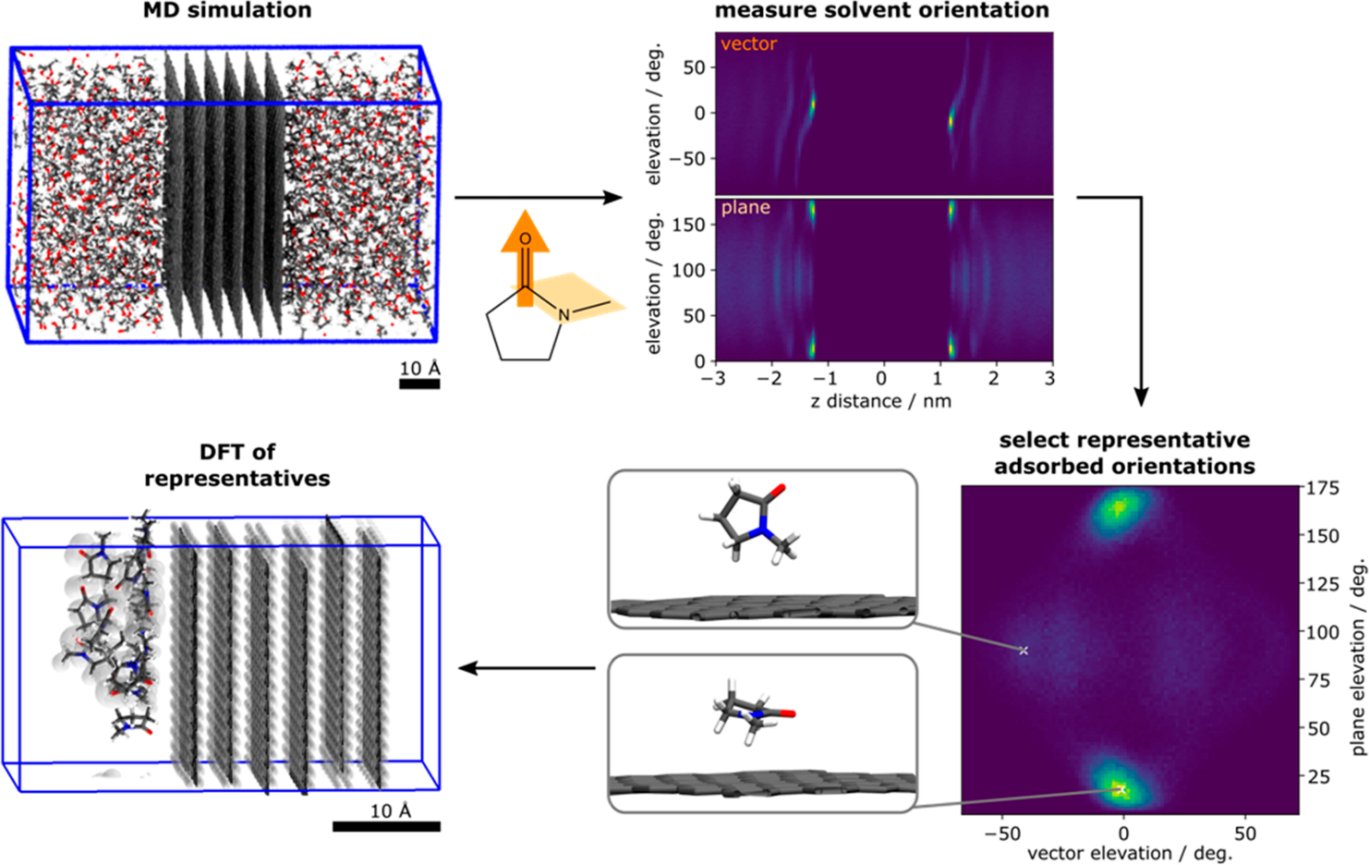
Modeling pipeline. A graphite surface is surrounded with a solvent of interest, and simulated with MD (top left, graphite layers are shown in black). The orientation of every solvent molecule is described by a vector (orange arrow) and a plane (yellow rhomboid); see details in Figure S3.1. The orientation of each solvent as a function of distance from the surface is then extracted (shown as a 2D probability density, top right). From the orientational distribution of species in the first solvation shell only (shown as a 2D probability density, bottom right), representative arrangements are identified (see examples in the two insets). The coordinates of the representative solvent, surface underneath and a shell of solvent, are extracted and subjected to planewave DFT calculations (bottom left).

Our modeling pipeline is graphically represented in Figure 1; for full details see the “Methods” section. We begin with molecular dynamics (MD) simulations of the 2D or layered material, in this case graphene or graphite, surrounded by each of the chosen solvents. To describe the interactions of the solvent and the surface, we assign each solvent molecule geometric labels, enabling us to define the orientation of each of the hundreds of molecules with respect of the surface of the material. The molecular orientations are then considered as statistical populations, hereafter called *fingerprints.* We cluster these populations into subsets, allowing us to automatically identify representative configurations of solvent molecules in contact with the surface. The coordinates of each identified representative configuration, which includes a central solvent molecule, the surface beneath and a shell of surrounding solvent molecules (see section 2.4 for details on the choice of solvent shell size), are extracted for planewave density functional theory (DFT) calculations to investigate any solvent-mediated changes in the electronic structure.

### 2.2 Choice of Molecular Systems

In order to identify and quantify the specific solvent-surface interactions that stabilize exfoliated graphene layers and prevent aggregation, we use MD simulations to screen through a number of solvents with known performance for graphene exfoliation. Our strategy is to compare and contrast interactions of a set of solvents known to exhibit different exfoliation capabilities. First, we selected *N*-methyl-pyrrolidone (NMP), a commonly used solvent, very effective at exfoliation.^16^ We then included solvents with poor exfoliation capabilities, specifically water, ethanol, and acetone.^8^ Finally, we selected two solvents that, having similar physical properties to NMP, are used as substitutes in a laboratory setting: *N*,*N*-dimethylformamide (DMF) and dimethyl sulfoxide (DMSO). DMF is already known to be an exfoliating solvent, albeit generally producing a lower graphene concentration.^17^ Conversely, DMSO is ineffective for exfoliation,^18,19^ despite having properties such as surface tension and Hansen solubility parameters remarkably similar to those of NMP. All the solvents used in this study and their physical properties, solubility parameters, and toxicity are summarized in Tables S1.1–S1.3, respectively.

### 2.3 Quantifying Solvent–Surface Interactions through Molecular Dynamics

**Figure 2:**
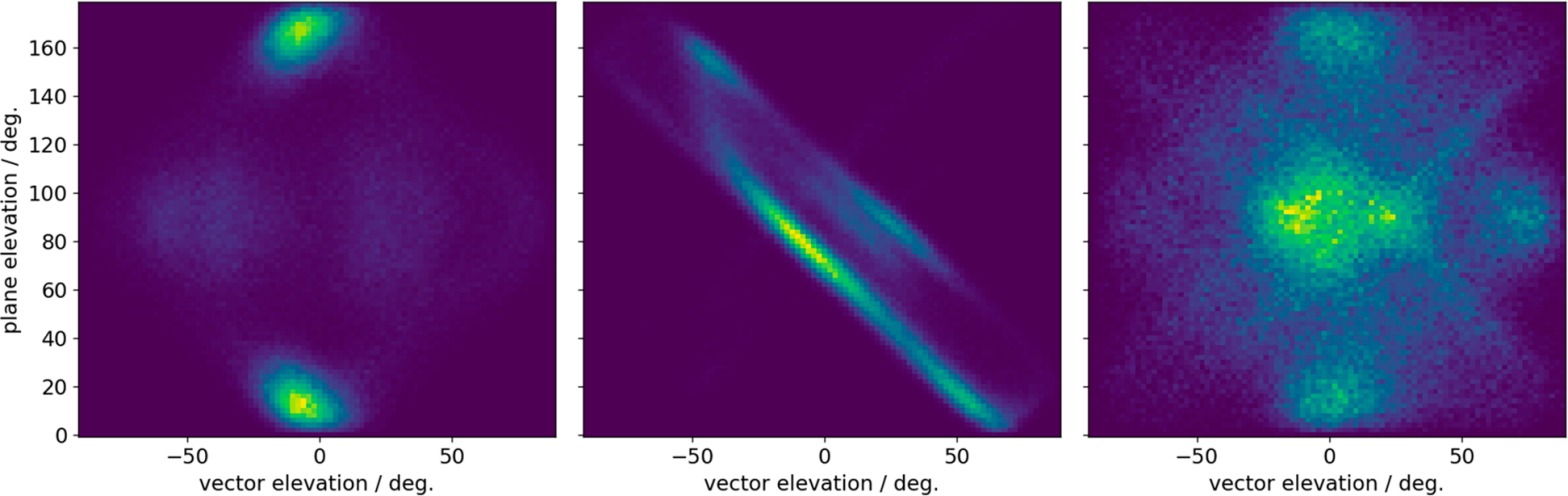
Fingerprints of the solvent orientation on a graphene sheet. The plot is a statistical distribution of the surface-coating solvent molecule orientations, showing the most probable orientations in lighter colors, as described by the elevation of the vector (*x*-axis) and of the plane (*y*-axis), see details in Figure S3.1. (a) NMP features a strong symmetric alignment preference. (b) DMSO features strong lines describing a range of preferred alignments. (c) Ethanol does not feature any prominent surface ordering.

We carried out MD simulations of each selected solvent with both graphene and graphite. In our pipeline, the orientation of each solvent molecule is described by a plane and a vector in spherical coordinates (Table S3.1). This approach is a form of dimensionality reduction, allowing us to comprehensively describe the orientation of the solvent with respect to the surface using only four values: elevation and azimuth of the vector and the plane, without strict dependency on the chosen referential.^20,21^ Since graphene surfaces feature a 6-fold symmetry, azimuth values hold little additional information (Figure S3.2), allowing us to further reduce our descriptors into a two-dimensional plot of vector vs plane elevations for surface coating solvent molecules (Figure S3.3). From these statistical distributions, we observe that the known effective solvents share a similar pattern, (Figure 2a). The other solvents either feature completely different fingerprints (Figure 2b) or random distributions (Figure 2c).

**Figure 3:**
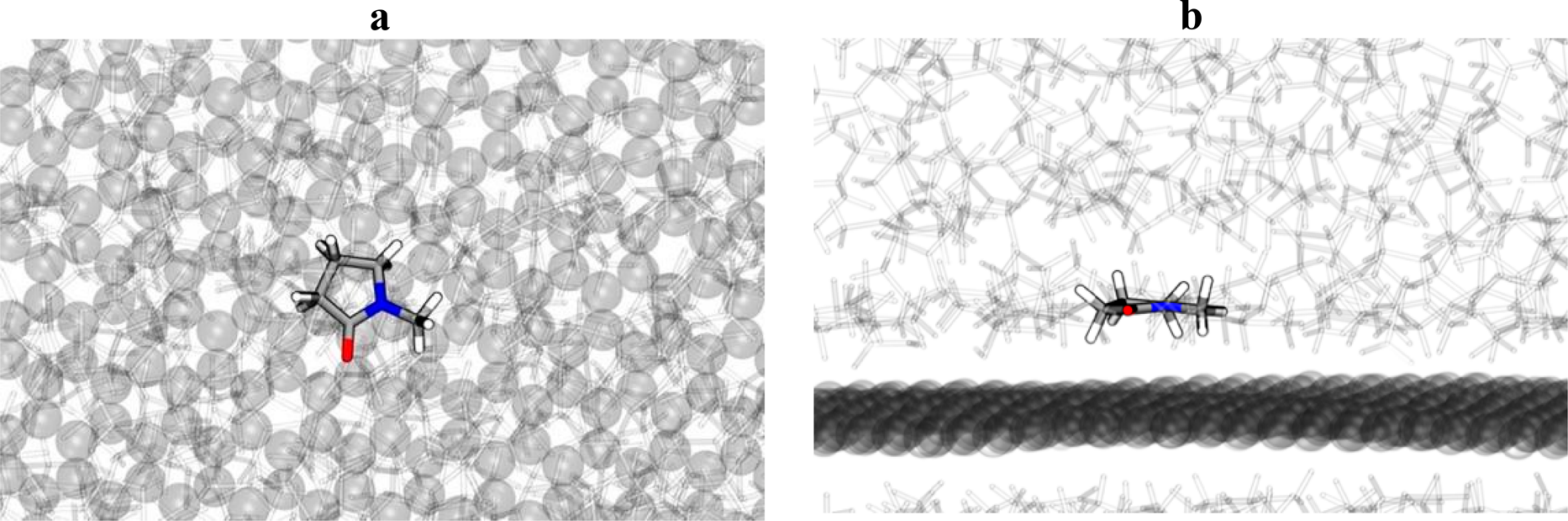
NMP solvent over the surface of graphene sheet, (a) showing the top view, and (b) a side view. The rendering highlights a single NMP molecule, representative of most common orientation, interacting with graphene (transparent spheres) and surrounded by other NMP solvent molecules (transparent). Colors are as follows: gray for carbon, red for oxygen, blue for nitrogen, and white for hydrogen atoms.

The effective solvents, NMP and DMF, shared a well-defined fingerprint corresponding to an alignment of the delocalized system near-parallel to the graphene sheet (Figure 3). We hypothesized this highlighted interaction to be key for exfoliated graphene stabilization by a solvent. To test this hypothesis, we selected three solvents with similar delocalized systems and unknown graphene exfoliation performance. Two of them, 2-pyrrolidone (PRL) and 1,3-dimethyl-2-imidazolidinone (DMI), feature the same cyclic amide structural motif as NMP, while the third, cyclopentanone (CPN), has a five-membered polarized cyclic structure.

**Figure 4:**
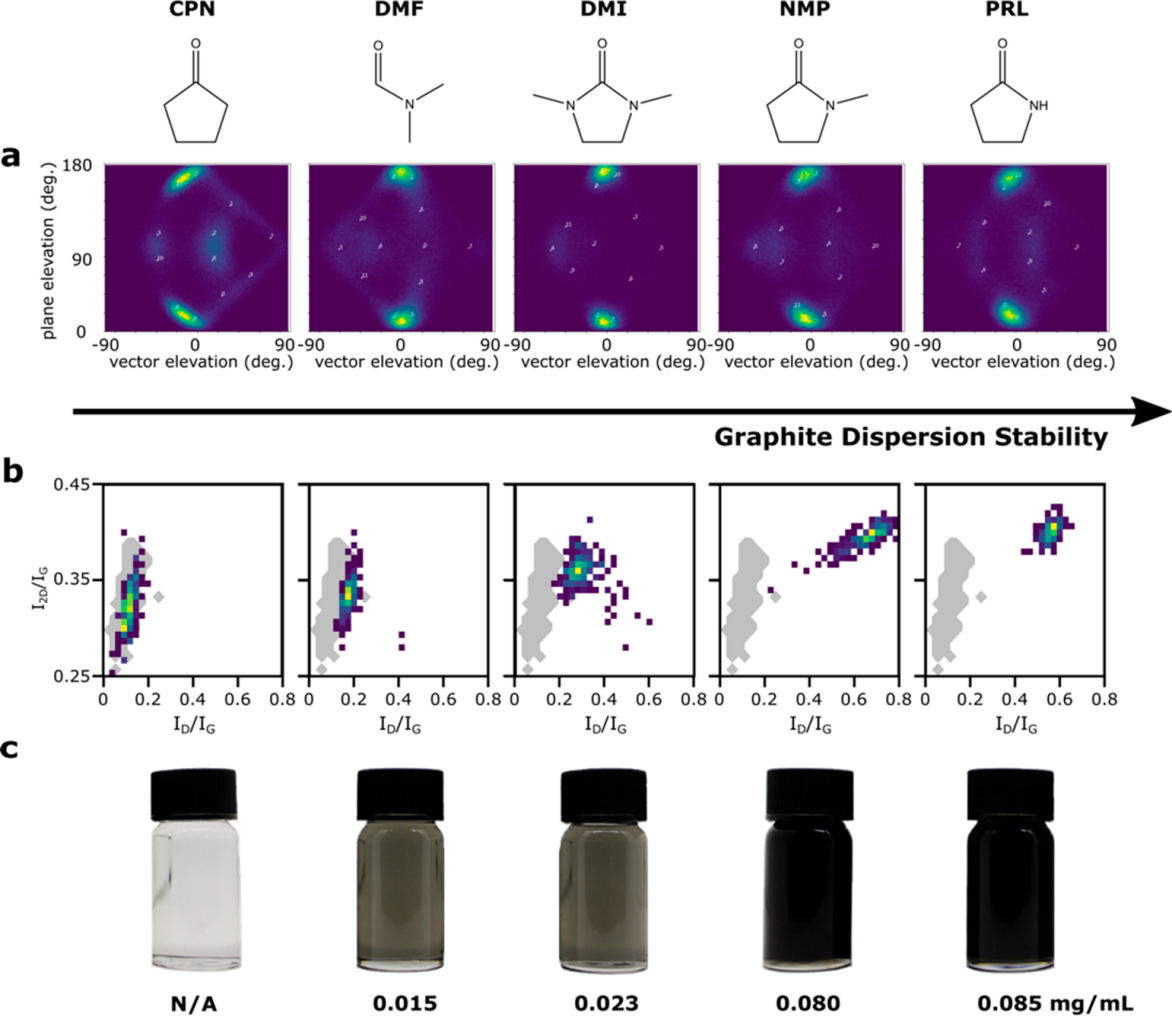
Experimental evaluation of solvent exfoliating effectiveness. For each of the experimentally tested solvents: cyclopentanone (CPN), dimethylformamide (DMF), 1,3-dimethyl-2-imidazolidinone (DMI), *N*-methyl-pyrrolidone (NMP), 2-pyrrolidone (PRL). (a) Fingerprints describing solvent orientation on a graphene surface (see Figure 2 and the Supporting Information for details). (b) Statistical Raman analysis presented as 3D heat maps, where yellow denotes a higher sample population, and gray represents the region occupied by a graphite sample. (c) Photos of the vials with solvents after shear mixing and centrifugation; the concentrations of graphene flakes in the solvent are given under images.

After simulating graphite and graphene immersed in these solvents, results summarized in Figure 4a, our computational analysis shows the fingerprint of PRL featured a well-defined signal like that of NMP, highlighting it as a promising solvent to stabilize graphene exfoliation. DMI featured a slightly different fingerprint, appearing flatter to the surface not dissimilar to the DMF alignment. Finally, CPN shows an even greater difference in alignment to the NMP with more disorder shown by the greater spread of bright areas in the 2D plot.

Thus, from our computational analysis, we do not expect CPN to be a good solvent for supporting exfoliated graphene dispersion, DMI is predicted to perform moderately well, akin to DMF, and PRL is anticipated to be a good solvent for exfoliation. These predictions are in contrast to those expected from Hansen solubility parameters, this is discussed in more detail in Supporting Information Section 1.2.

### 2.4 Experimental Assessment of Solvent Performance

To test our computational predictions on the effectiveness of the hypothesized solvents DMI, CPN, and PRL, we compared their graphene dispersion performance to that of the commonly used solvents NMP and DMF in a laboratory. We shear mixed natural flake graphite in each of these five solvents, removed the undispersed graphite by centrifugation, and analyzed the supernatant (Figure 4c). UV–vis–nIR spectroscopy was used to estimate material concentration; statistical Raman was used to measure degree of exfoliation. Microscopy images from atomic force microscopy (AFM), scanning electron microscopy (SEM), and transmission electron microscopy (TEM) were used to measure lateral size and thickness of the flakes (see the “Methods” section for details).

The statistical Raman analysis followed the workflow introduced previously. In brief, by collecting many spectra (example spectra in Figure S5.1) from a material and plotting a 3D bivariant histogram, statistically significant changes to the material can be observed.^22^ A 3D histogram shows the occupancy of bins as a heat map, while the bins are simultaneously described by two metrics, in this case the intensity ratios of Raman peaks: *I*_D_/*I*_G_ is correlated with defects and inversely correlated to lateral size, while an increase in *I*_2D_/*I*_G_ is usually caused by exfoliation.

CPN produced unstable dispersions that sedimented within an hour, leaving a clear solvent (Figure 4c). This was also confirmed by Raman analysis: the sample data mostly overlaps the gray shadow denoting the starting graphite, showing little change in the material (Figure 4b). In contrast to CPN, DMI and PRL produced visually black dispersions (Figure 4c) albeit of differing concentration, as measured by UV–vis spectroscopy (Figure S5.4), which remained stable beyond a month.

DMF successfully exfoliated graphene and produced a stable dispersion (visible solution opacity, Figure 4c), although much of the material remained similar to the starting graphite. DMI produced a dispersion with a slightly higher concentration of graphene and more evidence of exfoliation, particularly seen in the Raman data (Figure 4b). However, both DMF and DMI were found to be less effective than the traditionally used NMP. Overall, NMP and PRL are equally effective at stabilizing the exfoliation process, producing stable black dispersions with the highest graphene concentrations (Figure 4c). Their Raman spectra (Figure 4b) showed the largest shift of the histogram away from the starting graphite (graphite shown as the gray shadow). Since this heat map shows the distribution of Raman metrics measured from each material, the change in the histogram to greater occupancy (lighter color) of bins corresponding to higher *I*_2D_/*I*_G_ values indicates a statistically significant change in the spectral data between the two samples. In this case the increase in *I*_2D_/*I*_G_ implies a decrease in the flake thickness that is most impactful for the PRL and NMP exfoliated graphene.^23^ Furthermore, the PRL histogram occupies a smaller area caused by a decreased polydispersity of spectra obtained, indicating more uniform flakes^22^ (see Supporting Information Section 5.2 for a detailed discussion). The lower *I*_D_/*I*_G_ is likely caused by larger flakes within the sample, and this is supported by the microscopy data of the few layer flakes (Figure S5.3, Supporting Information Section 5.3).^24^

**Figure 5:**
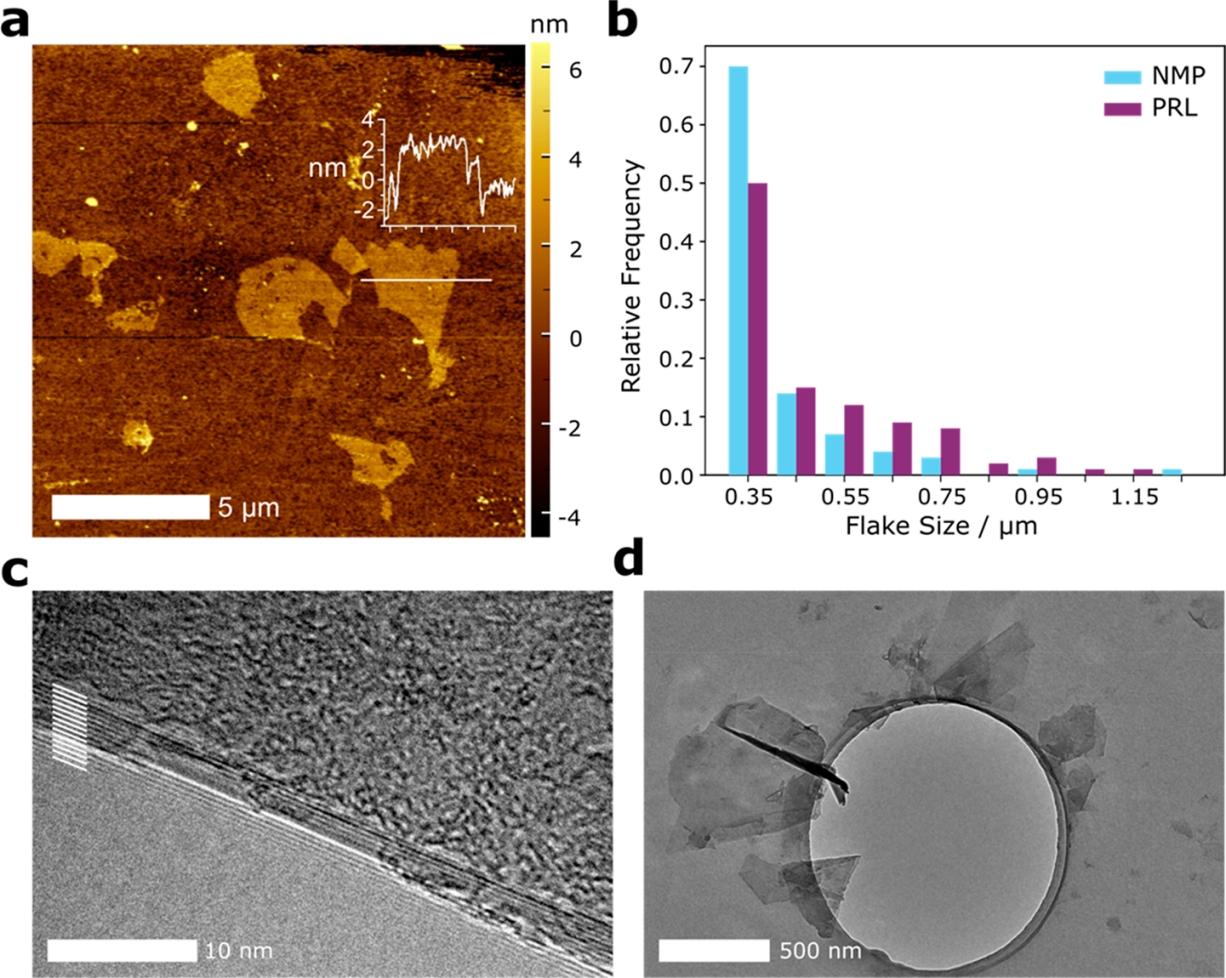
Microscopy analysis of graphene flakes produced with PRL. (a) AFM micrograph of exfoliated graphene flakes deposited on silicon, insert: height profile along white line shown with the same *x*-scale as the image scale. (b) Flake lateral size distribution, averaged from length and width,^25^ from TEM for NMP (blue) and PRL (palatinate). (c) TEM micrograph of the folded edge of a thicker few-layer graphene flake with the 14 carbon layers visible and highlighted by white lines. (d) TEM micrograph of individual graphene flakes.

To further validate the exfoliation potential in PRL, we examined samples of its graphene dispersions with microscopy techniques. AFM showed large individual flakes micrometers in lateral size and 1–2 nm in thickness (Figure 5a, Supporting Information Section 5.5), indicating the presence of single and few-layer graphene. TEM was used to image a large number of flakes, some examples shown in (Figures 5c,d and Supporting Information Section 5.6), and from these distributions comparing the lateral sizes of NMP and PRL exfoliated graphene were produced (Figure 5b). The flake size is the average of length and width. The larger flakes exfoliated in PRL were also detected with statistical Raman analysis (Figure 4b); the 3D histograms show the PRL exfoliated material has a lower *I*_D_/*I*_G_, a metric inversely correlated with lateral flake size.

### 2.5 Understanding Electronic Interactions with DFT

Our MD simulations highlighted the importance of solvent alignment on graphene surfaces for the successful stabilization of exfoliated layer. To characterize possible underlying chemical interactions, we profited from the statistical sampling provided by MD to serve as a starting point for an electronic structure study with planewave DFT.

To faithfully reproduce the experimental conditions of a solvated surface and to prevent finite size effects, we tested the dependence of the system charges on the size of included solvent shell. We looked for the shell size that no longer leads to the changes in Mulliken populations on the central solvent and surface beneath. The details of systems studied and their analyses are given in Supporting Information Section 4.1. We found that the population values converge only when a substantial solvent shell, with a cutoff radius between 8 and 9 Å, is included, which corresponds to 35–50 additional solvent molecules.

For each solvent system, we extracted the structure from MD simulation associated with the most probable configuration, as identified by our fingerprint analysis. The structures comprised of a central representative solvent, its underlying surface and a solvent shell within 9 Å. Each system featured a periodic surface in the *xy*-plane and a vacuum in the *z*-direction. The structures were geometry optimized before any further analysis (see the “Methods” section for details).

Previously, charge transfer between solvent molecules and the graphene sheet had been proposed to explain solvent stabilization of graphene.^26^ We assessed charge distribution via Mulliken population analysis but were unable to find any conclusive evidence. We continued with a range of additional analyses: solvent-induced changes in the layer undulation and *d*-spacing, changes in the Hirshfeld populations, *xy*-averaged electron density per graphene layer, partial electrostatic potential, changes of charges per molecule and surface area, as well as density of states (complete results data in the Supporting Information Section 4.3). Although the density of states analysis highlighted slight differences between the partially occupied bands of effective and ineffective solvents; these differences were too small to be considered statistically significant. We note that graphene layers can distribute charges across the plane of their surface. Thus, even if some charge transfer is present, its identification may not be possible within the level of statistical error.

## 3 Discussion and Conclusion

The utility of molecular modeling for the characterization and prediction of solvent–material interactions has been demonstrated on an example of solvent-mediated graphene exfoliation. We used MD simulations to examine an array of solvents and to classify their interactions with the substrate, obtaining a distinctive molecular fingerprint associated with these solvents’ effectiveness. Interestingly, statistically inferred representative structures examined via planewave DFT showed no significant electronic structure changes, failing to confirm previously suggested charge-transfer mechanisms.^26^ Our DFT study featured a number of extremely large systems, allowing us to minimize finite-size effects and undersampling and, therefore, reduce the risk of false-positive conclusions.^27,28^ The lack of relevant changes in the electronic structure of graphene and adsorbed solvent molecules within our calculations is of note, indicating that physisorption dominates chemisorption.

Statistical ensembles from MD simulations were able to highlight a key interaction shared by all effective solvents tested–formation of a structured solvent layer at the interface with graphene, where the solvent’s π-system over part of the ring aligns parallel to the surface. The saturated section of the ring is, therefore, pushed away from the surface and acts as a steric barrier. This prevents another π-system from establishing close-contact with the surface and restacking. Solvents without π-electrons are ineffective, as they only weakly interact with the graphene, maintaining the flexibility to reorient themselves and allowing for restacking. Planar conjugated molecules can also allow for the restacking of the graphene sheets, as they remain flat on the surface. This key interaction was shared by all the effective solvents tested within this work, but it is possible, and indeed likely, that other adsorption modes exist from other solvent molecules within the vast possible chemical space.

While previously Hansen solubility parameters and surface tensions have been used to guide the choice of new solvents, the predictive power of the method is limited since it is based on solvent–solvent mixing which ignores heterogeneous interactions at the interface. Our observations suggest that DLVO-based approaches,^29−31^ developed for colloid suspensions, to be better poised, as they include forces between interfaces such as solid surfaces and liquid films. Our work has highlighted the importance of accounting for interfacial interactions to describe the solvent-nanosheet stabilization mechanism. Furthermore, it should be highlighted that other combinations of 2D materials and solvents should benefit from such an *in silico* approach as a part of assessment for nanomaterial processability.

Overall, we demonstrated how our modeling pipeline was able to identify key molecular interactions driving graphene stabilization, informing the identification of an effective solvent, 2-pyrrolidone, which was also experimentally validated. Our computational approach is of general applicability for the systems where physicochemical interactions of liquid or gas with a surface result in a macroscopically observable phenomenon. In this context, we foresee utility of our approach for examining other 2D materials, such as MXenes, transition metal dichalcogenides, and layered double hydroxides.

## 4 Methods

### 4.1 Molecular Dynamics Simulations

#### 4.1.1 Set Up and Simulations

MD simulations featured 4.2 × 4.2 nm^2^ graphene or graphite surfaces, where graphite is formed by a stack of six graphene sheets. The material layer is placed at center of the simulation box of 10 nm, filled with solvent molecules. See Supporting Information Section 2.2 for full details of the set up. All the simulations were performed using CHARMM36^32^ force field, assigned with CGenFF.^33,34^ Parameters with a high assignment penalty score have been reassigned and thoroughly validated; see Supporting Information Section 2.1 for further details.

The simulations were performed with GROMACS 2018.^35^ Each simulation was first energy-minimized using a steepest descent algorithm with convergence criterion being the maximum force on any one atom to be less than 100 kJ mol^–1^ nm^–1^. Systems were then equilibrated for 5 ns in the isothermal–isobaric ensemble with a velocity-rescale thermostat set at 300 K, coupling constant set to 0.1 ps, and a semi-isotropic Berendsen barostat at 1 bar, coupling constant of 1 ps. The minimization and equilibration simulations were run with real-space particle-mesh-Ewald electrostatics and a van der Waals cutoff of 1.2 nm. After equilibration, production runs of 20 ns were performed, using the same protocol except with a longer cutoff of 1.4 nm.

The simulations were assessed for convergence using *DynDen*(36) (see Supporting Information Section 2.3), and the last 10 ns of the trajectory were used for further analysis. We then followed previously established analysis protocols, obtaining linear partial density, layer undulations, and interlayer spacing (Figures S2.4, S2.5).^37^ See Supporting Information Section 2 for the full details of preparation and validation systems for MD simulations.

#### 4.1.2 Analysis of Solvent Alignment and Selection of Representative Molecular Clusters

The coordinates of all solvent atoms required by our orientation descriptors are extracted from simulations and saved into a file using a VMD Tcl script.^38^ These coordinates are processed with a Python script to extract orientation information for each molecule at each time frame via vector and plane normal in spherical coordinates as well as solvent molecule average *z*-position. From this data, we generated 2D histograms reporting on the distribution of each orientation descriptor (elevation and azimuth of vector and plane normal) versus its position on the *z*-axis (Figure S3.2). We then extracted the subset of descriptors belonging to surface coating, or adsorbed, molecules which lay within 0.6 nm from the surface, as determined from the linear density profile (Figure S2.4). Using the *k-means* clustering algorithm, we aggregated molecules into 12 clusters according to the surface interaction descriptors (Figure S3.3). We selected the molecules associated with the centroid of each cluster as representative of typical solvent absorption modes, their selection included the molecule itself, additional surrounding solvent molecules with any atom falling within 9 Å from the representative, solvent molecule, and surface patch underlying it. For graphite, the selected patch included all six underlying layers, while for graphene, solvent molecules underneath the layer were also included. All systems occupied a simulation box of ∼2.3 × 3.1 × 3.4 nm^3^, which also allowed for vacuum in *z*-direction. Graphene systems featured between 500 and 700 atoms, out of which 252 formed the graphene sheet. Graphite systems contained 1510 atoms of material, with solvent molecules totalling to 1650 and 1800 atoms. The selected cluster IDs, as well as angles of vector and plane elevation are given in Table S3.4. See Supporting Information Section 3 for full details of analysis of MD simulations.

### 4.2 Density Functional Calculations

The electronic structure calculations were performed using the CASTEP code (v18).^39^ The exchange-correlation interactions were described within the generalized gradient approximation of Perdew, Burke, and Ernzerhof^40^ with the semiempirical correction of Grimme for dispersion used in geometry optimizations.^41^ Ultrasoft/PAW potentials were used to describe the valence electron interactions with atomic cores. A supercell approach was used with periodic boundary conditions with a vacuum region to simulate the surfaces. Brillouin zone sampling and plane-wave basis set cut-offs were set to converge total energy differences to better than 0.01 eV/cell and SCF and eigenenergy convergence tolerance of 10^–8^ eV. An L-Broyden–Fletcher–Goldfarb–Shanno minimization scheme was used for geometry optimization, which is considered converged when the following criteria are satisfied: electronic energy tolerance of 1 × 10^–6^ eV, energy change 5 × 10^–6^ eV per atom, maximum displacement of 5 × 10^–4^ Å, and maximum force of 3 × 10^–2^ eV Å^–1^.

After geometry optimization, atomic charges of the models were calculated *via* Mulliken population analysis and Hirshfeld analysis.^42^ The valence electron density of states was calculated, and partially occupied bands shown on the structure. The data was postprocessed with *c2x*(43) and rendered with VMD.^38^ Furthermore, layer undulation and interlayer spacing was analyzed using the same protocol as for MD simulation (Figure S4.2). See Supporting Information Section 4 for full details of analysis of DFT simulations.

### 4.3 Experimental Section

#### 4.3.1 Graphene Exfoliation

Graphene was exfoliated following the procedure outlined by Paton et al.^7^ with a Silverson Ltd. L5M laboratory mixer with a 15.6 mm diameter rotor/stator with a gap size of 100 μm. Natural flake 325-mesh graphite (50 mg mL^–1^) was added to 100 mL of solvent and mixed at 6000 rpm (γ̇ = 49000 s^–1^) for 60 min. The resulting dispersion was centrifuged at 5000 rpm (3438 g) for 60 min to remove large unexfoliated flakes.

#### 4.3.2 Laboratory Characterization

##### 4.3.2.1 Raman Spectroscopy

Raman spectra were recorded with a Horiba LabRam Evolution using a 532 nm, 1 mW laser and a 50× long working distance objective lens. The instrument was calibrated against the 520.7 cm^–1^ Raman signal of silicon. Dispersions were filtered through a 0.45 μm PTFE filter membrane to produce a smooth surface from which Raman spectra were collected directly. Over a hundred points were collected from each sample, these spectra were fitted with a six-order polynomial background and Lorentzian line shapes using Python 3.4; further method details published previously.^22^ Further detailed information and a complete data set are given in Supporting Information Section 6.

##### 4.3.2.2 Transmission Electron Microscopy (TEM)

TEM images were acquired using a JEOL 2100F FEG TEM operating at 80 kV. Samples were dispersed in ethanol solution then dropped onto holey carbon on a 300-mesh copper grid. Flake lateral size measurements were made following the National Physical Laboratory protocol, calculating a mean of the longest length and perpendicular width value.^25^

##### 4.3.2.3 Scanning Electron Microscopy (SEM)

SEM images were collected with a Hitachi SU-70 FEG SEM. Samples were dispersed in ethanol and deposited on silicon wafers, and all images were collected uncoated.

##### 4.3.2.4 Atomic Force Microscopy (AFM)

AFM images were collected with an AIST-NT SPM SmartSPM-1000 operating in noncontact mode with silicon tips; samples were washed in ethanol by repeated dispersion and centrifugation before being drop-cast onto silicon for imaging.

##### 4.3.2.5 Ultraviolet–Visible–Near-Infrared Spectroscopy (UV–vis–nIR)

UV–vis spectra were collected with a Cary 5000 UV–vis–nIR using quartz cuvettes, path length 2 mm.

##### 4.3.2.6 Photographs

Photographs (Figure 4c) were taken following shear mixing and centrifugation as described above, the dispersions were then left for 6 days before photographing with no visible sign of instability.

See Supporting Information Section S5 for full details of experimental preparation and characterization, including complete set of microscopy images.

### 4.4 Data Visualization

Simulation box structures were rendered with VMD v.1.9^38^ with carbon atoms in gray, oxygen atoms in red, nitrogen atoms in blue, sulfur atoms in yellow, and hydrogen atoms in white, unless otherwise specified. All graphs reporting on simulation data, as well as on statistics of Raman spectra were produced with Python 3.4 using the *matplotlib* package.^44^

## Supporting Information Available

Data tables of solvents, details and results from MD simulations, DFT simulations, and further experimental results and characterization (PDF)

## Abbreviations

NMP: *n*-methyl-pyrrolidone
MD: molecular dynamics
DFT: density functional theory
DMF: *N*,*N*-dimethylformamide
DMSO: dimethyl sulfoxide
DMI: 1,3-dimethyl-2-imidazolidinone
PRL: 2-pyrrolidone
CPN: cyclopentanone
AFM: atomic force microscopy
SEM: scanning electron microscopy
TEM: transmission electron microscopy
