# Supplementary material for: Identification of Graphene Dispersion Agents through Molecular Fingerprints
Source: ACS Nano. 2022 Sep 27;16(10):16109–17. doi: 10.1021/acsnano.2c04406 (PMC9620402; doi:10.1021/acsnano.2c04406)
Supplement: Supplementary file 1 — nn2c04406_si_001.pdf [file nn2c04406_si_001.pdf]

Identification of graphene dispersion agents through molecular fingerprints

Stuart J. Goldie[1†], Matteo T. Degiacomi[2], Shan Jiang[1], Stewart J. Clark[2], Valentina Erastova[3*], Karl S. Coleman[1*‡].

AUTHOR ADDRESS

1. Department of Chemistry, Durham University, South Road, Durham, DH1 3LE, UK
2. Department of Physics, Durham University, South Road, Durham, DH1 3LE, UK
3. School of Chemistry, University of Edinburgh, David Brewster Road, Edinburgh, EH9 3FJ, UK

KEYWORDS: graphene, 2D materials, exfoliation, molecular modelling, solvent prediction

Supplementary Information

Table of Content

Identification of graphene dispersion agents through molecular fingerprints 1
1. Solvents used in this study 3
  1.1 Bulk properties of solvents used in this study 3
  1.2 Predicted solvent effectiveness from Hansen solubility parameters 5
2. Molecular dynamics simulations 9
  2.1 Force field validation 9
  2.2 Solvent system set up 10
  2.3 Assessing simulation convergence 12
  2.4 Linear density profiles 15
  2.5 Layer undulation and interlayer spacing 16
3. Structural characterisation pipeline 17
  3.1 Descriptors 17
  3.2 Alignment of solvent molecules as a function of distance from the surface 18
  3.3 Clustering of the surface adsorbed molecules 23
  3.4 Representative structures from MD from DFT 28
4. Planewave density functional theory calculations 29
  4.1 Test of solvation shell size 29
  4.2 Layer undulation and interlayer spacing from DFT 35
  4.3 Density of States 36
5 Experimental 42
  5.1 Sample preparation 42
  5.2 Raman 42
  5.3 Flake size 43
  5.4 Absorbance and concentration 44
  5.5 AFM 45
  5.6 TEM 47

6. Validating Number of Raman Data Points 51
6.1 Graphite 51
6.2 CPN 53
6.3 DMF 55
6.4 DMI 57
6.5 NMP 59
6.6 PRL 61
References 63

1. Solvents used in this study

1.1 Bulk properties of solvents used in this study

Table S1.1: Physical properties of the solvents in the study, *taken from ChemSpider.*

SOLVENT	Structure	Molecular Weight, *g mol^{-1}*	Specific Gravity, *g mL^{-1}*	Boiling point, *^{o}C*
DMF<br>Dimethylformamide	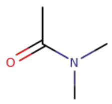	73.09	0.944	153
NMP<br>N-Methylpyrrolidone	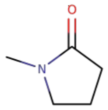	99.13	1.03	204
DMSO<br>Dimethyl sulfoxide	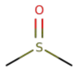	78.13	1.10	189
ETH<br>Ethanol	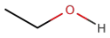	46.07	0.789	78
ACT<br>Acetone	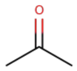	58.08	0.791	56
H2O<br>Water	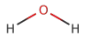	18.01	1.00	100
PRL<br>2-Pyrrolidone	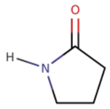	85.10	1.12	245
DMI<br>1,3-Dimethyl-2-imidazolidinone	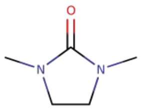	114.15	1.056	225
CPN<br>Cyclopentanone	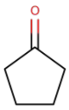	84.12	0.949	130

Table S1.2: Hansen and Hildebrand solubility parameters and surface tensions for solvents used in the study and graphene*. Hansen parameters: δ_d is dispersion solubility, δ_p is polar solubility, and δ_h is hydrogen bonding solubility. Hildebrand solubility parameter is δ_t.*

**SOLVENT**	**Solubility Parameters**				**Surface tension,** *mJ m⁻²*
	**Hansen**			**Hildebrand**	
	δ_d, $MPa^{1/2}$	δ_p, $MPa^{1/2}$	δ_h, $MPa^{1/2}$	δ_t, $MPa^{1/2}$	
DMF<br>Dimethylformamide	17.4	13.7	11.3	24.9	34.4
NMP<br>N-Methylpyrrolidone	18.0	12.3	7.2	23.0	44.6
DMSO<br>Dimethyl sulfoxide	18.4	16.4	10.2	26.7	42.9
ETH<br>Ethanol	15.8	8.8	19.4	26.5	22.1
ACT<br>Acetone	15.5	10.4	7.0	19.9	23
H_2O<br>Water	15.5	16.0	42.3	47.8	72.7
PRL §<br>2-Pyrrolidone	19.4	17.4	11.3	28.4	32.5 *
DMI<br>1,3-Dimethyl-2-imidazolidinone	18.0	10.5	9.7	23.0	33.6 *
CPN<br>Cyclopentanone	17.9	11.9	5.2	22.1	33.0 ⁺
Graphene #<br>*desired solvent parameters*	***18***	***9.3***	***7.7***	***23***	***40***

Data for solubility parameters taken from HSPiP software www.hansen-solubility.com, except § (data from [1]) and # (parameters estimated by [2]).

Surface tension data from [3], except * (predicted data generated using the ACD/Labs Percepta Platform - PhysChem Module) and ⁺ (data from [4]).

Table S1.3: Toxicity and hazard information of key solvents tested. *The key hazards associated with the experimentally tested solvents taken from safety data sheets.*

SOLVENT	Hazards<br>1 (greater) – 4 (lesser)
DMF<br>Dimethylformamide	Reproductive Toxicity: *Cat 1B*<br>Eye Irritation: *Cat 2*<br>Flammable: *Cat 3*<br>Acute Toxicity: *Cat 4*
NMP<br>N-Methylpyrrolidone	Reproductive Toxicity: *Cat 1B*<br>Eye Irritation: *Cat 2*<br>Skin Irritation: *Cat 2*<br>Organ Toxicity: *Cat 2 & 3* (2 - Respiratory system 3 - Kidney, Liver, spleen, Blood)<br>Flammable: *Cat 4*
PRL<br>2-Pyrrolidone	Reproductive Toxicity: *Cat 1B*<br>Eye Irritation: *Cat 2*
DMI<br>1,3-Dimethyl-2-imidazolidinone	Eye Damage: *Cat 1*<br>Reproductive Toxicity: *Cat 2*<br>Acute Toxicity: *Cat 4*
CPN<br>Cyclopentanone	Skin Irritation: *Cat 2*<br>Eye Irritation: *Cat 2*<br>Flammable: *Cat 3*

1.2 Predicted solvent effectiveness from Hansen solubility parameters

A basic consideration of solubility is that dissolving one material in another means breaking the bonds that exist between the identical molecules in each pure material and replacing them with new interactions between the different molecules in the solution. If these new interactions are similar in energy to the broken bonds in the pure form, the solution will be stable but if the energy cost of disrupting the pure phases is not balanced the solution or dispersion will be unstable. A simple solubility parameter is the Hildebrand parameter, this is simply the square root of the cohesive energy density:

$$\delta_t = \sqrt{E_{coh}/V}.$$

Cohesive energy density is the energy, E_{coh}, required to completely remove a unit volume, V, of material from all neighbours. Materials with similar solubility parameters are, therefore, predicted to form favourable interactions.

The primary weakness of the Hildebrand parameter is its inability to account for different types of interaction that may have similar energies, but behave very differently and would not be compatible. Splitting the cohesive energy density into polar, dispersive and hydrogen bonding components (δ_p, δ_d and δ_h respectively) produces the Hansen solubility parameters for a material:

$${\delta_t}^2 = {\delta_d}^2 + {\delta_p}^2 + {\delta_h}^2 .$$

The closer all the Hansen parameters are collectively, between the solute and solvent, the greater the solubility. This approach has been applied to a huge range of systems; however, its limitations must be considered. Firstly, Hansen parameters are difficult to measure and rely on comparisons with other known solvents. The model is also unable to consider the effect of shape and size, and most significantly does not include contributions from electrostatics and induced structuring of solvents around solutes.[5]

The derivation of the Hansen parameters of graphene used solvents already known to be effective, taking an averaged value of the solvents normalised to their dispersion performance. [2] These Hansen parameters from graphene can then be compared with other solvents to predict the solvent performance, Ra, by calculating the distance between the solvent and graphene in 'Hansen space', with a larger weight given to the dispersive forces:

$$Ra^2 = 4\left({\delta^{sol}}_d - {\delta^{gra}}_d\right)^2 + \left({\delta^{sol}}_p - {\delta^{gra}}_p\right)^2 + \left({\delta^{sol}}_h - {\delta^{gra}}_h\right)^2.$$

Undertaking this for the solvents tested in this work reveals no correlation between the Hansen parameters and solvent performance, as shown in **Figure S1.1**. It should be noted only solvents with measurable graphene concentration in dispersion are shown, i.e. other very poor solvents such as water are not shown. The calculated Ra values for NMP and PRL would suggest a dramatic difference in graphene dispersion performance. However, our theoretical and experimental work has shown that these solvents are competitive. While Hansen parameters can be calculated for new materials and used as an approximate guide to solvent performance their key limitations must be considered. Firstly, as shown in this case they cannot account for structures forming around the surfaces so may give erroneous results for highly structured, and therefore effective, solvents. The other limitation that must be considered, especially for new materials, is the large amount of experimental work required to measure the performance of every new material in a vast catalogue of different solvents to ensure an accurate empirical measurement of the Hansen parameters for that material. Only after this data set has been measured can any predictions even be attempted using Hansen parameters.

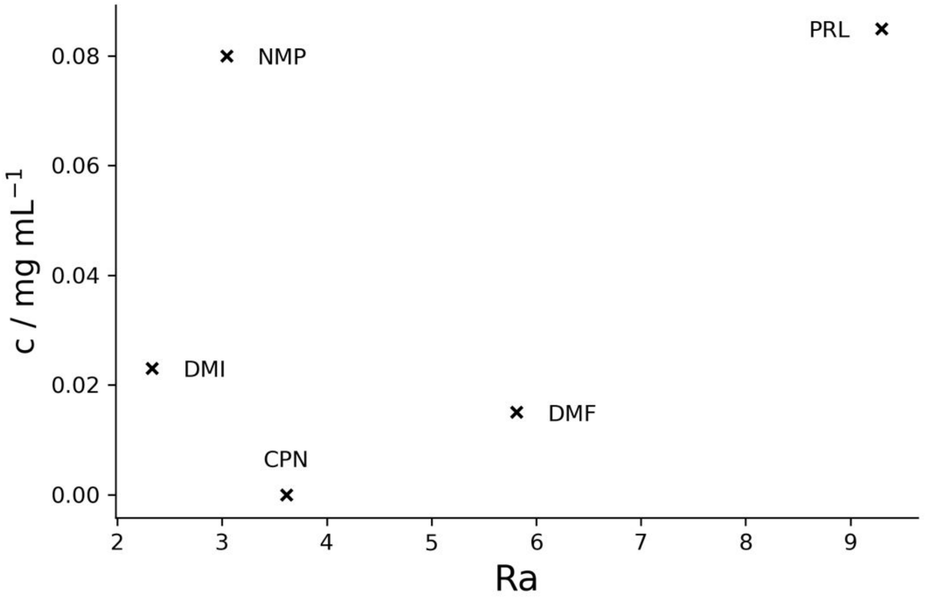

Figure S1.1: *The theoretical solvent compatibility according to Hansen solubility parameters (Ra) compared with the experimental concentration of graphene dispersions prepared in this work.*

1.3 Comparison with previously reported solvents

Table S1.4 summarises the experimental conditions and resulting graphene concentrations from other shear exfoliated samples in a collection of literature studies. It can be seen that a great variation exists between the different experiments. For brevity we do not include the details of graphene exfoliated by other methods like ultrasonication. While the exfoliation process does not alter the stability of the resulting dispersion in different solvents, the concentration obtained tends to be greater with sonication but the volumes processible are smaller. The concentrations reported within this work (given in bold in the Table S1.4) are comparable to other studies using similar shear exfoliation procedures, however no two studies use identical process of both shear mixing and centrifugation; this makes any direct comparisons of concentration meaningless.

Table S1.4: Comparison of graphene dispersions prepared by mechanical shear mixing. Summary of graphene concentrations and experimental conditions under which they were prepared, including centrifugation of the final dispersion. It was not possible to calculate the shear rate for every case due to missing rotor-stator dimensions. Abbreviations of solvents: NMP = 1-Methyl-2-pyrrolidinone, DMF = N-N-Dimethylformamide, PVA = Poly(vinyl alcohol), CHP = N-cyclohexyl-2-pyrrolidone, IPA = Isopropyl Alcohol, PVP = Poly(vinyl pyrrolidone), SDS = odium Dodecyl Sulfate, PG = Propylene glycol, BB = Benzyl benzoate, DMI = 1,3-dimethyl-2-imidazolidinone, PRL = 2-pyrrolidone.

SOLVENT	EXFOLIATION PARAMETERS	SHEAR RATE / s^{-1}	SHEAR TIME / hours	CENTRIFUGE PARAMETERS	INITIAL CONC / mg mL^{-1}	FINAL CONC / mg mL^{-1}	% YIELD	REF	NOTES
Cyrene	Silverson LM5 Mixer Emulsor Screen (7.5k rpm) Emulsor Screen and Axial Flow head (5k rpm)	95200, 63467	8, 2	9000 rpm 40 min	50	2.24	4.48	[6]	Shear rate assuming "standard" large rotor of 32 mm diameter
NMP					50	0.44	0.88		
DMF					50	0.20	0.40		
PVA	Silverson LM5 Mixer 1 hr, 4500 rpm	49645	1	500 rpm 40 min	50	0.011	0.022	[7]	
NMP		49645	1		50	0.05	0.10		
CHP		49645	1		50	0.085	0.17		
Sodium Cholate	Silverson LM5 Mixer 2600 rpm	32269	1		50	0.005	0.010		5 mg mL^{-1} surfactant
NMP	Taylor–Couette Flow 1500 rpm	-	1	1000 rpm 30 min	50	0.65	1.3	[8]	
IPA-H_2O	Fluko FM300 9500 rpm	-	1	450 rpm 45 min	10	0.18	1.8	[9]	
1-BuOH	Silverson LM5 Mixer 4500 rpm	57100	$0.\dot{6}$	226 g 150 min	50	0.023	0.046	[10]	Shear rate assuming "standard" large rotor of 32 mm diameter
Sodium Cholate	IKA Magic Lab (1L module micro-plant) 20000 rpm	-	2	4700 rpm 60 min	40	0.68	1.7	[11]	3 mg mL^{-1} surfactant
PVP		-			40	1.04	2.6		20 mg mL^{-1} surfactant
PVA	IKA T18 digital Ultra-Turrax disperser	-	1	500 rpm 45 min	10	3.36	33.6	[12]	1 mg mL^{-1} surfactant

	10000 rpm								
Sodium Cholate	POLYTRON® immersion dispersers PT 10–35 GT drive unit coupled with a PT-DA 20 16500 rpm	20950	2	1500 rpm 60 min	100	3	3	[13]	Range of values produced depending on starting graphite flakes, max value 3.9
SDS + EtOH (15 wt.%)	IKA Ultra-Turrax T-50	10472	1	1500 rpm 30 min	100	0.19	0.19	[14]	Higher concentration achieved by long mixing times
Modified PVA	IKA Ultra-Turrax T25 with a S25N - 25G-ST 12000 rpm	25133	$1.\dot{6}$	4000 rpm 60 min	25	1.04	4.16	[15]	8 mg mL^{-1} surfactant
NMP	Magic Lab, IKA 6000 rpm	-	6	3000 rpm 30 min	10	0.15	1.5	[16]	
NMP	Taylor–Couette Flow	20270	8	1500 rpm 120 min	10	0.122	1.22	[17]	Shear rate reported by authors
Fairy Liquid	Kenwood Kitchen Blender	28000	4	1500 rpm 45 min	100	0.92	0.92	[18]	12.5 mg mL^{-1} surfactant
NMP	Anton Paar MCR 301 Rheometer	3490	1	2500 rpm 60 min 2 x	50	0.002	0.004	[19]	
PG		3490	1		50	0.006	0.012		
BB		3490	1		50	0.001	0.002		
DMF	Silverson LM5 Mixer 4000 rpm	49645	1	5000 rpm 60 min	50	0.015	0.030	This Work	
NMP		49645	1		50	0.08	0.16		
DMI		49645	1		50	0.023	0.046		
PRL		46945	1		50	0.085	0.17		

2. Molecular dynamics simulations

2.1 Force field validation

All of the simulations were performed using CHARMM-36 forcefield,[20] assigned with CGenFF.[21] In the case of NMP, DMI, PRL and CPN some parameters held a high assignment penalty score, indicating poor analogy, and therefore required validation/optimisation. The penalties were related to the dihedrals and/or charge assignment, which was then refined using bond-charge increment rule[22] and with MATCH.[23] The refined force field parameters have been validated *via* MD simulation of a bulk solvent, measuring its bulk density.

Each simulation featured a cubic box, periodic in all directions, containing 729 molecules. Each simulation was first energy minimized using a steepest descent algorithm with convergence criterion being the maximum force on any one atom to be less than 100 kJ mol^{-1} nm^{-1}. The system was then equilibrated for 2 ns in the isothermal–isobaric ensemble at 300 K and isotropic 100 bar pressure to speed up volume reduction towards liquid state. Thus, was then followed by another 1 ns-long simulation at 300 K and 1 bar. In all the systems a velocity-rescale thermostat with a 0.1 ps coupling constant and Berendsen barostat with a pressure-coupling constant of 1.0 ps were used. The simulations were run with real-space particle-mesh-Ewald electrostatics and a Van der Waals cut-off of 1.2 nm.

Solvents' density was evaluated over the final 0.5 ns of the simulation, when box volume remained constant. **Table S2.1** shows the comparison against experimental density, with deviation below 4%.

Table S2.1: *Experimental and calculated density for parametrised molecules at 300 K and 1 bar. Experimental values taken from ChemSpider.*

Solvent	Experimental kg/m^3	Calculated kg/m^3	Deviation from experimental
CPN	945	913	3.4%
NMP	1024 – 1029	987	3.6 – 4.1%
PRL	1115	1070	4%
DMI	1052 – 1053	1036	1.5 – 1.6%

2.2 Solvent system set up

Our simulations featured approximately 4.2 × 4.2 nm^2 graphene and graphite surfaces. Graphite is a stack of six graphene sheets. The first layer, A, is followed by identical layer, B, separated by 0.335 nm and offset by $^{(a+b)}/_{3}$, where a and b are lattice translation vectors with respect to the first layer, A. This forms a AB-hexagonal stacking, as described by Bernal.[24] The layers are placed at centre of the simulation box and form a periodic xy-plane, see **Figure S2.1**. The simulation box was then extended along the z-axis, by an additional 8 nm to create a free volume of 125 nm^3. This volume is then filled with solvent molecules.

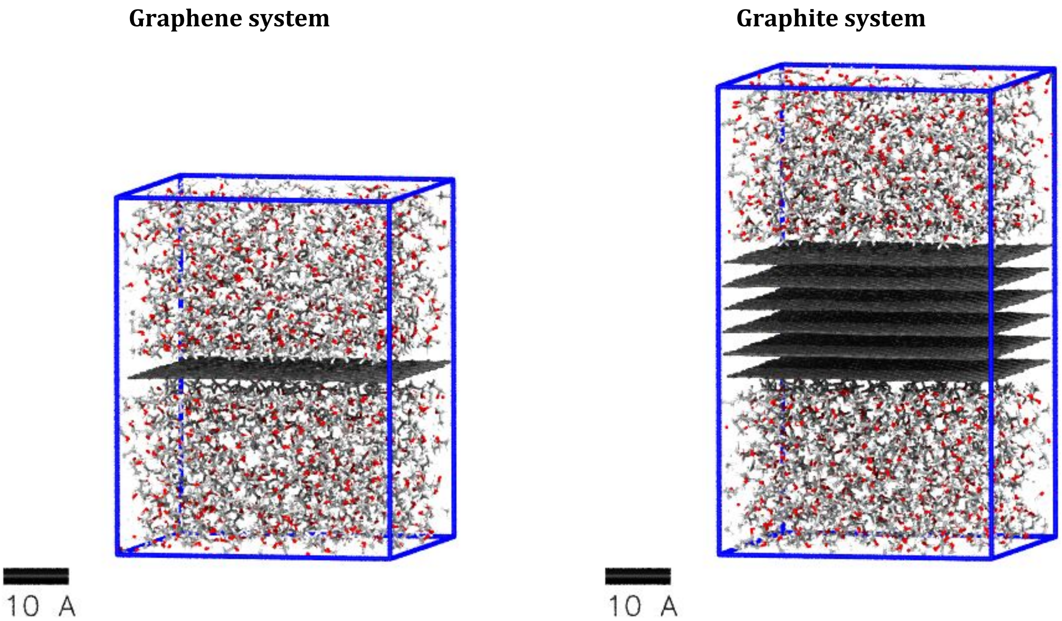

Figure S2.1: Example of systems simulated with MD, *featuring graphene (left) and graphite (right) in ethanol solvent. The carbon atoms of the material are represented as black spheres, while solvent carbons are shown in grey, oxygen in red and hydrogen in white. The image is rendered using VMD.*

The number of solvent molecules necessary to fill the space was calculated as follows:

$$N_{mol} = \frac{N_{Av} \times \rho \times V}{M_w},$$

where N_{Av} is Avogadro number, ρ is density, M_w is molecular weight and V is free volume. The number of solvent molecules inserted is given in the **Table S2.2.**

Table S2.2: Number of solvent molecules for each simulated system*. The number is equivalent when in presence of graphite or graphene.*

Solvent	No. molecules calculated	No. molecules inserted
DMF	976	1000
NMP	786	800
DMSO	1064	1000

ETH	1294	1000
ACT	1029	1000
H2O	4196	4000
PRL	994	800
DMI	700	700
CPN	853	800

The simulations were performed with GROMACS 2016.[25] Each simulation was first energy minimized using a steepest descent algorithm with convergence criterion being the maximum force on any one atom to be less than 100 kJ mol^{-1} nm^{-1}. The system was then equilibrated for 5 ns in the isothermal–isobaric ensemble with a velocity-rescale thermostat set at 300 K and the temperature coupling constant set to 0.1 ps. A semi-isotropic Berendsen barostat was used, set at 1 bar, with a pressure-coupling constant of 1 ps. The minimization and equilibration simulations were run with real-space particle-mesh-Ewald (PME) electrostatics and a van der Waals cut-off of 1.2 nm. After equilibration, a production run of 20 ns was performed. This was run with PME electrostatics and a Van der Waals cut-off of 1.4 nm in NPT ensemble, with the same parameters as in the equilibration.

2.3 Assessing simulation convergence

Simulation convergence was assessed through root mean square deviation (RMSD), showing that after ~10 ns RMSD have converged, i.e. flattened, indicating that after this time systems are in equilibrium. Furthermore, we have also checked for convergence using *DynDen.*[26]

DynDen analysis is shown on the Figure **S2.3A** for graphene and **2.3B** for graphite in ethanol, where evolution of linear density of the components (left columns) are assessed by the cross correlation (right columns). When there is no evolution is seen, the system is assumed to be converged.

For all the further analysis, the final 10 ns of the simulation are used.

Graphene systems **Graphite systems**

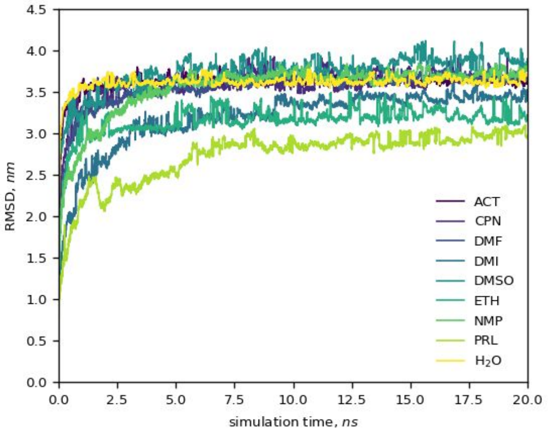

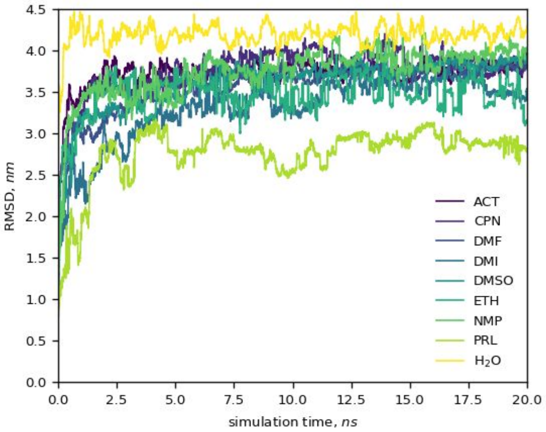

Figure S2.3: RMSD of solvents in our simulations. *Graphene systems shown on the left, graphite on the right.*

Graphene component

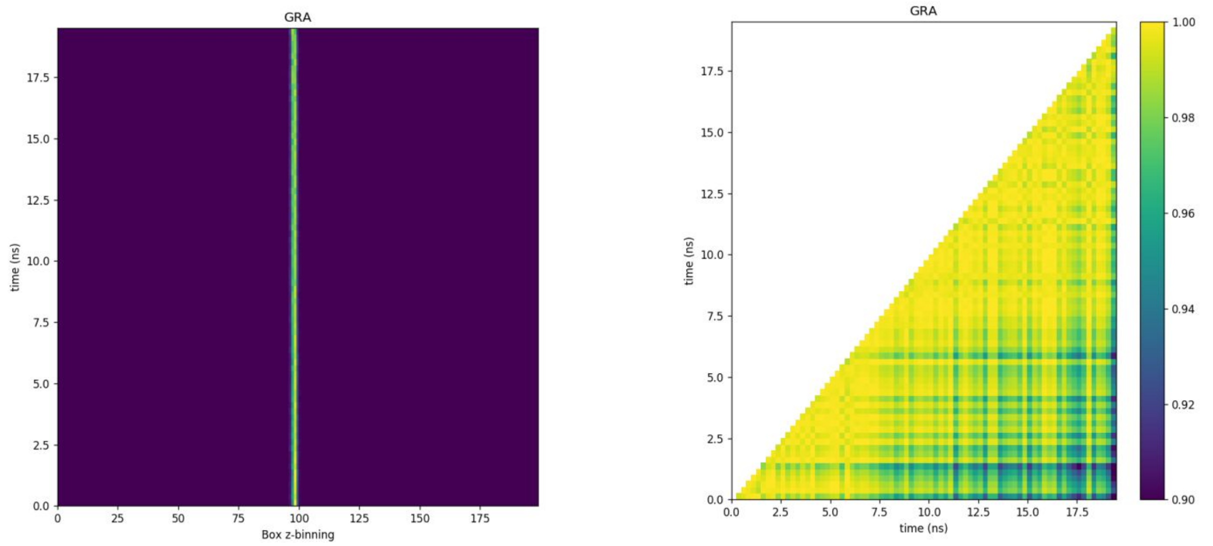

Ethanol component

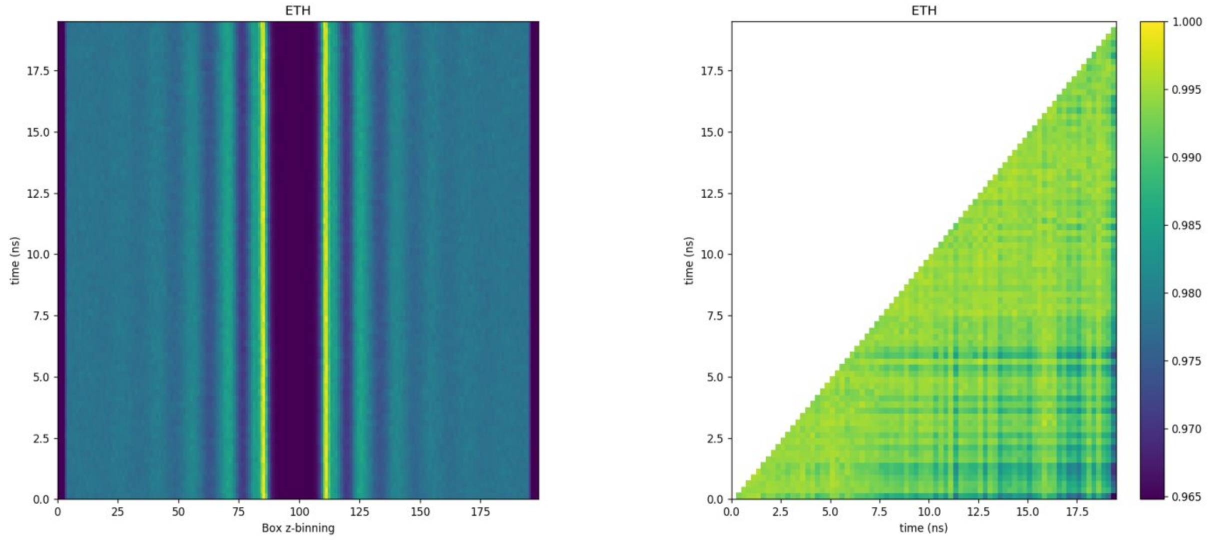

Figure S2.3A: DynDen analysis of graphene (GRA) in ethanol (ETH) *showing evolution of the linear density in the left column and cross-corelation in the right column.*

Graphite component

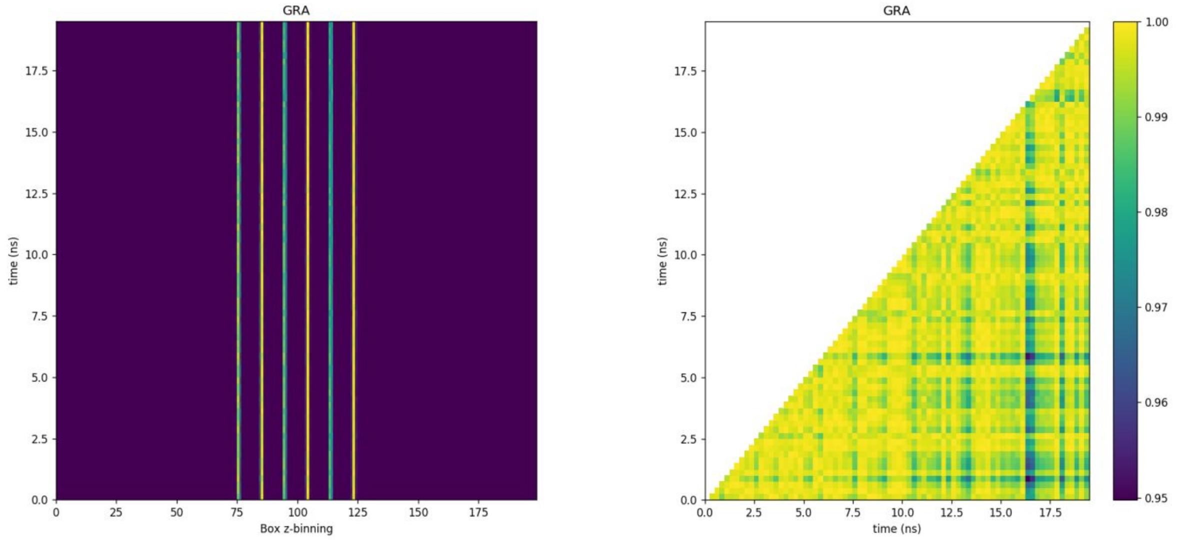

Ethanol component

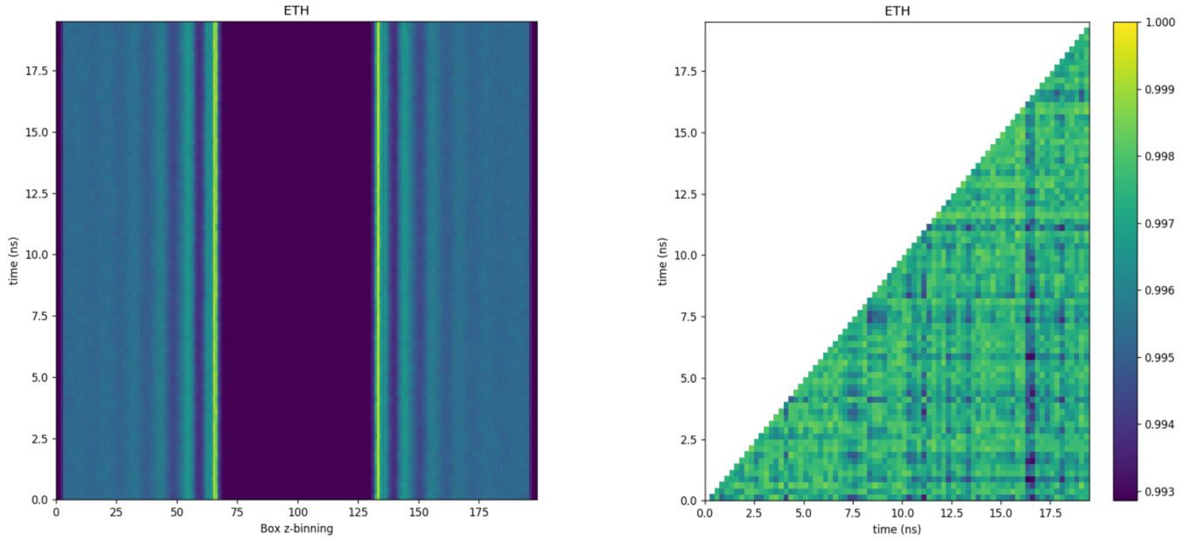

Figure S2.3B: DynDen analysis of graphite (GRA) in ethanol (ETH) *showing evolution of the linear density in the left column and cross-corelation in the right column.*

2.4 Linear density profiles

Linear, *xy*-averaged, density of solvents and graphene/graphite layers in the system, calculated with 500 slices along the *z*-axis. In all cases, the formation of multiple solvation shells is observed. Solvents behave comparatively when interacting with graphene or graphite.

Graphene systems **Graphite systems**

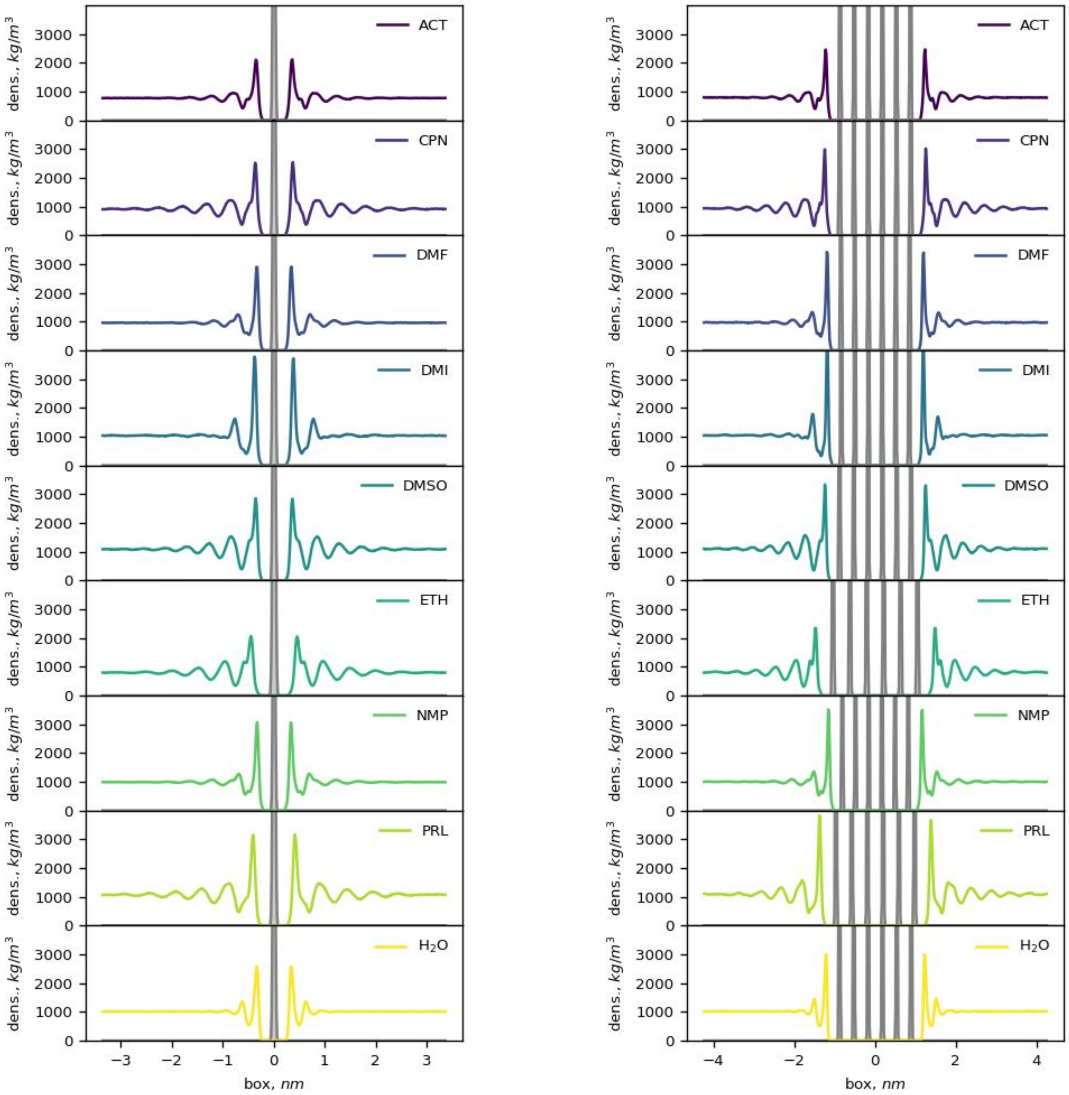

Figure S2.4: Linear densities of the components in graphene and graphite systems. *Gray lines represent the density of graphene (left) or graphite (right).*

2.5 Layer undulation and interlayer spacing

For each simulation, we calculate layer undulation, following previously established protocol,[27] where a layer is cantered at *z*=0 and standard deviation of atomic positions is shown as a bar, while the lowest and highest atom positions ever observed are shown as points.

Graphene appears marginally more flexible than graphite, although both only display sub-Ångstrom fluctuations. No solvent-specific effect to the layer undulation or d-spacing is observed.

Layer undulation for graphene and graphite in each solvent

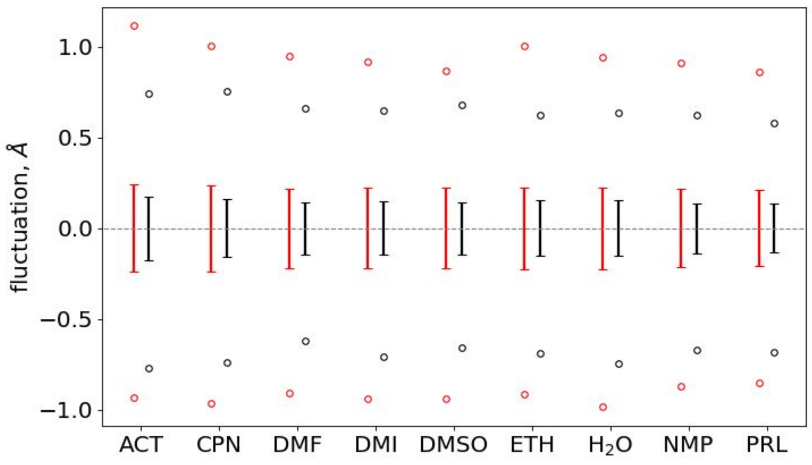

Interlayer spacing for graphite in each solvent

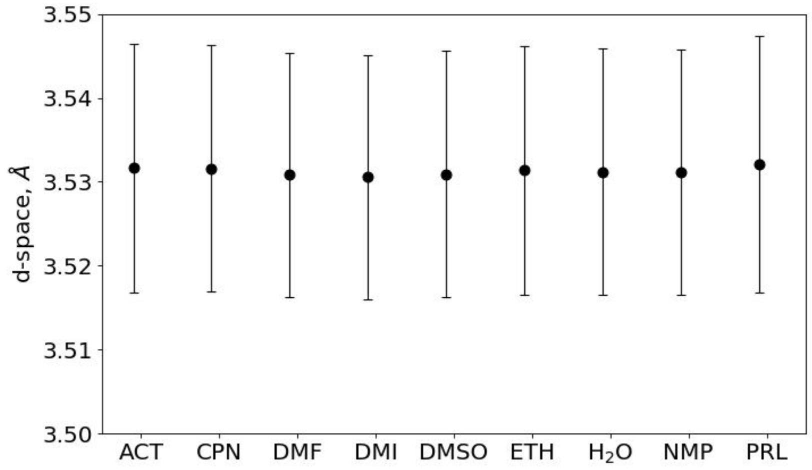

Figure S2.5: Layer undulations and interlayer spacing *shown as standard deviations, lowest and highest position of graphene layer (red) and graphite top layer (black) in the presence of solvents.*

3. Structural characterisation pipeline

3.1 Descriptors

To characterize the interactions of the solvent molecules with the material, we describe the orientation of each molecule with respect of the surface normal with a vector and a plane. The vector is located on a bond involving the most electronegative atom. The plane is defined by any three atoms such that, when possible, the vector is non-coplanar to it.

***Table S3.1**: Definition of vectors and planes for each solvent.*

**Solvent**	ACT	CPN	DMF	DMI	DMSO	ETH	NMP	PRL	H_2O	Definition<br>0° 90°
Vector	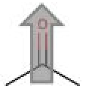	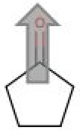	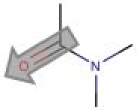	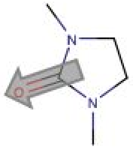	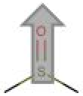	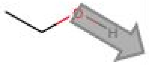	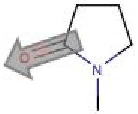	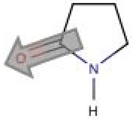	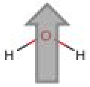	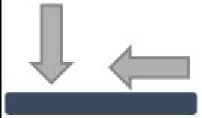
Plane	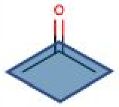	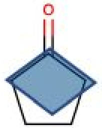	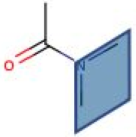	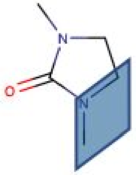	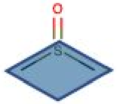	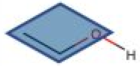	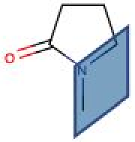	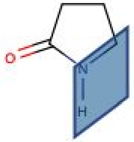	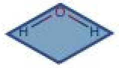	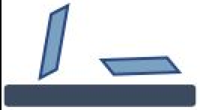

3.2 Alignment of solvent molecules as a function of distance from the surface

Material

Graphene | **Graphite**

SOLVENT

ACT

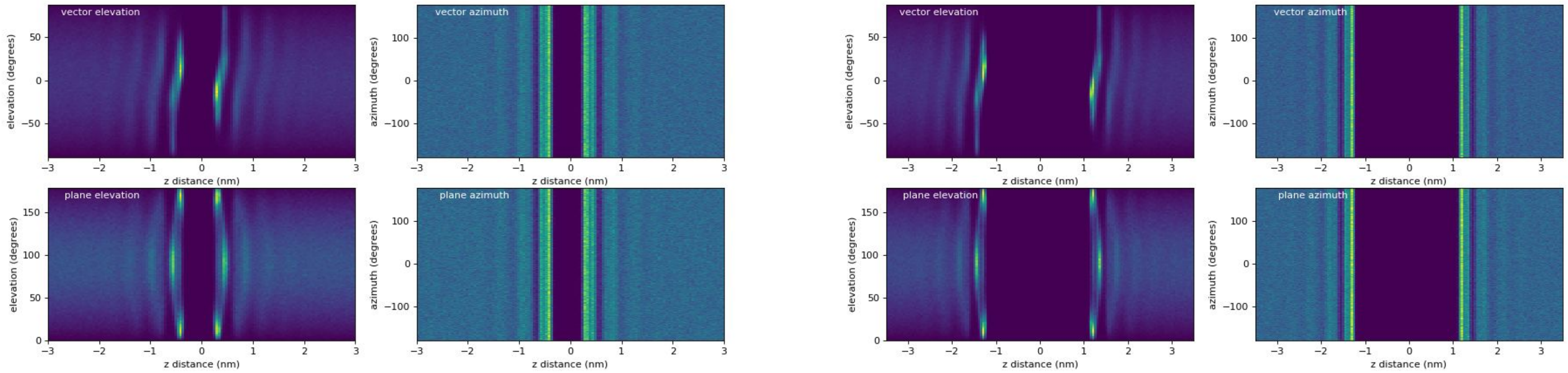

***Figure S3.2**: **orientation of solvents on graphene and graphite surfaces**. Orientations of vector and plane descriptors are reported as probability density in spherical coordinates, as a function of solvent distance from the surface. z=0 is set as the centre of graphene or graphite layers.*

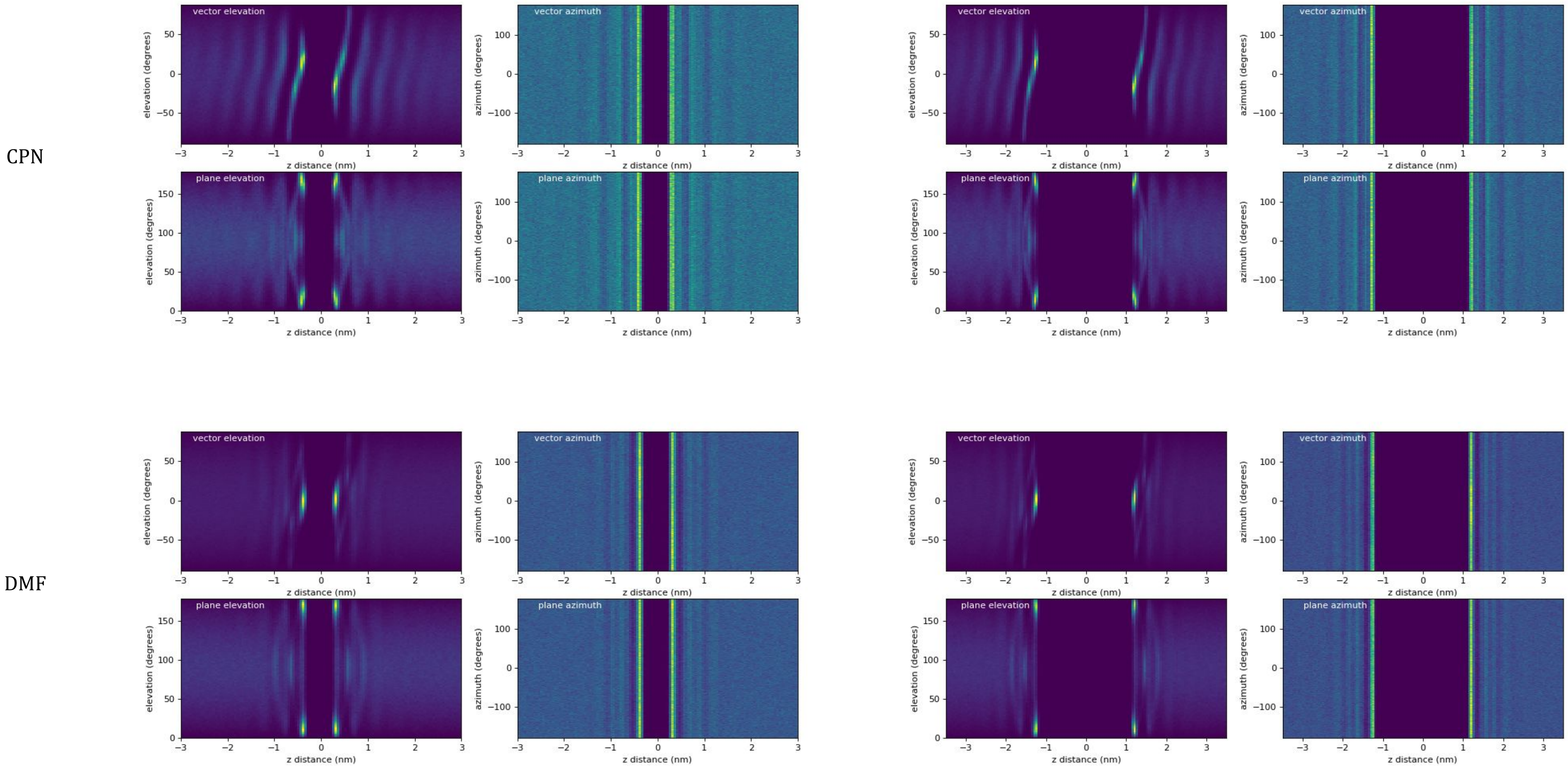

Figure S3.2: *contd.*

DMI

DMSO

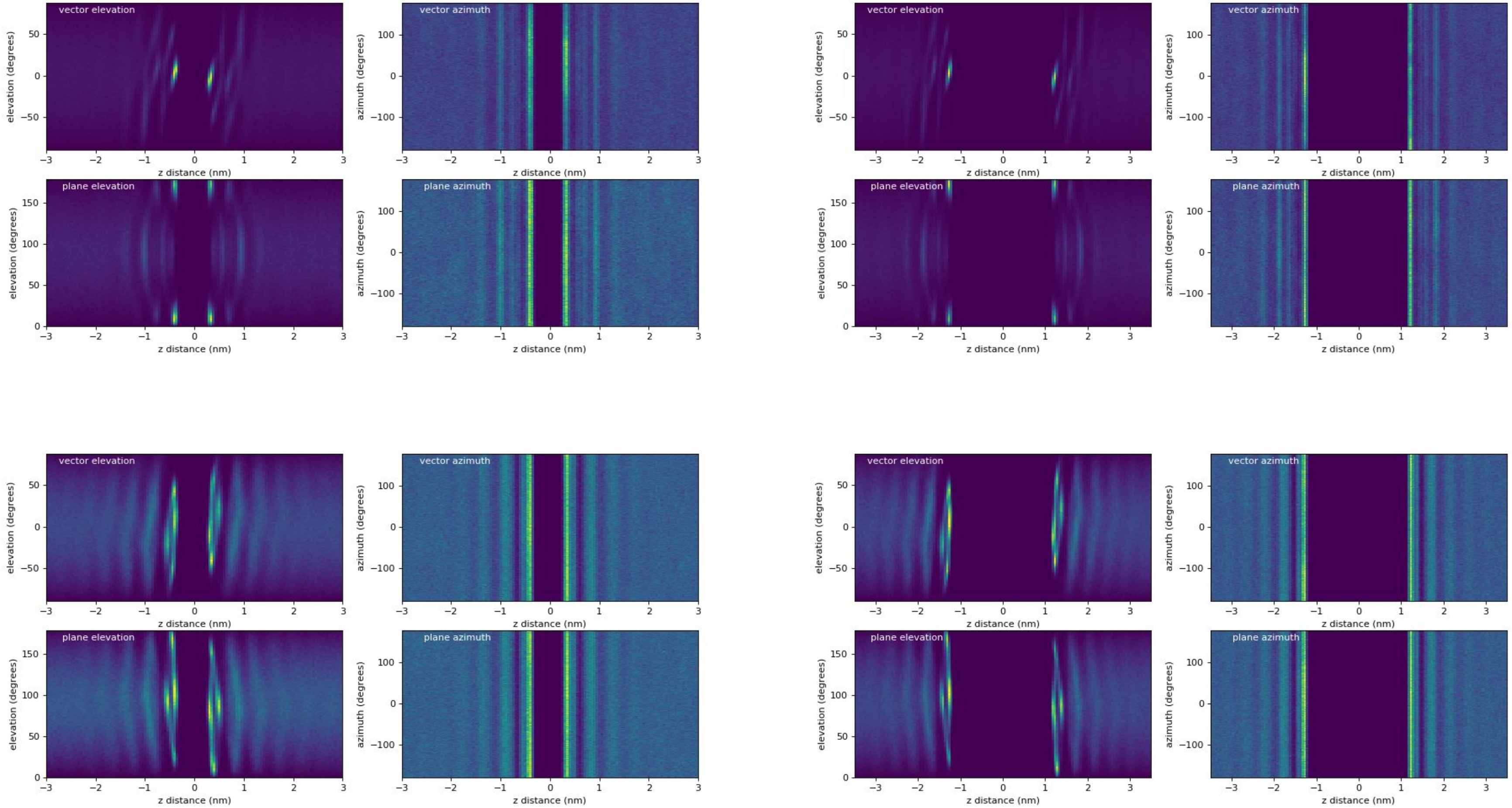

Figure S3.2: *contd.*

ETH

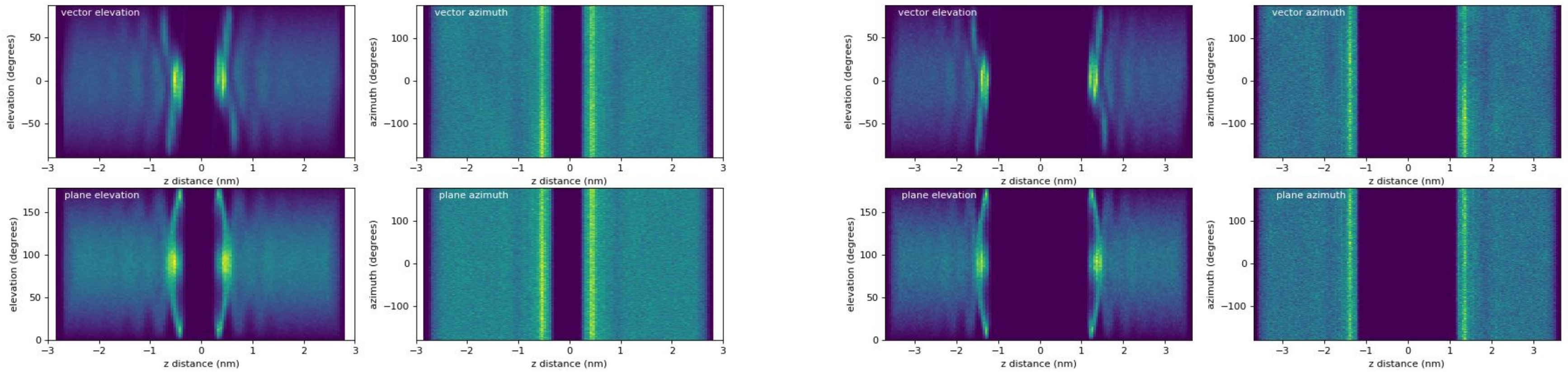

NMP

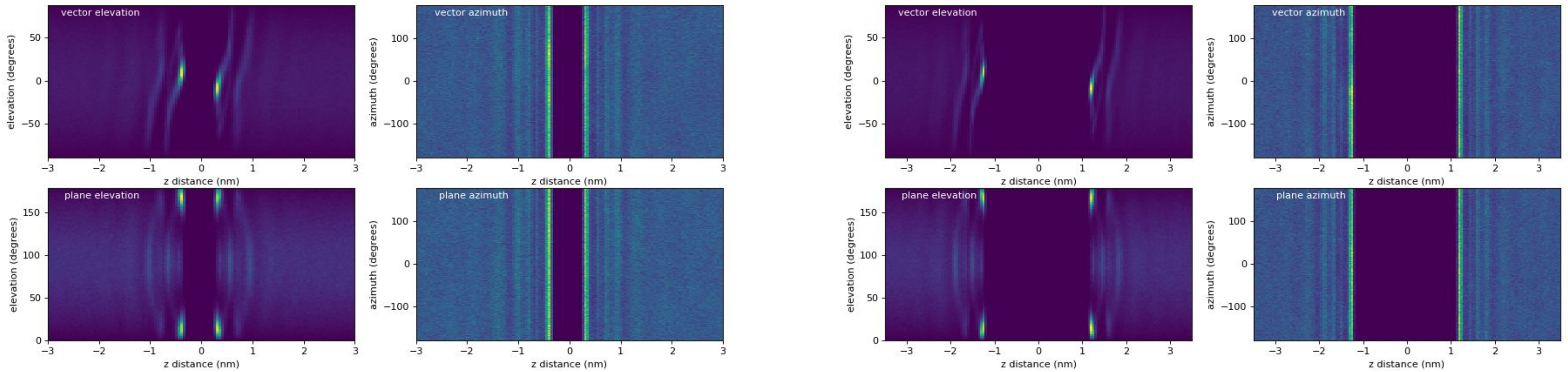

Figure S3.2: *contd.*

PRL

H$_2$O

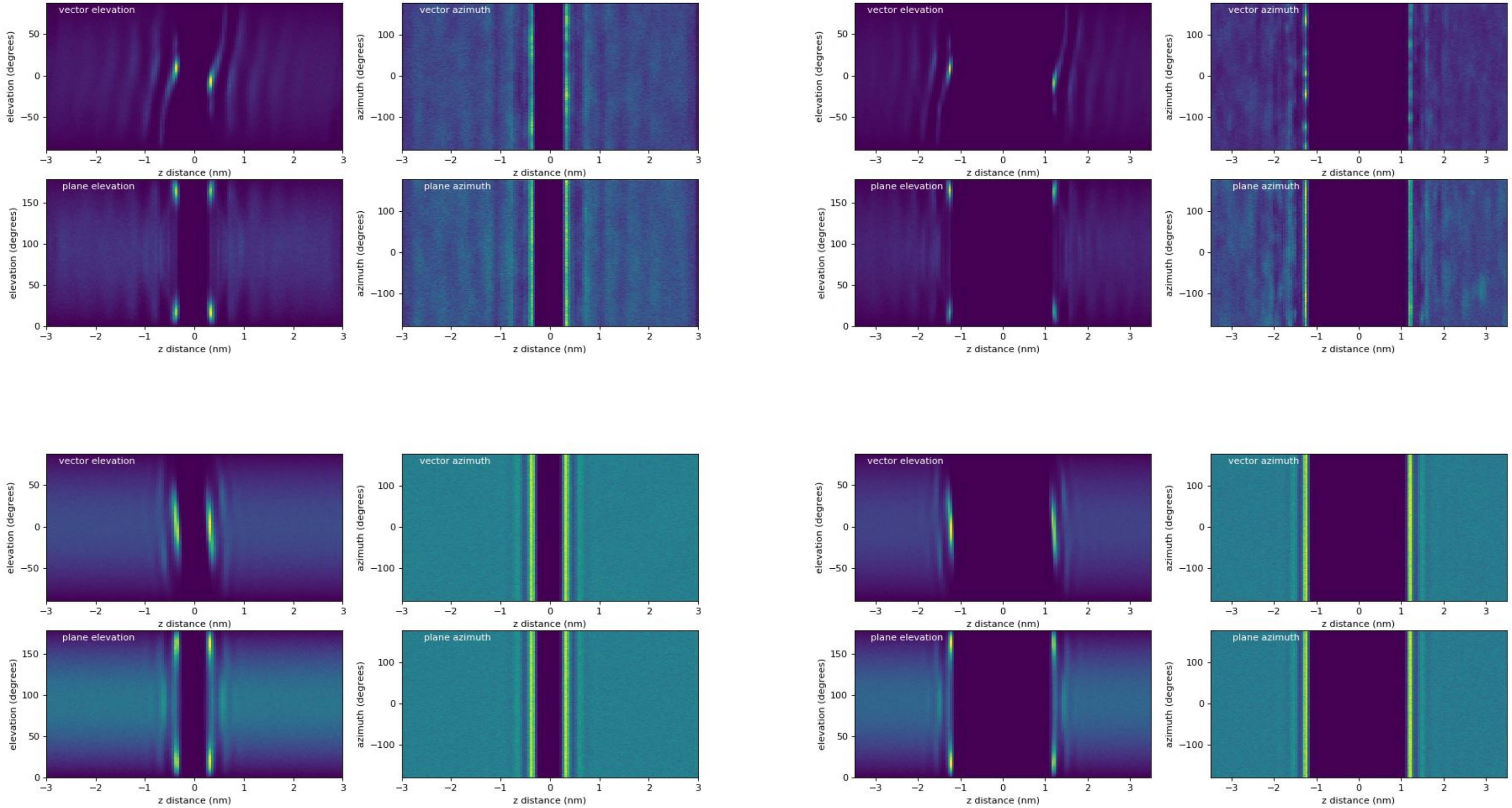

Figure S3.2: *contd.*

3.3 Clustering of the surface adsorbed molecules

Material	Graphene	Graphite
SOLVENT		
ACT		

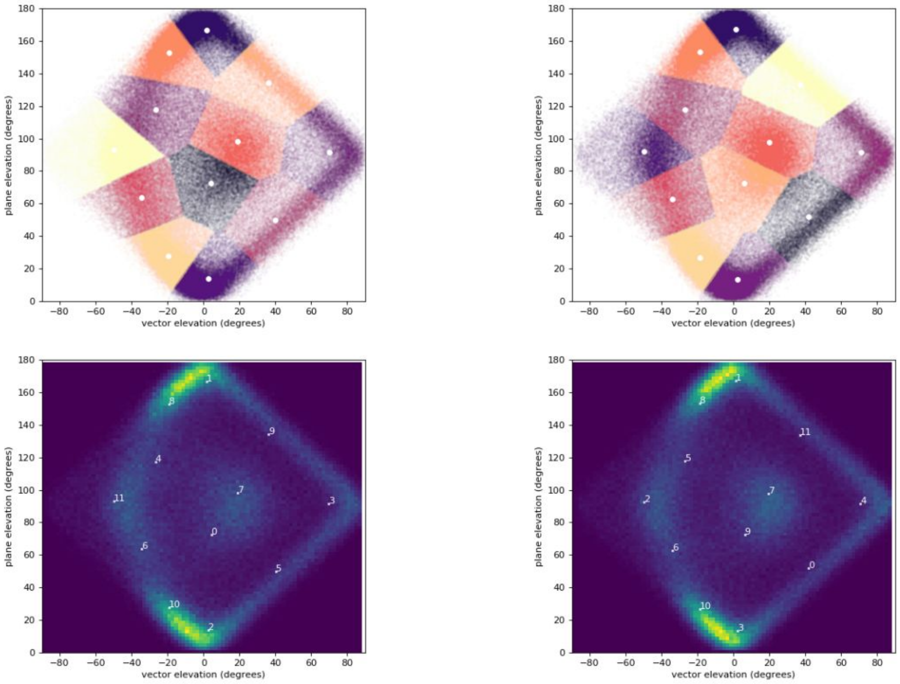

***Figure S3.3**: **Clustering solvents according to their orientation with respect of graphene** (left) **or graphite** (right**)**.*

***Top subfigures**: The vector and plane elevation of each solvent molecule are shown with a point on. Points colour determines which cluster they belong to. White points show cluster centres.*

***Bottom subfigures**: Elevation probability density. Solvents preferential alignments are revealed by lighter colours, whereby the narrower these regions, the more specific the binding. Clusters are marked with a dot and numbered. Cluster centres associated with the most likely alignment regions are selected for further analysis, see **Table S3.4**. Solvent alignments on graphene or graphite surfaces are comparable.*

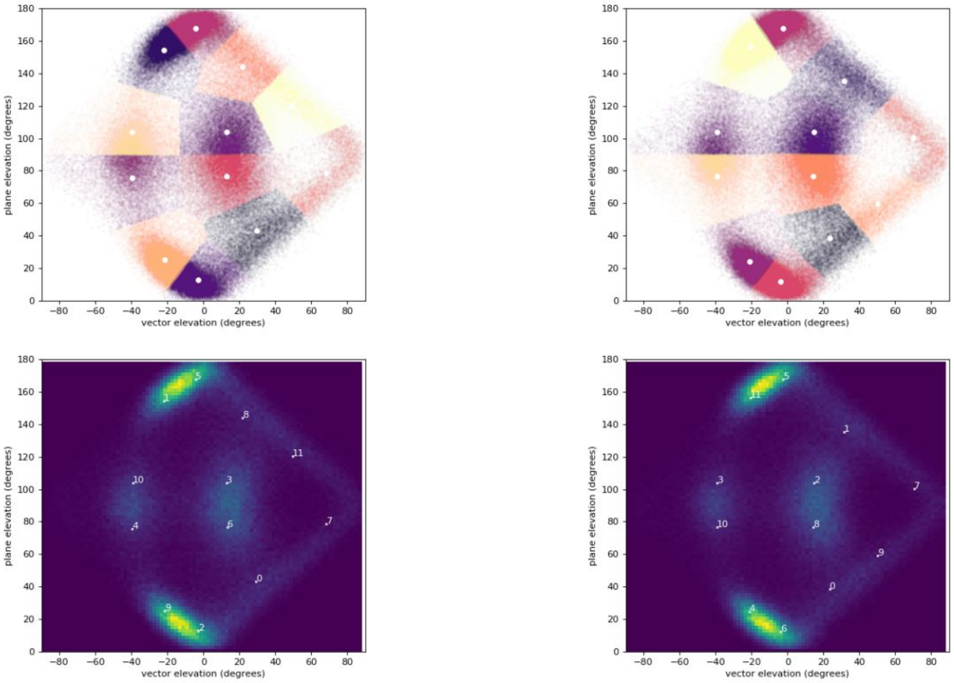

CPN

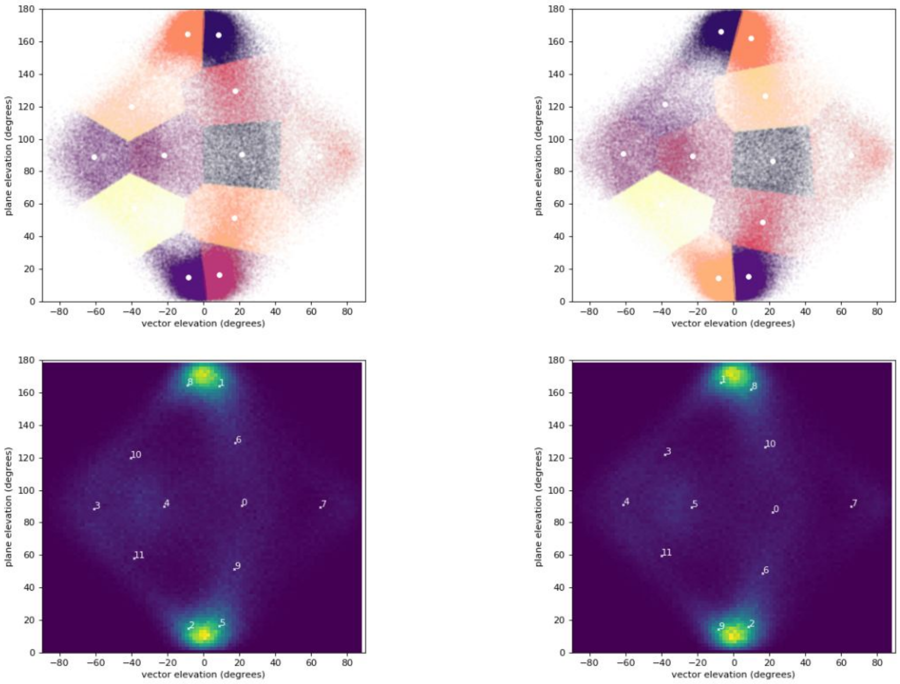

DMF

Figure S3.3: *contd.*

DMI

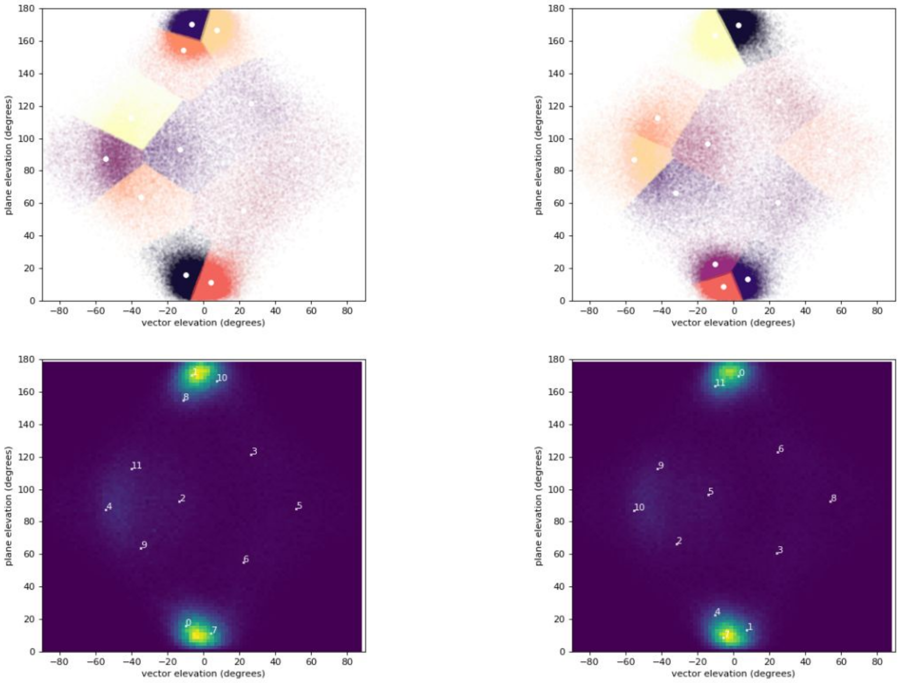

DMSO

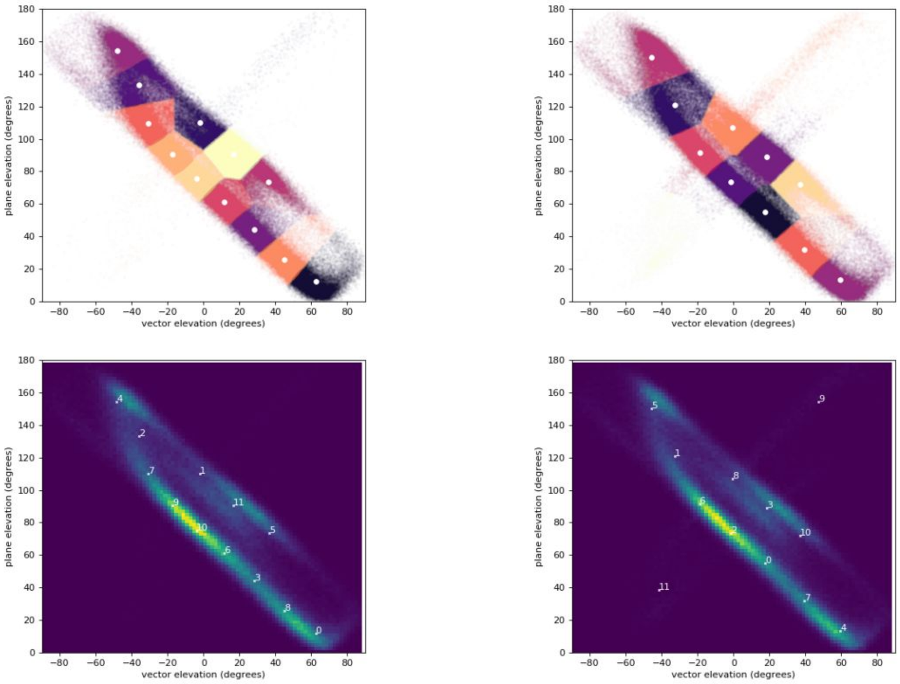

***Figure S3.3**: contd.*

ETH

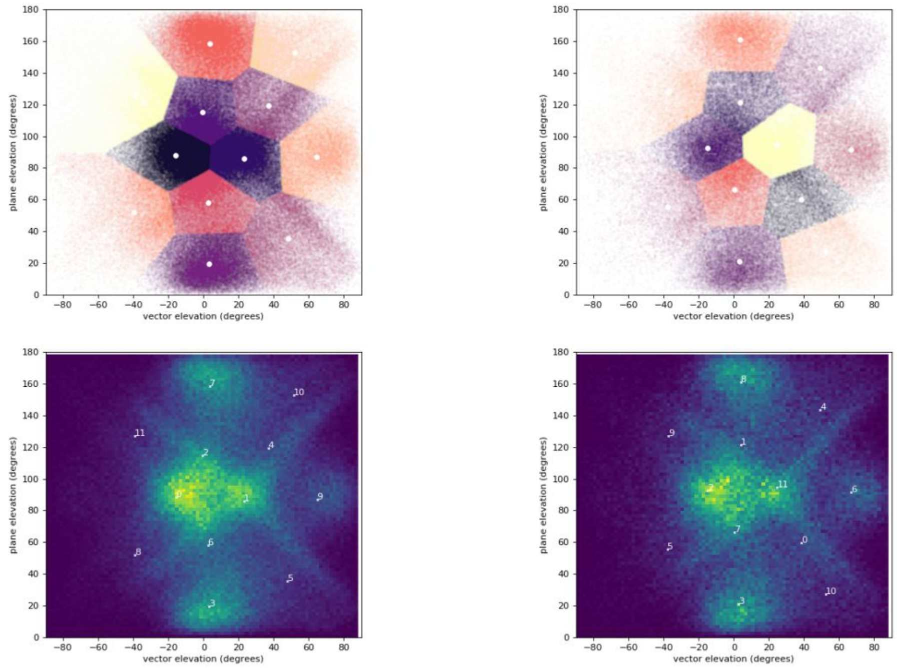

NMP

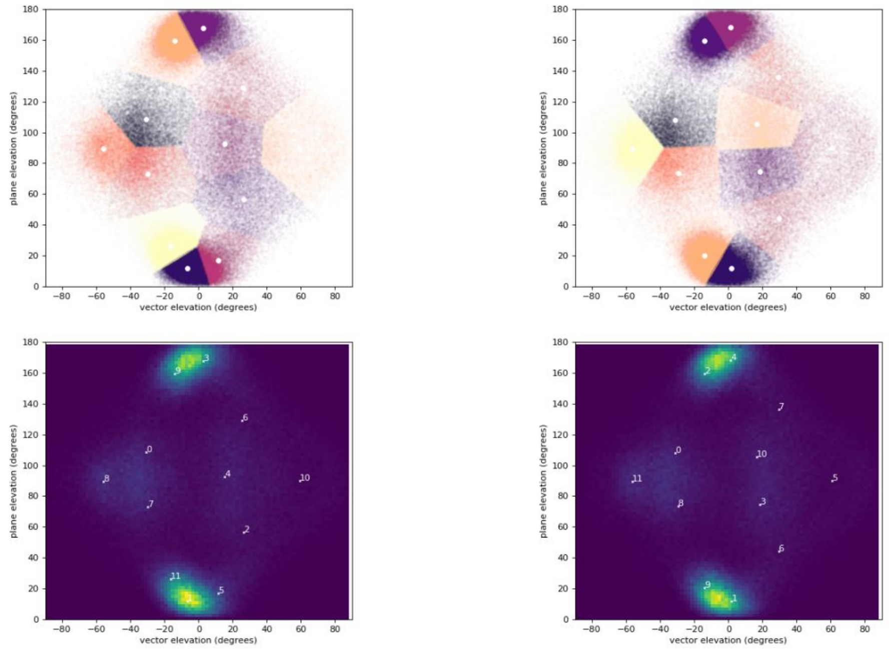

Figure S3.3*: contd.*

PRL

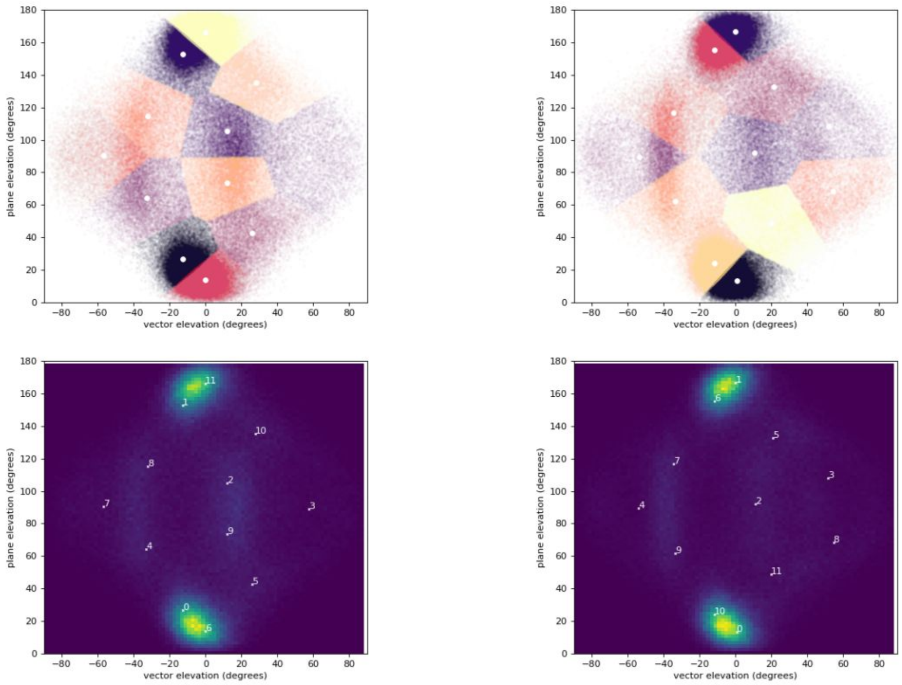

H_2O

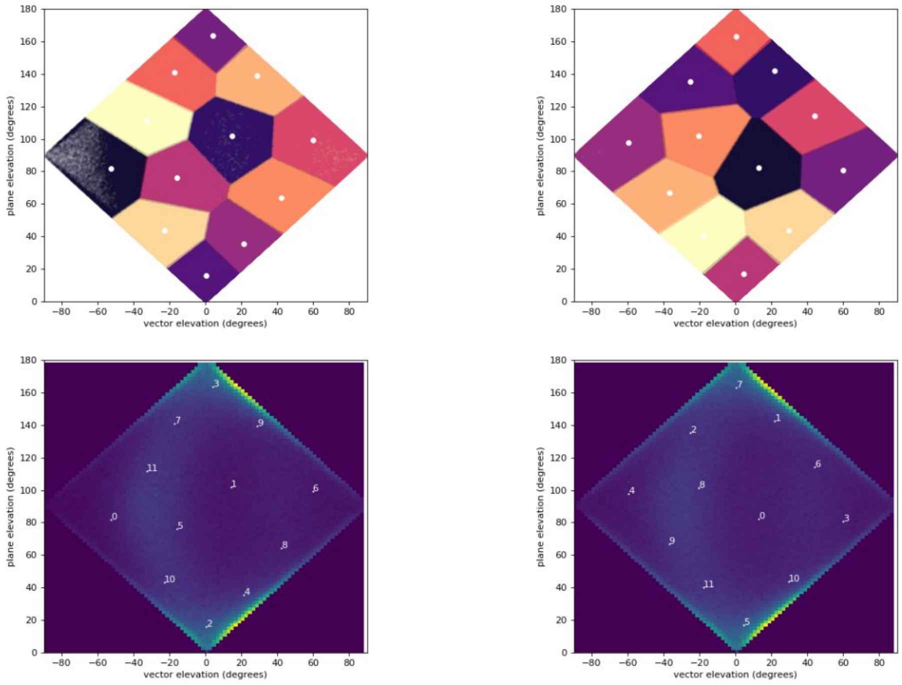

***Figure S3.3**: contd.*

3.4 Representative structures from MD from DFT

Table S3.4: *Cluster ID number that is chosen as representative structure for further DFT refinement, and their elevation angles. Cluster ID numbers are randomly assigned, and shown over* ***Figure S3.3****. Please note, that due to symmetrical nature of all solvent molecules, but DMSO, the elevation of X degrees is equivalent to the elevation of 180-X degrees.*

**Solvent**	**Graphene systems**			**Graphite Systems**		
	cluster ID no.	vector elev, deg	plane elev, deg	*cluster no.*	vector elev, deg	plane elev, deg
**ACT**	*1*	1.6	166.6	*3*	2.4	13.5
**CPN**	*2*	-2.8	12.9	*5*	-2.3	167.6
**DMF**	*2*	-8.5	14.8	*1*	-7.2	166.2
**DMI**	*7*	3.9	11.2	*7*	-5.5	8.6
**DMSO**	*10*	-3.9	75.3	*2*	-1.5	73.6
**ETH**	*0*	-15.7	88.2	*2*	-15.1	92.7
**NMP**	*1*	-6.5	11.8	*1*	1.9	11.9
**PRL**	*6*	-0.2	13.8	*0*	0.6	13.4
**H_2O**	*2*	0.3	16.0	*5*	4.5	17.2

4. Planewave density functional theory calculations

4.1 Test of solvation shell size

<table>
<tr><th rowspan="2">Solvent shell inclusion radius, Å</th><th colspan="2">DMF + Graphene</th><th colspan="2">ETH + Graphene</th></tr>
<tr><th>DMF</th><th>Graphene</th><th>ETH</th><th>Graphene</th></tr>
<tr><td>in vacuum</td><td>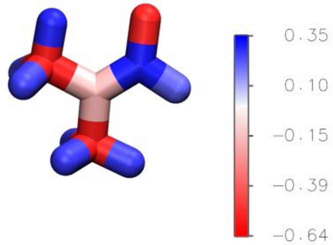</td><td>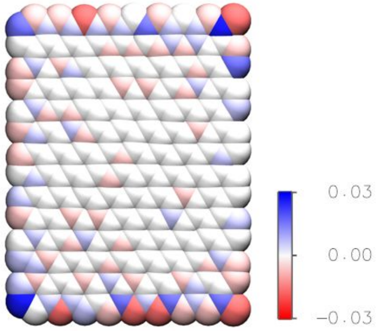</td><td>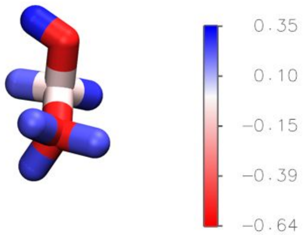</td><td>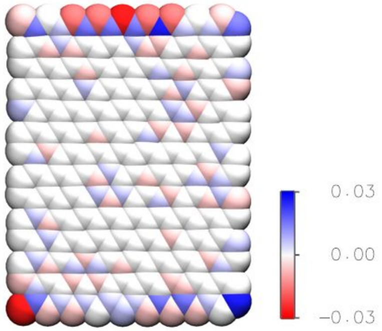</td></tr>
</table>

Figure S4.1: Mullikan charge distribution of the DMF (left column) and ETH (right column) solvents on the graphene slab*. The included shell of solvents with a radius from 3 to 8 Å around the central solvent molecule (shown in bold) over a slab of graphene (shown as grey spheres) under the central solvent molecule. The individual components are also calculated in vacuum and given. The red-white-blue colours represent Millikan system charges, colours are fixed between -0.64 (red) and +0.35 (blue) for all the solvents and between -0.03 and +0.03 for graphene layer. The charges for each atom, and the differences between models are given in the* ***Table 4.1****. For each system the charges are projected over the central solvent molecule (left sub-column) or over the graphene surface (right sub-column).*

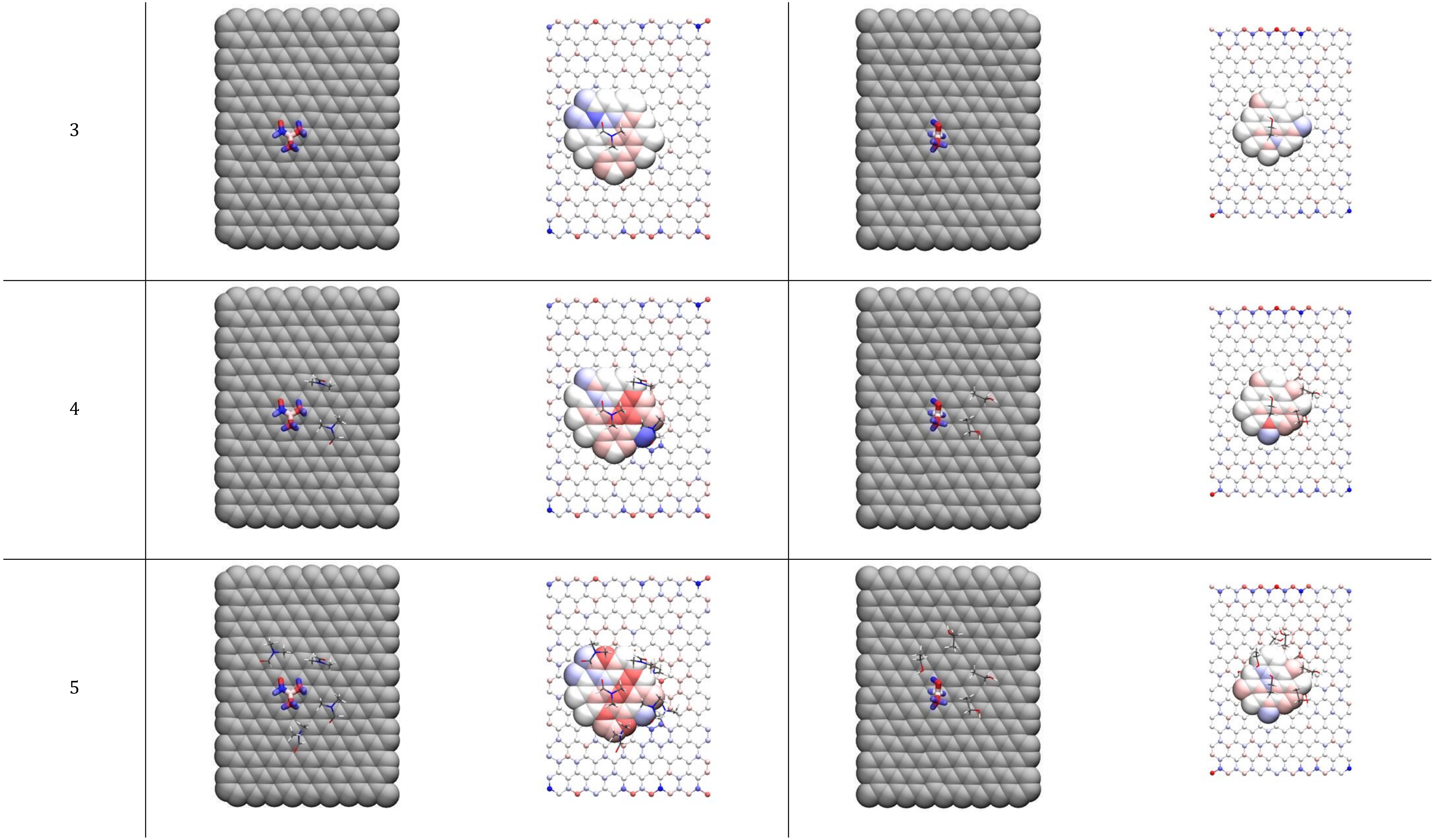

Figure S4.1: *contd.*

6	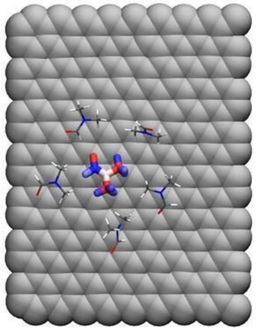	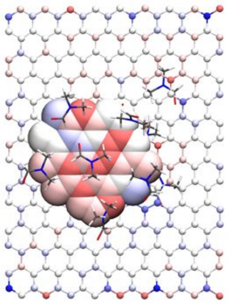	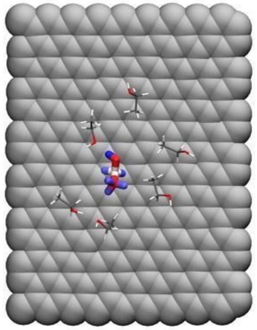	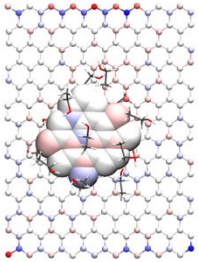
7	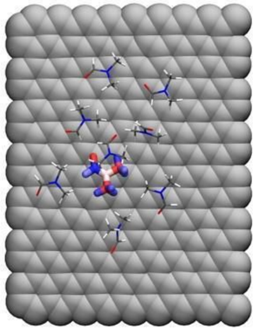	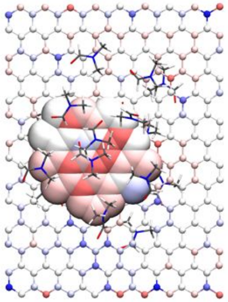	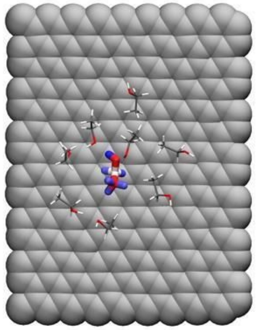	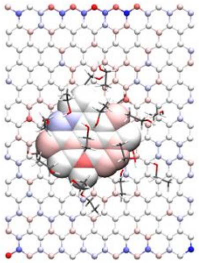
8	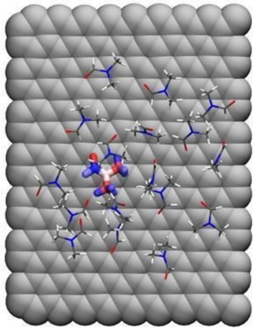	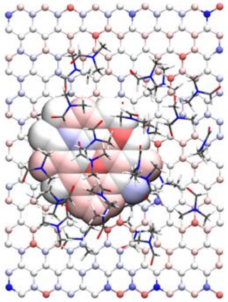	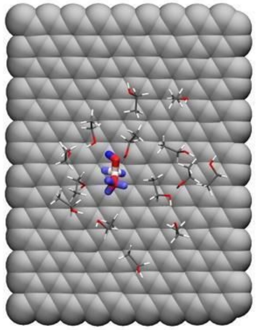	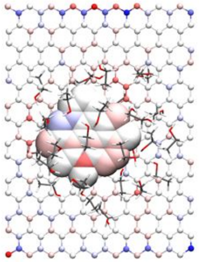

Figure S4.1: *contd.*

9

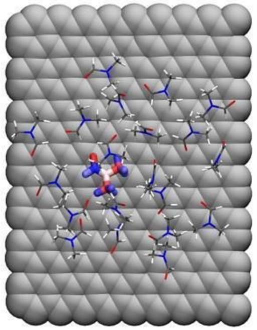

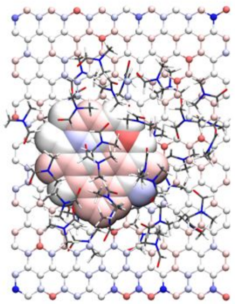

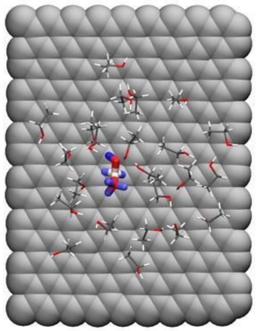

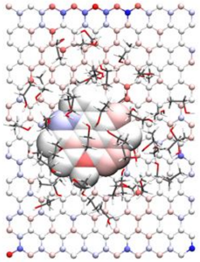

Figure S4.1: *contd.*

Table S4.1: *Summary of DFT charges. The corresponding visual representation is given in the* ***Figure S4.1****.*

DMF + GRA1

DMF	VAC	Delta(3-vac)	s3	Delta(4-3)	s4	Delta(5-4)	s5	Delta(6-5)	s6	Delta(7-6)	s7	Delta(8-7)	s8	Delta(9-8)	s9
C1	-0.66	0.02	-0.64	0.00	-0.64	0.04	-0.6	0.04	-0.64	0.05	-0.59	0.00	-0.59	0.00	-0.59
N1	-0.34	0.01	-0.33	0.00	-0.33	0.01	-0.32	0.01	-0.33	0.02	-0.31	0.00	-0.31	0.00	-0.31
C2	-0.64	0.00	-0.64	0.00	-0.64	0.03	-0.61	0.03	-0.64	0.04	-0.60	0.03	-0.57	0.00	-0.57
C3	0.35	0.00	0.35	0.00	0.35	0.00	0.35	0.00	0.35	0.03	0.38	0.00	0.38	0.00	0.38
O1	-0.62	0.00	-0.62	0.00	-0.62	0.00	-0.62	0.00	-0.62	0.00	-0.62	0.01	-0.63	0.01	-0.64
H1	0.33	0.00	0.33	0.00	0.33	0.02	0.31	0.02	0.33	0.02	0.31	0.00	0.31	0.01	0.3
H2	0.24	0.00	0.24	0.00	0.24	0.01	0.23	0.01	0.24	0.03	0.21	0.01	0.22	0.00	0.22
H3	0.28	0.00	0.28	0.00	0.28	0.02	0.26	0.02	0.28	0.01	0.27	0.01	0.26	0.00	0.26
H4	0.29	0.00	0.29	0.00	0.29	0.01	0.28	0.01	0.29	0.01	0.28	0.01	0.29	0.00	0.29
H5	0.29	0.00	0.29	0.00	0.29	0.02	0.27	0.02	0.29	0.02	0.27	0.02	0.25	0.00	0.25
H6	0.27	0.00	0.27	0.00	0.27	0.01	0.26	0.01	0.27	0.02	0.25	0.00	0.25	0.01	0.26
H7	0.21	0.00	0.21	0.00	0.21	0.01	0.22	0.01	0.21	0.03	0.18	0.00	0.18	0.01	0.19
total charge central mol	**0.00**		**0.03**		**0.03**		**0.03**		**0.03**		**0.03**		**0.04**		**0.04**
no. mol in system	**1**		**1**		**5**		**9**		**11**		**18**		**31**		**41**
total charge of all mol	**0.00**		**0.03**		**0.10**		**0.13**		**0.19**		**0.27**		**0.37**		**0.52**

GRA	VAC	Delta(3-vac)	s3	Delta(4-3)	s4	Delta(5-4)	s5	Delta(6-5)	s6	Delta(7-6)	s7	Delta(8-7)	s8	Delta(9-8)	s9
C3	0.00	0.00	0.00	0.02	0.02	0.00	0.01	0.00	0.01	0.00	0.01	0.00	0.01	0.00	0.01
C1	0.00	0.00	0.00	0.01	-0.01	0.00	-0.01	0.00	-0.01	0.00	-0.01	0.00	-0.01	0.00	-0.01
C2	0.00	0.00	0.00	0.01	-0.01	0.00	-0.01	0.00	-0.01	0.00	-0.01	0.01	0.00	0.00	0.00
C3	0.00	0.00	0.00	0.01	-0.01	0.00	0.00	0.00	0.00	0.00	0.00	0.00	0.00	0.00	0.00
C2	0.00	0.01	-0.01	0.00	-0.01	0.00	-0.02	0.00	-0.02	0.01	-0.01	0.00	-0.01	0.00	-0.01
C3	0.00	0.01	-0.01	0.00	-0.01	0.00	-0.01	0.00	-0.01	0.00	-0.01	0.00	-0.01	0.00	-0.01
C4	0.00	0.01	-0.01	0.01	0.00	0.00	0.00	0.00	0.00	0.00	0.00	0.00	0.00	0.00	0.00
C1	0.00	0.01	-0.01	0.01	-0.02	0.00	-0.01	0.00	-0.01	0.00	-0.01	0.00	-0.01	0.00	-0.01
C2	0.00	0.01	-0.01	0.01	-0.02	0.00	-0.02	0.00	-0.02	0.00	-0.02	0.01	-0.01	0.00	-0.01
C3	-0.01	0.00	-0.01	0.01	-0.02	0.00	-0.02	0.00	-0.02	0.00	-0.02	0.01	-0.01	0.00	-0.01
C4	0.00	0.01	-0.01	0.01	-0.02	0.00	-0.02	0.00	-0.02	0.00	-0.02	0.00	-0.02	0.00	-0.02
C1	0.00	0.00	0.00	0.00	0.00	0.00	0.00	0.00	0.00	0.00	0.00	0.00	0.00	0.00	0.00
C2	0.00	0.00	0.00	0.00	0.00	0.00	0.00	0.00	0.00	0.00	0.00	0.00	0.00	0.00	0.00
C1	0.00	0.00	0.00	0.00	0.00	0.00	-0.01	0.00	-0.01	0.00	-0.01	0.00	-0.01	0.00	-0.01
C2	-0.01	0.00	-0.01	0.00	-0.01	0.00	-0.01	0.00	-0.01	0.00	-0.01	0.00	-0.01	0.00	-0.01
C3	0.00	0.01	-0.01	0.00	-0.01	0.00	-0.02	0.00	-0.02	0.00	-0.02	0.01	-0.01	0.00	-0.01
C4	0.00	0.00	0.00	0.01	-0.01	0.00	-0.01	0.00	-0.01	0.00	-0.01	0.00	-0.01	0.00	-0.01
C1	-0.01	0.00	-0.01	0.01	-0.02	0.00	-0.02	0.00	-0.02	0.00	-0.02	0.00	-0.02	0.01	-0.01
C2	0.00	0.00	0.00	0.01	-0.01	0.00	-0.01	0.00	-0.01	0.00	-0.01	0.01	0.00	0.00	0.00
C3	0.00	0.01	0.01	0.00	0.01	0.00	0.01	0.00	0.01	0.00	0.01	0.00	0.01	0.00	0.01
C4	0.00	0.01	0.01	0.01	0.00	0.00	0.00	0.00	0.00	0.00	0.00	0.00	0.00	0.00	0.00
C1	0.00	0.00	0.00	0.00	0.00	0.00	-0.01	0.00	-0.01	0.00	-0.01	0.00	-0.01	0.00	-0.01
C2	0.00	0.00	0.00	0.00	0.00	0.00	-0.02	0.00	-0.02	0.01	-0.01	0.00	-0.01	0.00	-0.01
C3	0.00	0.00	0.00	0.00	0.00	0.01	0.00	0.01	-0.01	0.00	-0.01	0.01	0.00	0.00	0.00
C4	0.00	0.00	0.00	0.00	0.00	0.01	0.00	0.01	-0.01	0.00	-0.01	0.00	-0.01	0.00	-0.01
C1	0.00	0.00	0.00	0.01	-0.01	0.01	-0.01	0.01	-0.02	0.00	-0.02	0.01	-0.01	0.00	-0.01
C2	0.00	0.00	0.00	0.01	-0.01	0.01	0.00	0.01	-0.01	0.00	-0.01	0.00	-0.01	0.00	-0.01
C3	0.00	0.01	0.01	0.01	0.00	0.00	0.00	0.00	0.00	0.00	0.00	0.00	0.00	0.00	0.00
C4	0.00	0.02	0.02	0.01	0.01	0.00	0.01	0.00	0.01	0.01	0.00	0.01	0.01	0.00	0.01
C1	-0.01	0.01	0.00	0.01	-0.01	0.00	-0.01	0.00	-0.01	0.01	-0.02	0.01	-0.01	0.00	-0.01
C2	0.01	0.00	0.01	0.00	0.01	0.00	0.01	0.00	0.01	0.01	0.00	0.00	0.00	0.00	0.00
C1	0.00	0.00	0.00	0.00	0.00	0.01	0.00	0.01	-0.01	0.00	-0.01	0.00	-0.01	0.00	-0.01
C4	0.00	0.01	0.01	0.01	0.00	0.01	0.01	0.01	0.00	0.00	0.00	0.00	0.00	0.00	0.00
total charge of fragment	**-0.03**		**-0.03**		**-0.17**		**-0.20**		**-0.26**		**-0.27**		**-0.18**		**-0.17**
total charge of graohene	**0.00**		**-0.06**		**-0.10**		**-0.14**		**-0.17**		**-0.26**		**-0.41**		**-0.57**

ETH + GRA1

ETH	vac	Delta(3-vac)	s3	Delta(4-3)	s4	Delta(5-4)	s5	Delta(6-5)	s6	Delta(7-6)	s7	Delta(8-7)	s8	Delta(9-8)	s9
C1	-0.27	0.03	-0.24	0.01	-0.23	0.00	-0.23	0.00	-0.23	0.00	-0.23	0.00	-0.23	0.00	-0.23
C2	-0.77	0.00	-0.77	0.01	-0.76	0.01	-0.75	0.01	-0.74	0.01	-0.75	0.00	-0.75	0.00	-0.75
O	-0.81	0.00	-0.81	0.00	-0.81	0.01	-0.80	0.00	-0.80	0.01	-0.79	0.00	-0.79	0.00	-0.79
H1	0.3	0.01	0.29	0.02	0.27	0.01	0.26	0.00	0.26	0.01	0.27	0.01	0.26	0.00	0.26
H2	0.22	0.01	0.21	0.01	0.22	0.01	0.21	0.00	0.21	0.01	0.22	0.00	0.21	0.01	0.22
H3	0.25	0.00	0.25	0.00	0.25	0.00	0.25	0.00	0.25	0.00	0.25	0.01	0.27	0.01	0.26
H4	0.27	0.00	0.27	0.00	0.27	0.00	0.27	0.02	0.25	0.01	0.26	0.00	0.26	0.00	0.26
H5	0.25	0.00	0.25	0.01	0.24	0.00	0.24	0.01	0.23	0.01	0.24	0.01	0.23	0.00	0.23
H6	0.55	0.00	0.55	0.00	0.55	0.02	0.53	0.00	0.53	0.01	0.54	0.00	0.54	0.00	0.54
**total charge central mol**	**-0.01**		**0.00**		**0.00**		**-0.02**		**-0.04**		**0.01**		**0.00**		**0.00**
**no. mol in system**	**1**		**1**		**6**		**11**		**15**		**21**		**32**		**50**
**total charge of all mol**	**-0.01**		**0.00**		**0.08**		**0.15**		**0.16**		**0.25**		**0.23**		**0.36**

GRA	vac	Delta(3-vac)	s3	Delta(4-3)	s4	Delta(5-4)	s5	Delta(6-5)	s6	Delta(7-6)	s7	Delta(8-7)	s8	Delta(9-8)	s9
C3	0.00	0.00	0.00	0.00	0.00	0.00	0.00	0.00	0.00	0.00	0.00	0.00	0.00	0.00	0.00
C1	0.00	0.00	0.00	0.00	0.00	0.01	-0.01	0.00	-0.01	0.00	-0.01	0.01	-0.01	0.01	0.00
C2	0.00	0.00	0.00	0.00	0.00	0.00	0.00	0.00	0.00	0.00	0.00	0.00	0.00	0.00	0.00
C3	0.00	0.00	0.00	0.00	0.00	0.00	0.00	0.00	0.00	0.01	0.01	0.00	0.01	0.01	0.01
C2	0.00	0.00	0.00	0.01	0.01	0.00	0.01	0.00	0.01	0.01	0.00	0.00	0.00	0.00	0.00
C3	-0.01	0.00	-0.01	0.01	-0.02	0.01	-0.01	0.00	-0.01	0.01	-0.02	0.00	-0.02	0.02	-0.02
C4	0.01	0.01	0.00	0.00	0.00	0.00	0.00	0.00	0.00	0.00	0.00	0.00	0.00	0.00	0.00
C1	-0.01	0.00	-0.01	0.00	-0.01	0.00	-0.01	0.00	-0.01	0.00	-0.01	0.00	-0.01	0.01	-0.01
C2	0.01	0.01	0.00	0.00	0.00	0.01	0.01	0.00	0.01	0.01	0.00	0.00	0.00	0.00	0.00
C3	0.00	0.00	0.00	0.00	0.00	0.01	0.01	0.01	0.00	0.00	0.00	0.00	0.00	0.00	0.00
C4	0.00	0.00	0.00	0.00	0.00	0.01	0.01	0.00	0.01	0.00	0.01	0.00	0.01	0.01	0.01
C1	-0.01	0.00	-0.01	0.00	-0.01	0.01	0.00	0.00	0.00	0.00	0.00	0.00	0.00	0.00	0.00
C2	0.00	0.00	0.00	0.00	0.00	0.00	0.00	0.00	0.00	0.00	0.00	0.00	0.00	0.00	0.00
C3	0.00	0.00	0.00	0.01	-0.01	0.00	-0.01	0.01	0.00	0.01	-0.01	0.00	-0.01	0.01	-0.01
C4	0.01	0.00	0.01	0.01	0.00	0.00	0.00	0.00	0.00	0.00	0.00	0.00	0.00	0.00	0.00
C1	0.00	0.01	-0.01	0.00	-0.01	0.00	-0.01	0.00	-0.01	0.00	-0.01	0.00	-0.01	0.01	-0.01
C2	0.00	0.00	0.00	0.00	0.00	0.00	0.00	0.00	0.00	0.00	0.00	0.00	0.00	0.00	0.00
C3	0.00	0.00	0.00	0.00	0.00	0.00	0.00	0.00	0.00	0.00	0.00	0.00	0.00	0.00	0.00
C4	0.00	0.00	0.00	0.00	0.00	0.00	0.00	0.00	0.00	0.00	0.00	0.00	0.00	0.00	0.00
C1	0.00	0.00	0.00	0.00	0.00	0.00	0.00	0.00	0.00	0.00	0.00	0.00	0.00	0.00	0.00
C4	0.00	0.00	0.00	0.01	-0.01	0.00	-0.01	0.01	0.00	0.01	-0.01	0.00	-0.01	0.01	-0.01
C1	-0.01	0.00	-0.01	0.00	-0.01	0.00	-0.01	0.00	-0.01	0.00	-0.01	0.00	-0.01	0.01	-0.01
C2	0.00	0.01	0.01	0.01	0.00	0.00	0.00	0.00	0.00	0.00	0.00	0.00	0.00	0.00	0.00
C4	0.00	0.00	0.00	0.01	-0.01	0.00	-0.01	0.00	-0.01	0.00	-0.01	0.00	-0.01	0.01	-0.01
**total charge of fragment**	**-0.01**		**-0.03**		**-0.08**		**-0.04**		**-0.03**		**-0.07**		**-0.07**		**-0.06**
**total charge of graohene**	**0.05**		**0.04**		**-0.02**		**-0.09**		**-0.14**		**-0.21**		**-0.39**		**-0.56**

Table S4.1: *contd.*

4.2 Layer undulation and interlayer spacing from DFT

When compared to the results from the MD simulation, **Figure S2.5**, smaller sub-Angstrom fluctuations and narrower d-spacing are observed for DFT calculations compared to MD calculations. This is to be expected, as no temperature effect is included in the DFT. Some solvent-specific effect is observed, nevertheless still within the statistical uncertainty.

Layer undulation for graphite in each solvent

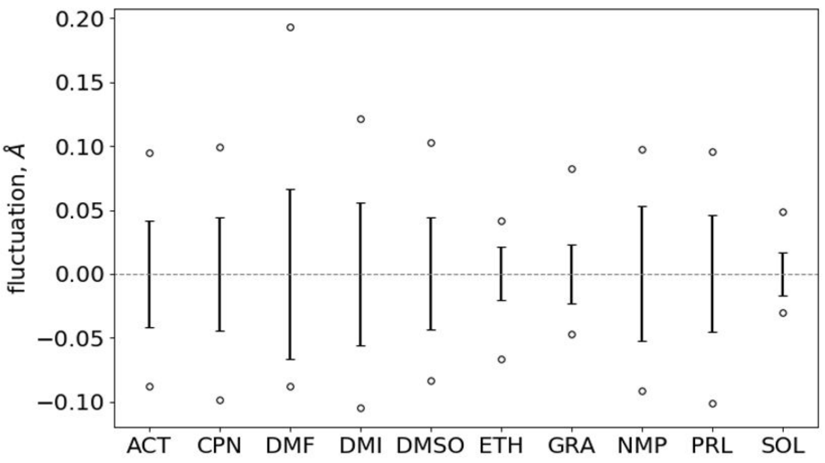

Interlayer spacing for graphite in each solvent

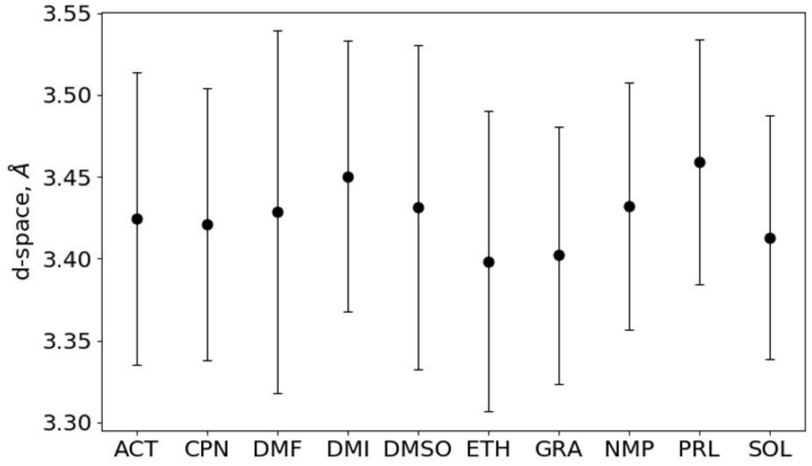

***Figure S4.2: Layer undulations and interlayer spacing of graphite in the presence of solvents**. Bars represent standard deviation of the positions of all atoms aggregated. Open circles represent lowest and highest atom positions observed.*

4.3 Density of States

Graphene **Graphite** **Pure solvents**

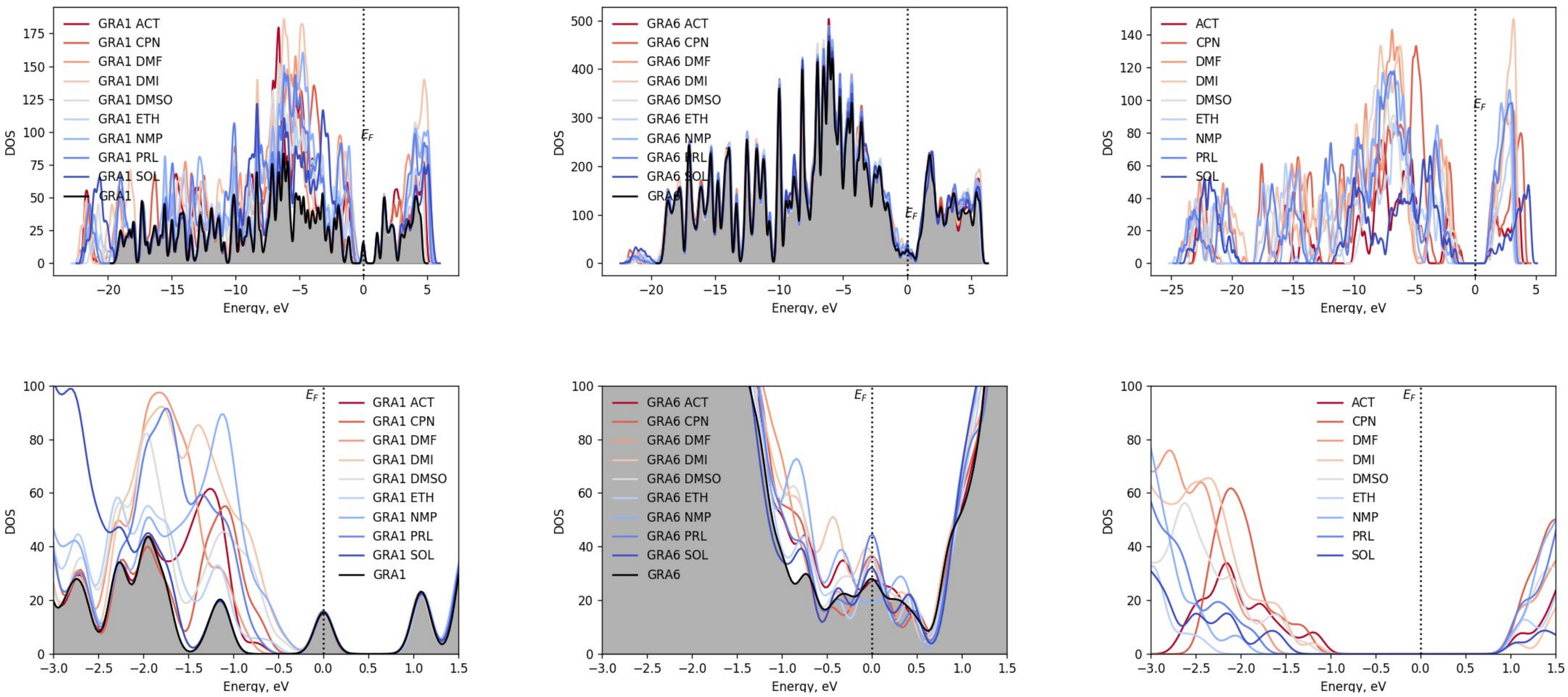

Figure S4.3: Analysis of Band Structure and DOS of graphene, graphite and pure solvent system. The Graphene and graphite contributions are shown in filled grey (electron/eV) for the systems. The top is complete DOS, the bottom is the zoom onto the Fermi area

***Figure S4.4**: DFT results - location of the partially occupied bands within the region (isovalue = 0.004).*

System	*Top view*	*Side view*
Graphite only	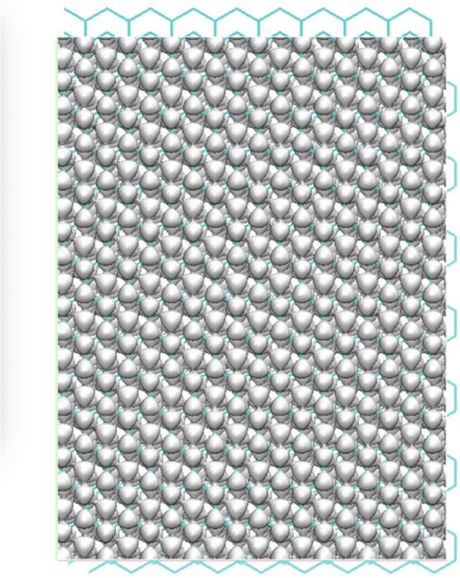	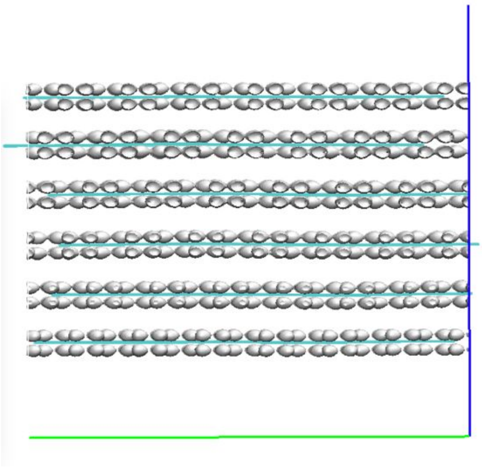
ACT	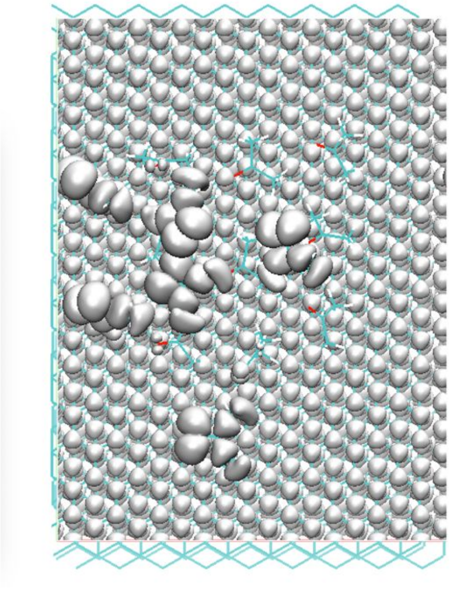	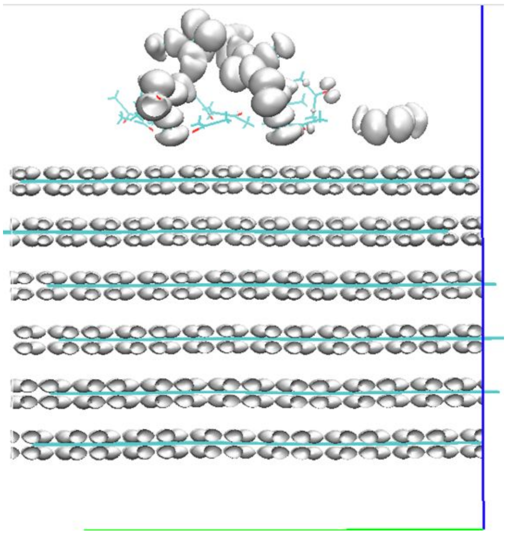

CPN

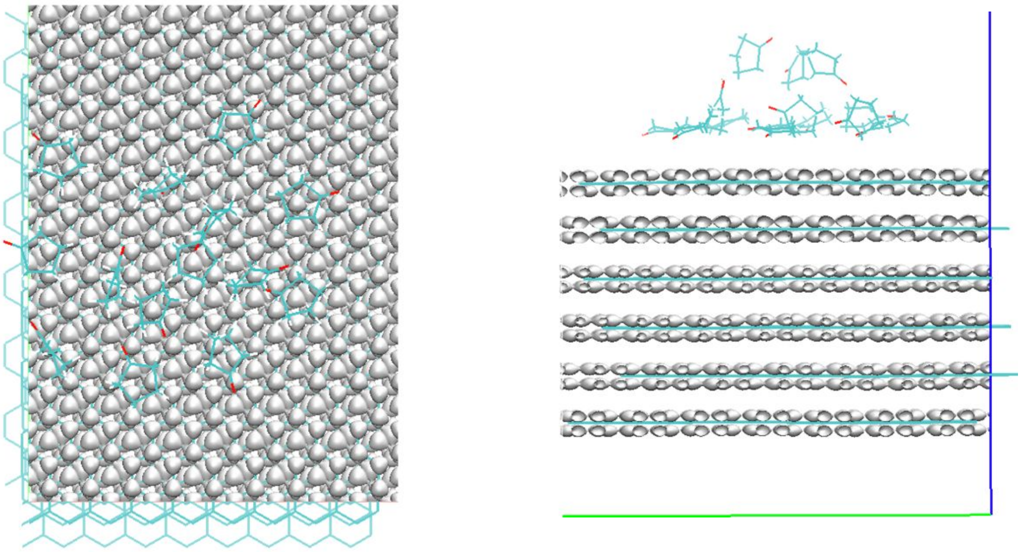

DMF

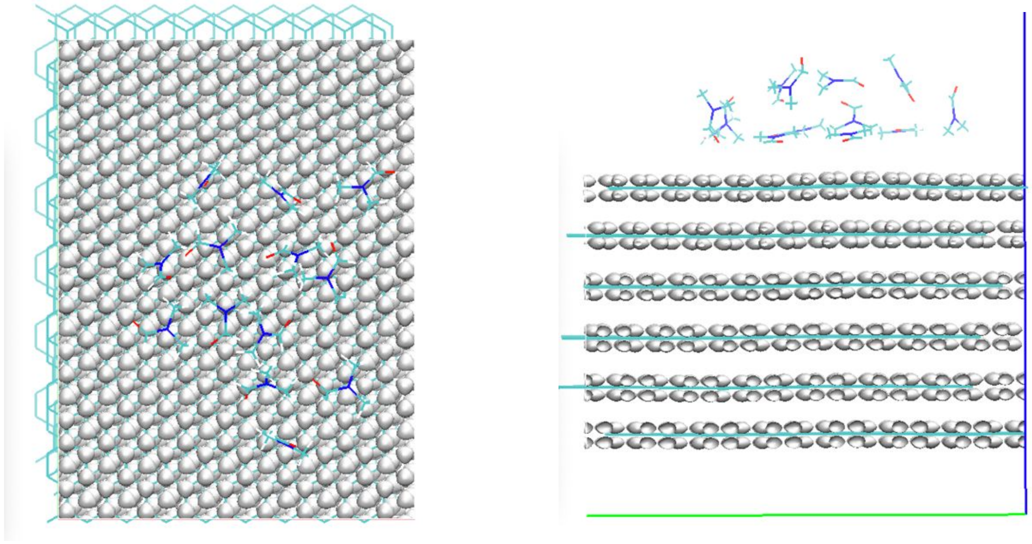

DMI

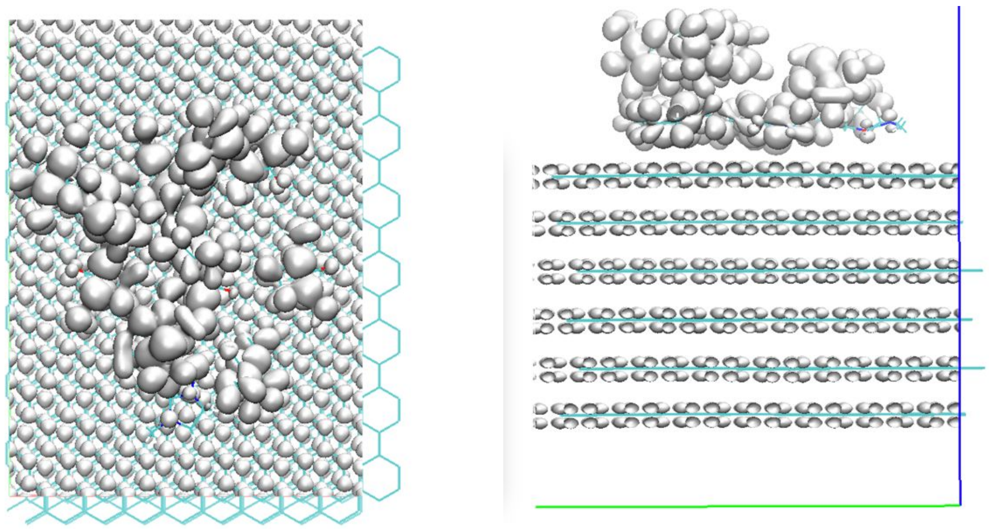

DMSO

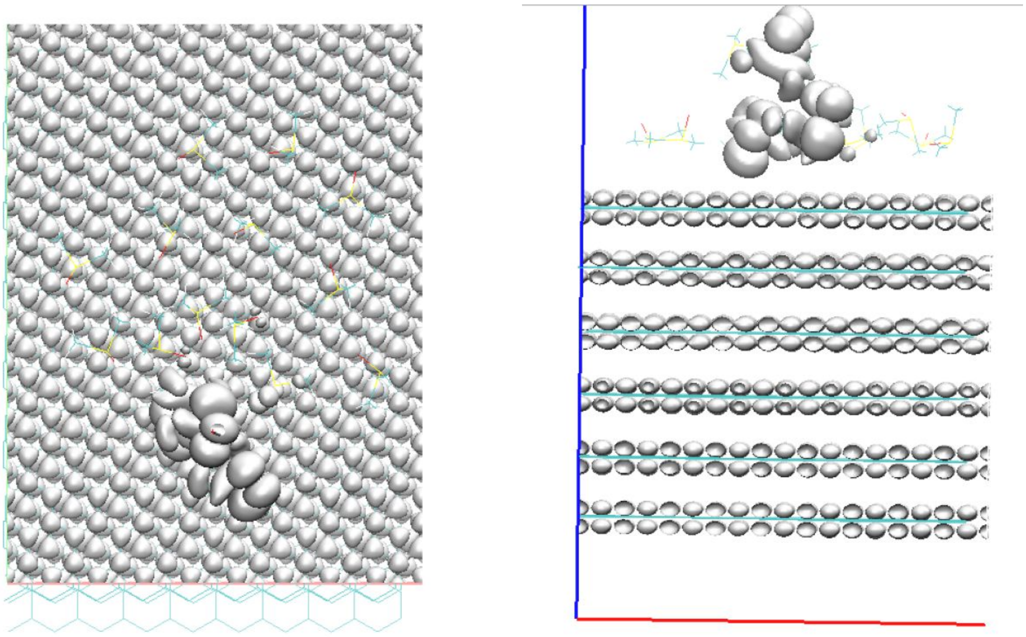

ETH

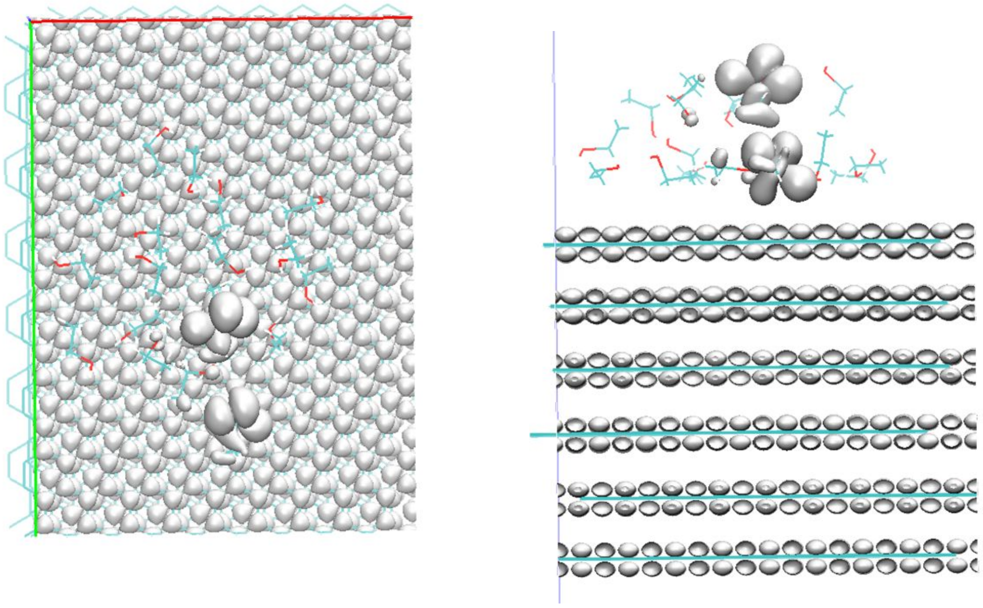

NMP

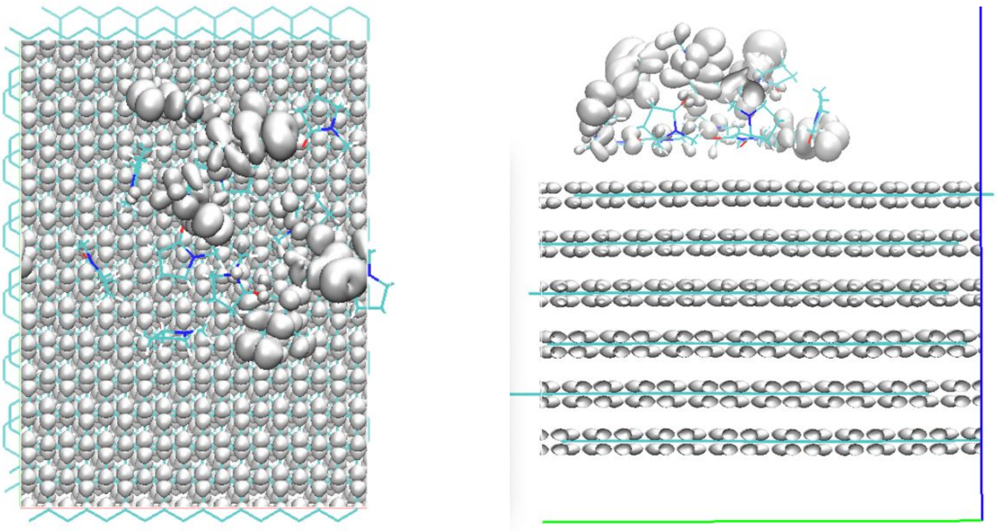

PRL

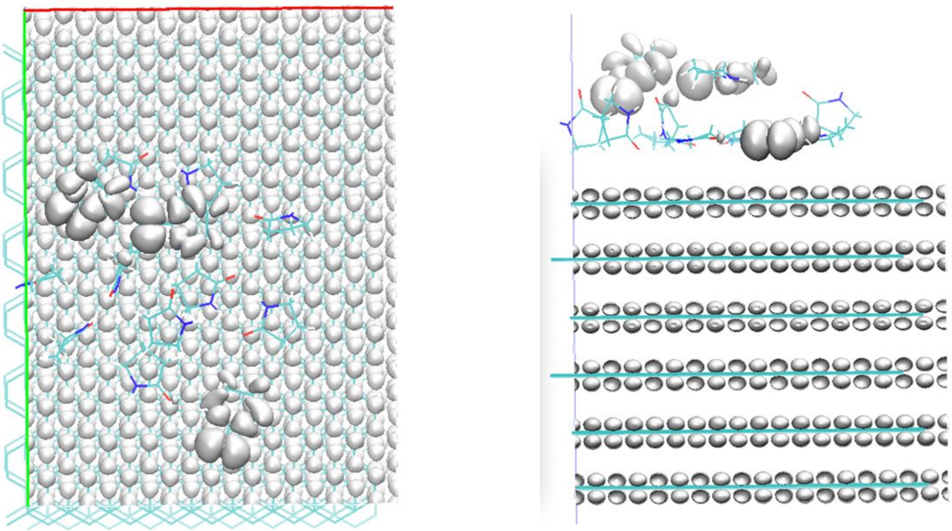

H_2O

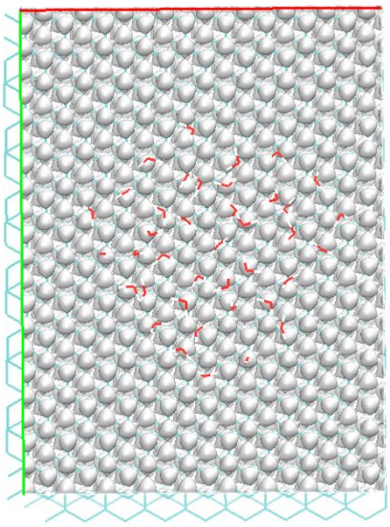

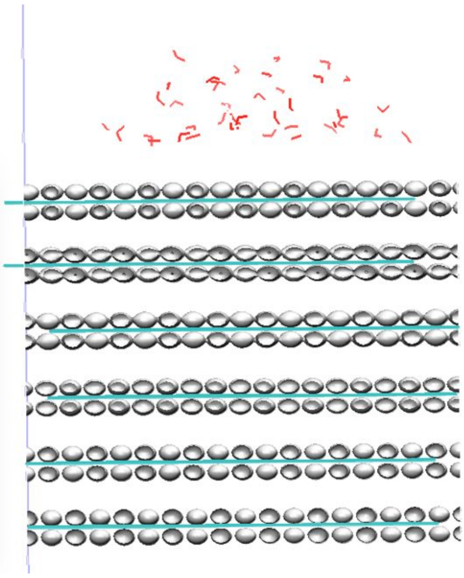

5 Experimental

5.1 Sample preparation

To gain insight on how well the modelling can predict laboratory performance, we experimentally tested the exfoliation capability of solvents (DMI, CPN, PRL) and compared them with NMP and DMF. 325 Mesh natural flake graphite was shear mixed in the pure solvent, followed by a single centrifugation process to remove large unexfoliated material. The dispersions were analysed with UV-vis-nIR and solid power dried onto microfilters for statistical Raman analysis; microscopy images were then taken from stable graphene dispersions. Optical absorbance was used to estimate the concentration of graphene flakes in a dispersion.

5.2 Raman

Raman spectra were recorded with a Horiba LabRam Evolution using a 532 nm, 1 mW laser and a x50 long working distance objective lens. The instrument was calibrated against the 520.7 cm^{-1} Raman signal of silicon. Dispersions were filtered through a 0.45 μm PTFE filter membrane to produce a smooth surface from which Raman spectra were collected directly. Over a hundred points were collected from each sample and a statistical workflow was used to established this was sufficient; these spectra were fitted with a six-order polynomial background and Lorentzian line shapes; further details published previously.[28]

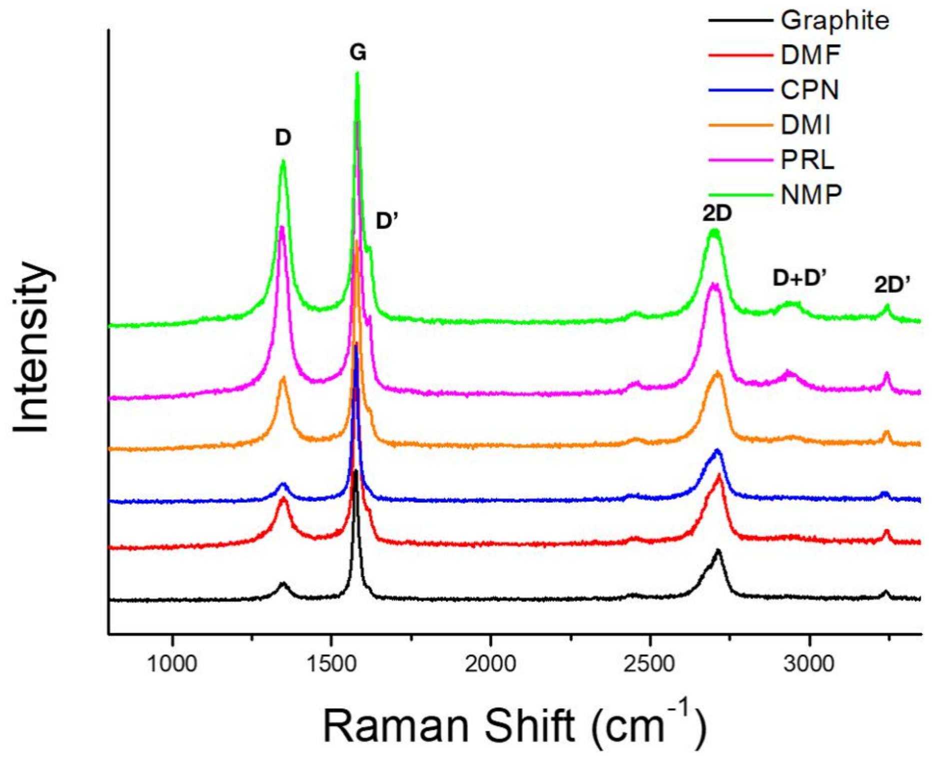

Figure S5.1: Representative Raman spectra of the material exfoliated with each solvent, *spectra selected from modal metrics for each sample.*

Raman analysis was used to measure exfoliated graphene within a bulk powder; here an automated stage collected hundreds of spectra from a regular grid of points over the sample surface. Regardless of the ordered grid, the sample is randomly distributed after shear mixing. These large data sets were then analysed by fitting each spectrum individually to extract common Raman metrics: I_D/I_G and I_{2D}/I_G. The presence of exfoliated few-layer graphene will increase the measured I_{2D}/I_G, and significant reductions in flake size or defects in the graphene sheet can be detected by an increased I_D/I_G.

The CPN exfoliated dispersion was unstable and the distribution of Raman metrics were unchanged from the original graphite; we therefore conclude CPN is a poor solvent for the exfoliation of graphene. DMF also shows little change to the starting graphite, the I_D/I_G ratio was observed to increase slightly as seen in the representative spectra and the slight rightward shift in the 3D bivariate histograms. I_{2D}/I_G also showed a slight increase; suggesting poor exfoliation of only a few flakes whilst the majority of the material was formed of unexfoliated graphite. DMI in contrast shows a significant difference in Raman spectra. A notable increase in I_D/I_G from 0.1,

typical of graphite, to an average value of 0.3 and high values of 0.6 is consistent with a decrease in flake size from the high energy shear process; occurring concurrently with an increase in I_{2D}/I_G caused by the exfoliation of flakes. This increase is modest, so much of the material remains un-exfoliated but sufficient delamination has occurred to increase I_{2D}/I_G.

NMP, the standard solvent used for graphene exfoliation, shows a substantial D peak, but the sharp lineshapes and visible D' peak show the flake size has decreased without substantial damage to the conjugated system as would be expected from graphene oxide. I_{2D}/I_G has also increased and the 2D peak is a single peak shape, not the doublet signature from graphite. Thus, the Raman data shows the exfoliation expected from NMP. Comparison with PRL, the new solvent, reveals approximately the same increase in I_{2D}/I_G so the same exfoliation is expected. However, the sharper distribution seen in the histogram as a tight cluster at a lower I_D/I_G value indicates the PRL exfoliated material may contain larger graphene flakes.

For the comparison of solvents against the starting graphite, a typical 3D histogram of unexfoliated graphite is shown below, **Figure S5.2**.

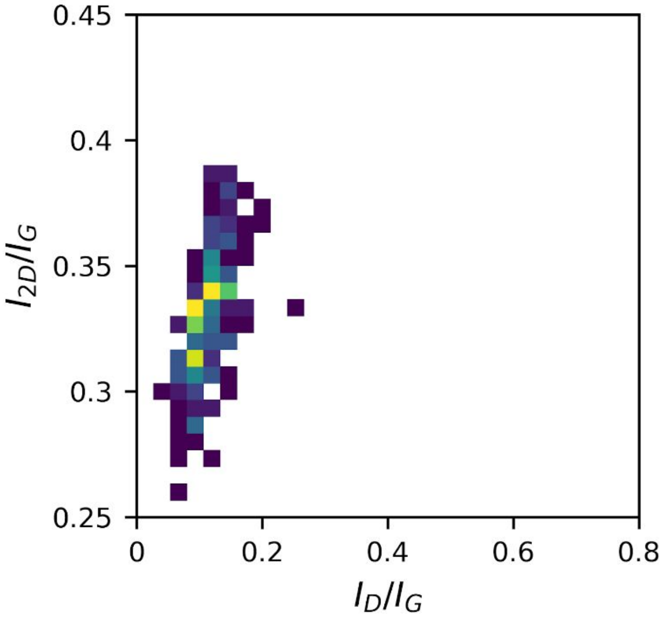

Figure S5.2: Statistical Raman data set from graphite control sample. *3D bivariate histogram showing the statistical Raman data from the starting flake graphite before exfoliation. Bin boundaries are defined by the axes and bin occupancy by the coloured heat map, yellow (light) = more populated bin.*

5.3 Flake size

TEM images were acquired using a JEOL 2100F FEG TEM operating at 80 kV. Samples were dispersed in ethanol solution then dropped onto holey carbon on a 300-mesh copper grid. Flakes used for size measurements were individual flakes without folding and were measured according to National Physical Laboratory guidance;[29] calculating a mean of the longest length and perpendicular width value.

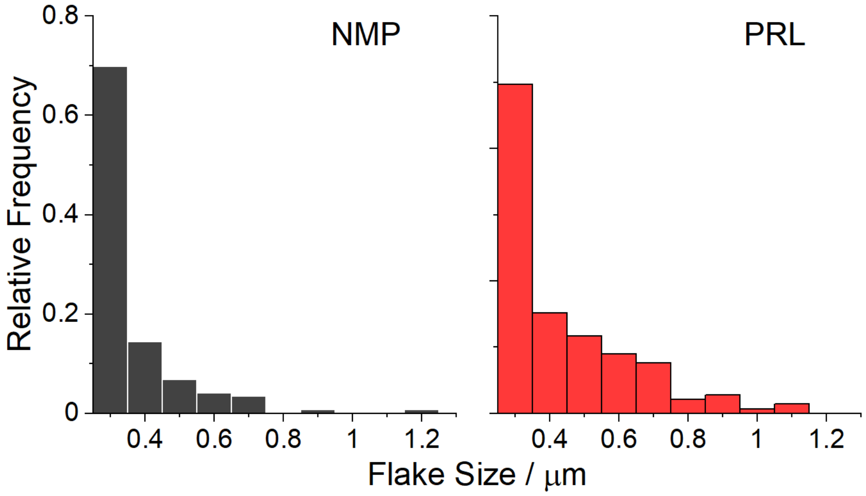

Figure S5.3**: **Histogram of lateral flakes sizes from NMP and PRL exfoliated graphene, measured with TEM. *Comparison of graphene flake sizes, PRL exfoliated graphene has a high proportion of flakes greater than 450 nm in lateral dimensions.*

5.4 Absorbance and concentration

Optical absorbance was measured with a Cary 5000 UV-vis-NIR using 2mm pathlength quartz cuvettes to minimise scattering from flakes; concentration values were calculated using the extinction coefficient reported at 660 nm (α_{660nm} = 2460 ml mg^{-1} m^{-1}).[30]

The comparable concentration of NMP to PRL and larger flake size of PRL may be promising for future graphene production. It should be acknowledged the greater viscosity of PRL (158 mP)[31] compared with NMP (18 mP)[32] may contribute to this concentration as the sedimentation during centrifugation may be reduced by a more viscous solvent. However, the exfoliated graphene evidenced by microscopy and Raman spectroscopy is consistent with the insights gained from MD.

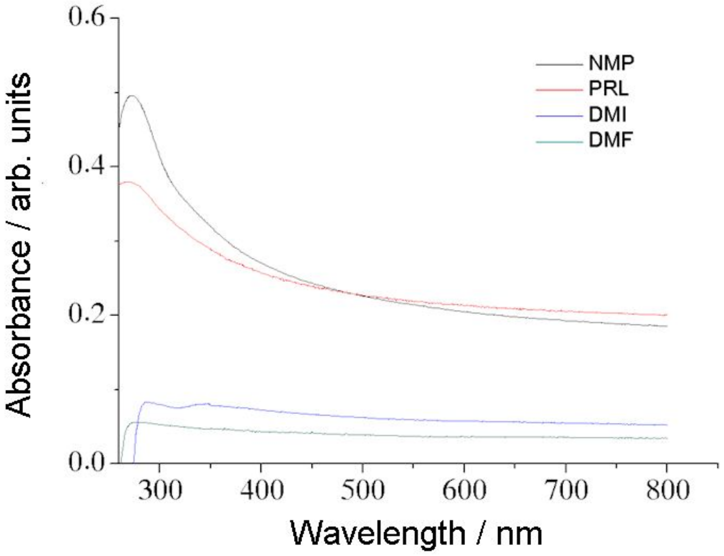

Figure S5.4: UV-vis-nIR absorbance spectra from all non-transparent graphene dispersions. *The concentration produced from cyclopentanone was insufficient.*

Table S5.4: Graphene concentrations calculated from optical absorbance. *Graphene concentrations calculated from the UV-vis-nIR absorbance spectra (Figure S5.4) using* $\alpha_{660nm} = 2460\ ml\ mg^{-1}\ m^{-1}$.[30]

Solvent	Concentration, mg mL^{-1}
CPN	N/A
DMF	0.015
DMI	0.023
NMP	0.080
PRL	0.085

5.5 AFM

AFM images were collected with an AIST-NT SPM SmartSPM™-1000 operating in non-contact mode with silicon tips, samples were washed in ethanol by repeated dispersion and centrifugation before being drop cast onto silicon for imaging. Example images and height profiles are shown below.

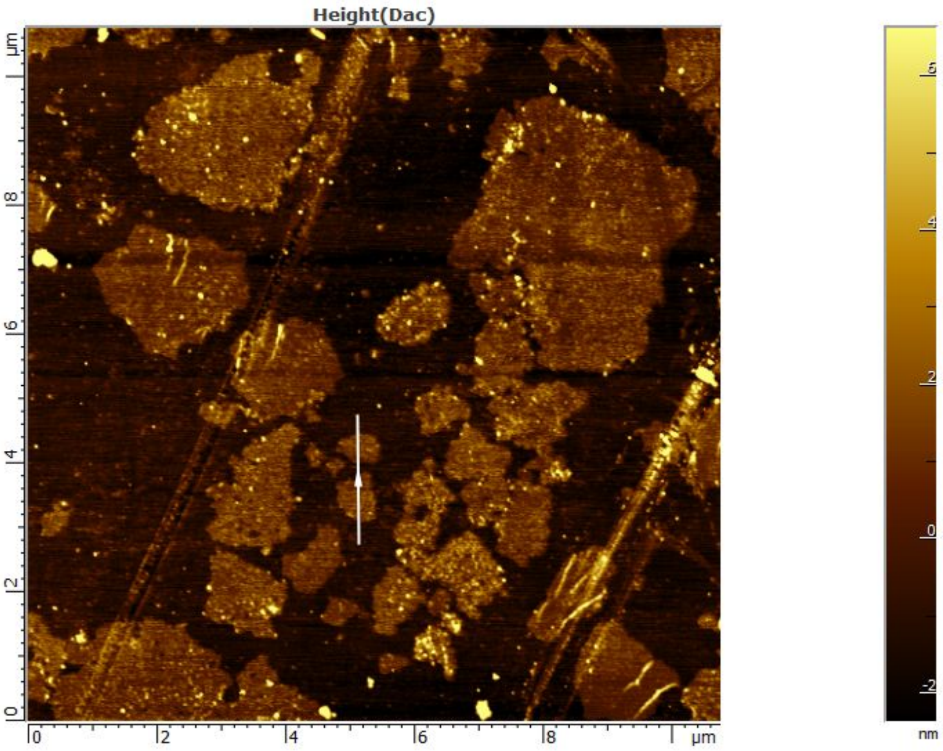

Figure S5.5.1: AFM height image of graphene exfoliated in NMP. *The location of the height profile shown below, Figure S5.5.2 is highlighted by the white line.*

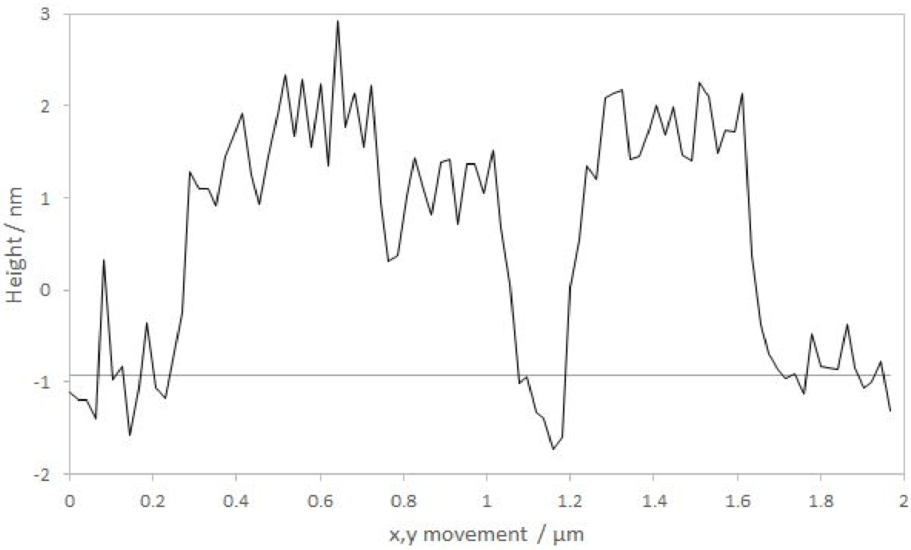

Figure S5.5.2: AFM height profile from two flakes exfoliated in NMP. *The location from which the height profile is measured is shown in the image above.*

AFM micrographs of the graphene exfoliated in NMP show individual flakes ranging in size from 3 – 4 µm for example the larger flake on the right; to smaller flakes 200 – 500 nm in lateral size. The height profile measured shows few layer graphene ~3 nm in measured height; unfortunately, without clear step heights the number of layers cannot be ascertained.

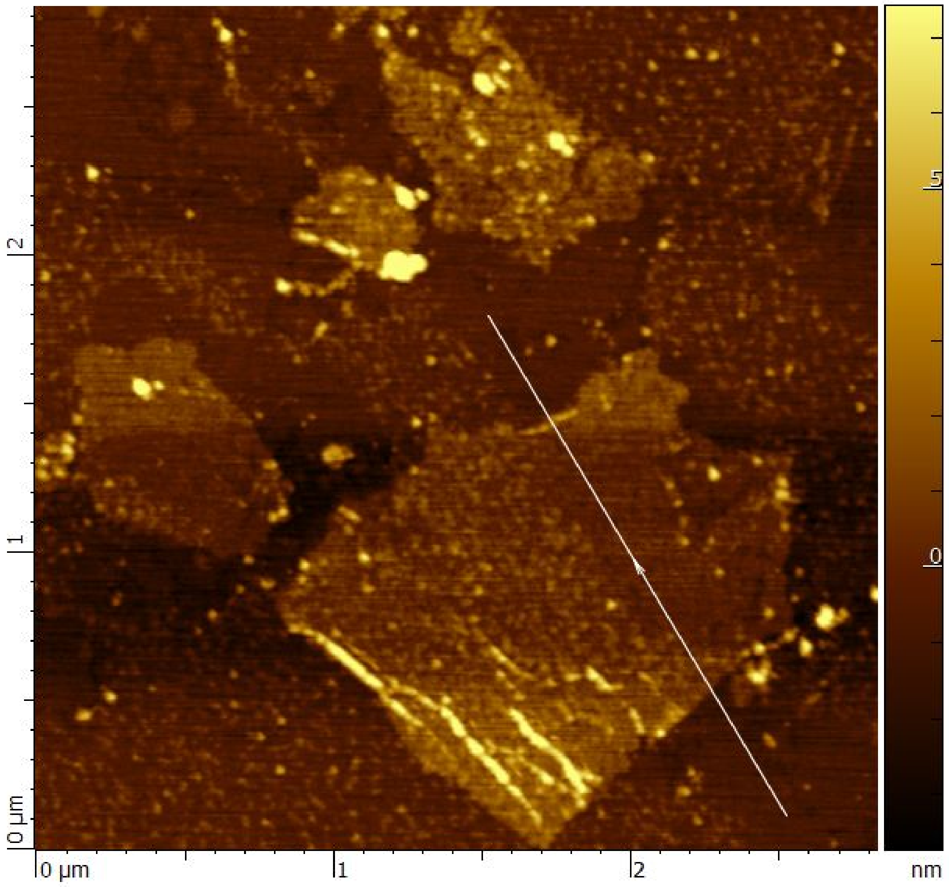

Figure S5.5.3: AFM image of a graphene flakes exfoliated in PRL. *Wrinkles of the graphene flake cause the high lines. The location of the height profile shown below in Figure S5.5.4 is denoted by the white line.*

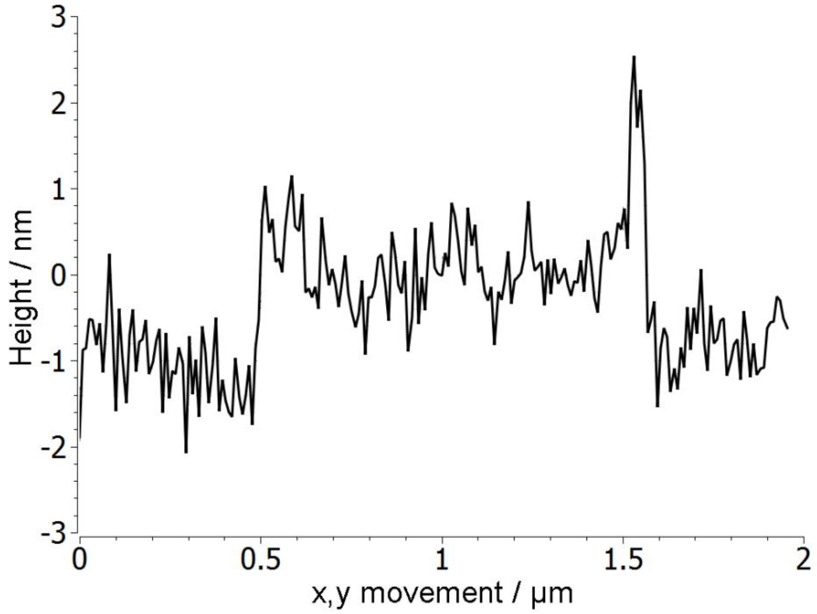

Figure S5.5.4: AFM height profile from the exfoliated graphene flake. *The location from which the height profile is measured is shown in the image above, Figure S5.5.3.*

AFM images of the graphene exfoliated by PRL show individual flakes; Figure S5.5.3 shows a single larger flake folded over on the top edge resulting in a large height at the edge of the sheet, seen in Figure S5.5.4. The presence of few layer graphene is inferred from the height profile (Figure S5.5.5) of the micrograph in Figure 4, main paper. Multiple individual flakes are observed with consistent steps of ~2 nm in height between the flakes. Such height steps are larger than the ~1 nm typical of single layer graphene measured by AFM, suggesting few-layer graphene folded at the edge doubling the apparent height.

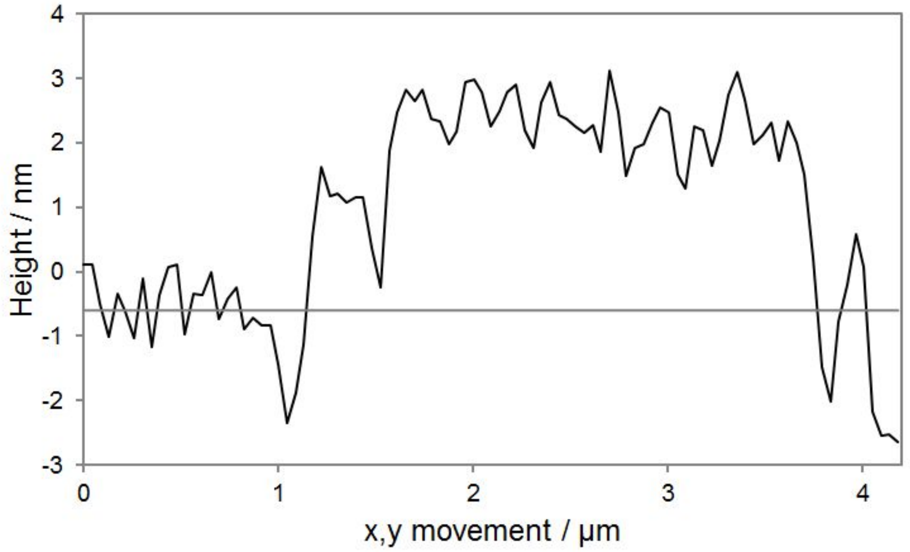

Figure S5.5.5: AFM height profile from the exfoliated graphene flake in PRL. *The location from which the height profile is measured is shown in Figure 4 within the main paper.*

5.6 TEM

TEM images were acquired using a JEOL 2100F FEG TEM operating at 80 kV. Samples were dispersed in ethanol solution then dropped onto holey carbon on a 300-mesh copper grid. Flake lateral size measurements were made following the National Physical Laboratory protocol;[29] calculating a mean of the longest length and perpendicular width value.

Additional TEM images of graphene flakes from both NMP and PRL shown below.

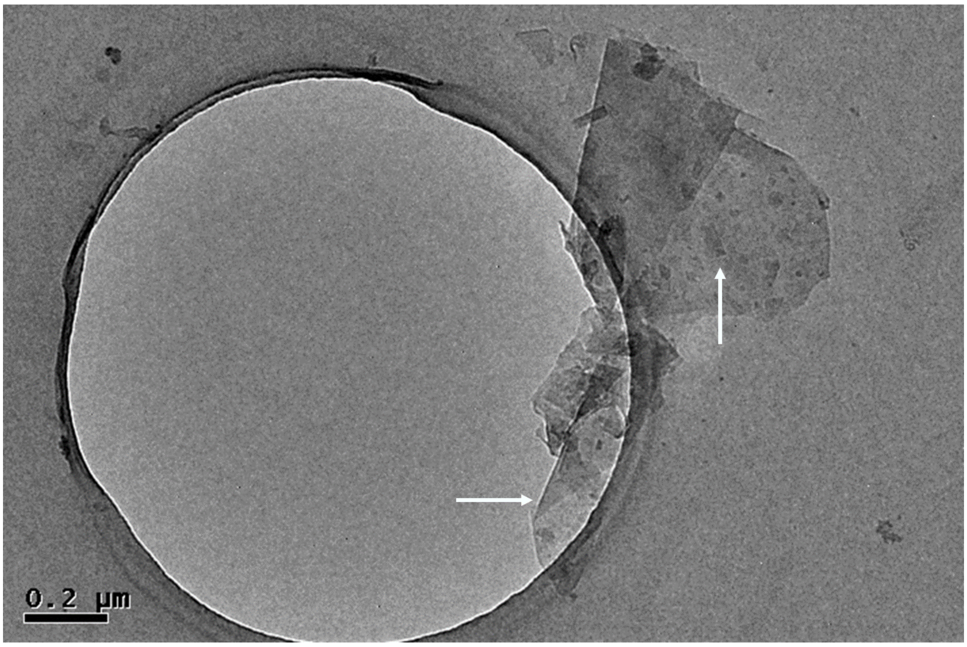

Figure S5.6.1: TEM of exfoliated graphene flakes from NMP. *Small flakes and fragments are present on the main graphene flake, highlighted by the topmost vertical arrow, while the flexibility to folding and overlaid sheets indicates few-layer graphene. Example fold highlighted by the left horizontal arrow.*

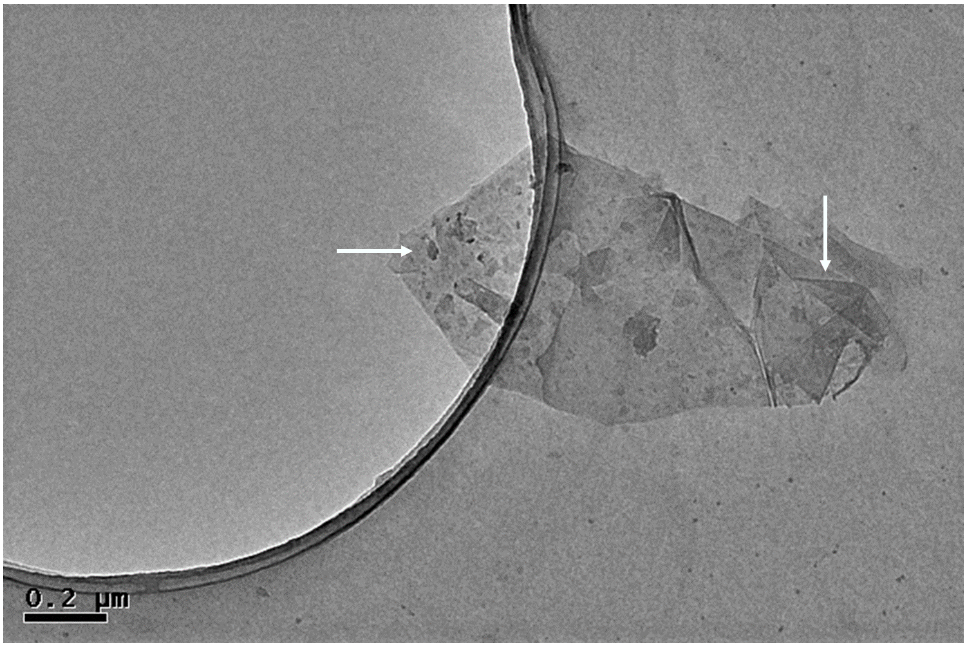

Figure S5.6.2: TEM of exfoliated graphene flake from NMP. *Small flakes and fragments are present on the main graphene flake, highlighted by the leftmost arrow, while the flexibility to folding and overlaid sheets indicates few-layer graphene. Example fold highlighted by the rightmost arrow.*

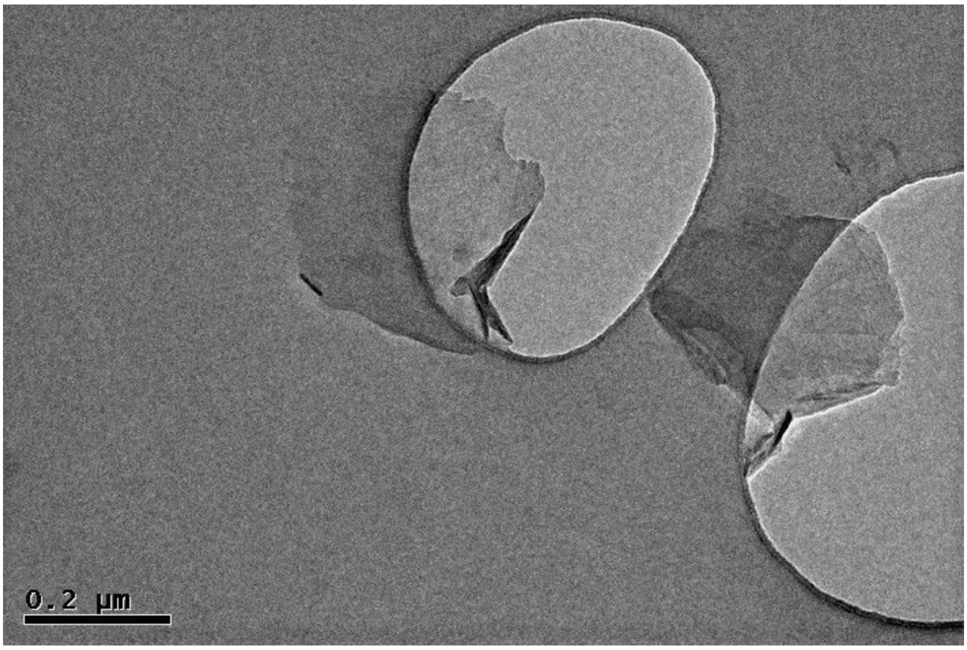

Figure S5.6.3: TEM of exfoliated graphene flake from PRL. *Large flakes of exfoliated graphene.*

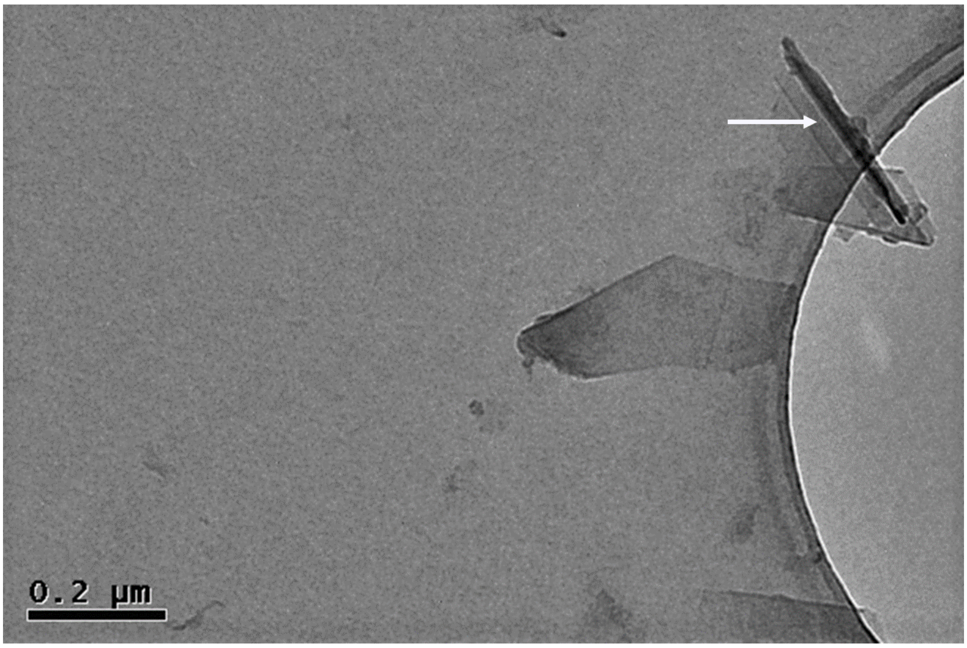

Figure S5.6.4: TEM of exfoliated graphene flakes from PRL. *Flakes of thin graphene exfoliated in PRL. The top flake shows signs of scrolling and clean folds highlighted by the white arrow.*

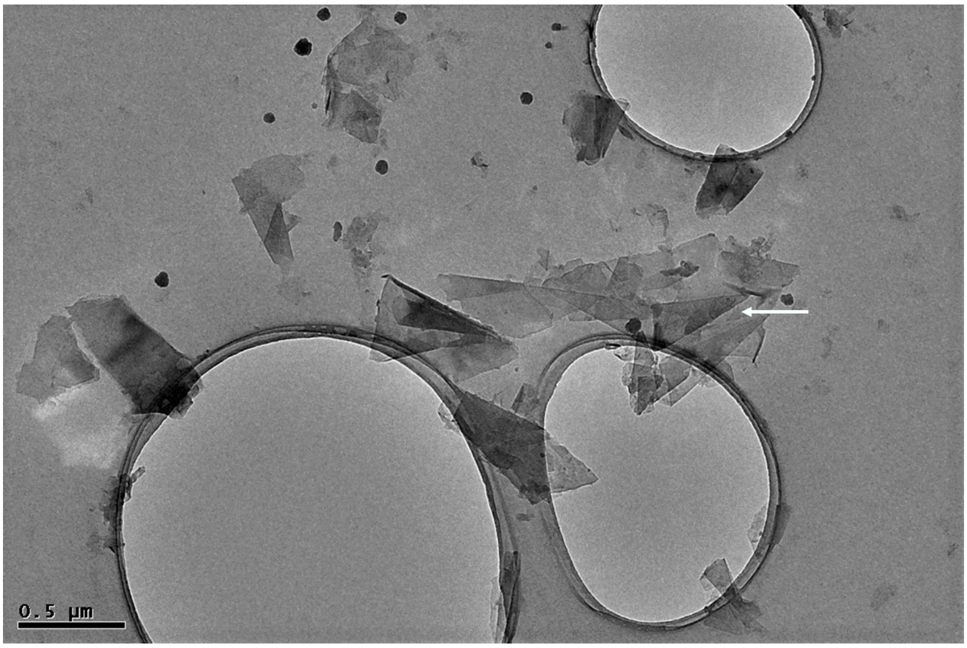

Figure S5.6.5: TEM of exfoliated graphene flakes from PRL. *Multiple graphene flakes of different sizes, many thin sheets seen overlaid and folded. One example highlighted by white arrow.*

These additional micrographs show the typical morphology of many of the flakes; here the difference in lateral flake size discussed in the main paper can be seen visually. Whereas the NMP flakes have many smaller fragments decorated over the surface the PRL graphene flakes were observed to be cleaner and mostly free from debris.

The flexible nature of graphene flakes makes they regularly fold and overlay, forming weak interactions between the sheets and minimising any unfavourable dangling bonds. At the fold, since the carbon layers are oriented perpendicular to the electron beam it is possible to count the carbon layers seen as sharply contrasting lines.

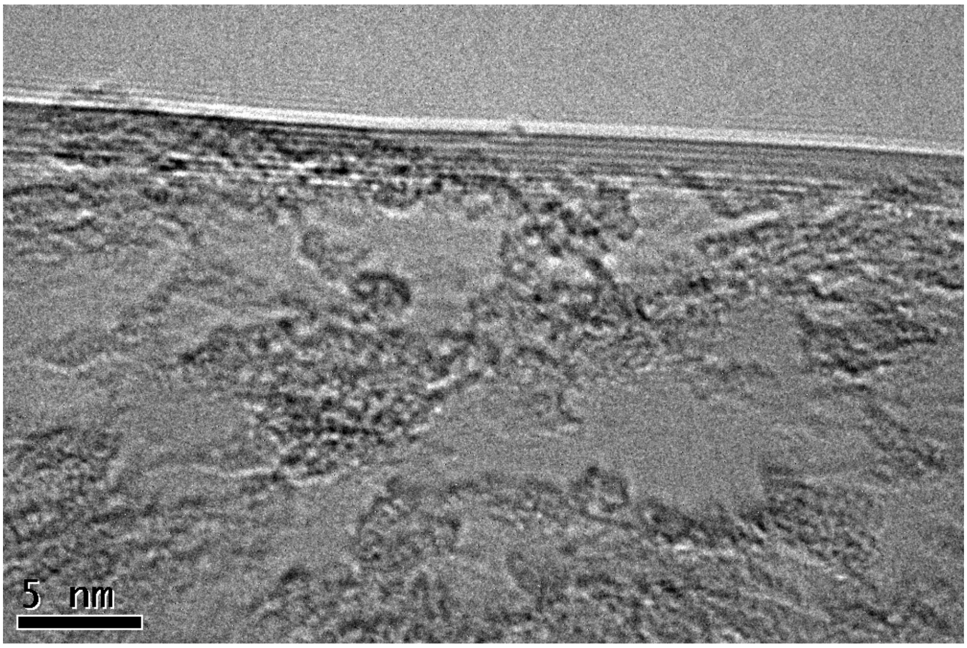

Figure S5.6.6: HRTEM of the edge of exfoliated graphene from PRL. *Here the edge of a graphene flake is observed folded over with seven carbon layers visible along the edge.*

6. Validating Number of Raman Data Points

To ensure the number of spectra collected was sufficient, a statistical analysis was undertaken. Plotting the convergence of key summary statistics: mean, upper and lower quartiles, and 10th and 90th percentiles as more data points were collected highlights the importance of each new spectra. This was also visualised with a bootstrap analysis; in effect plotting the distribution from randomly generated sub-sets from the total data set. By considering the difference between these sub-set distributions, the impact of under analysing can be seen.

When the bootstrap distributions are consistent and the summary statistics stop changing with new data points, the data set is said to have converged and the number of points measured is sufficient for statistical analysis.

6.1 Graphite

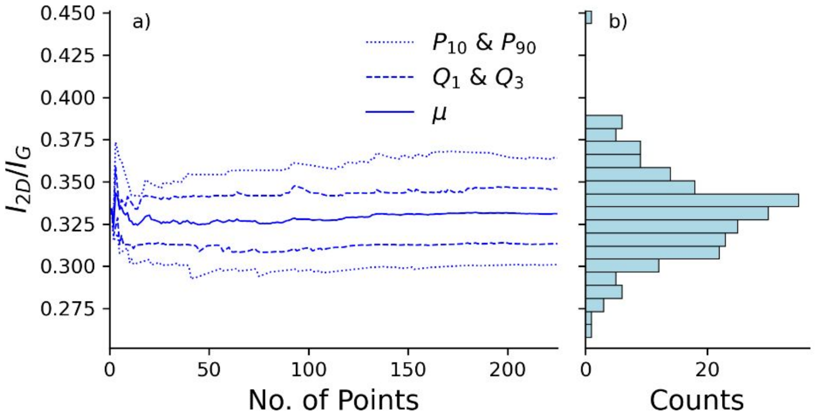

Figure S6.1.1: Convergence plot of the starting graphite. *a) the change in summary statistics representing the distribution of I_{2D}/I_G values as more data points are added. b) The final distribution shown as a horizontal histogram for reference.*

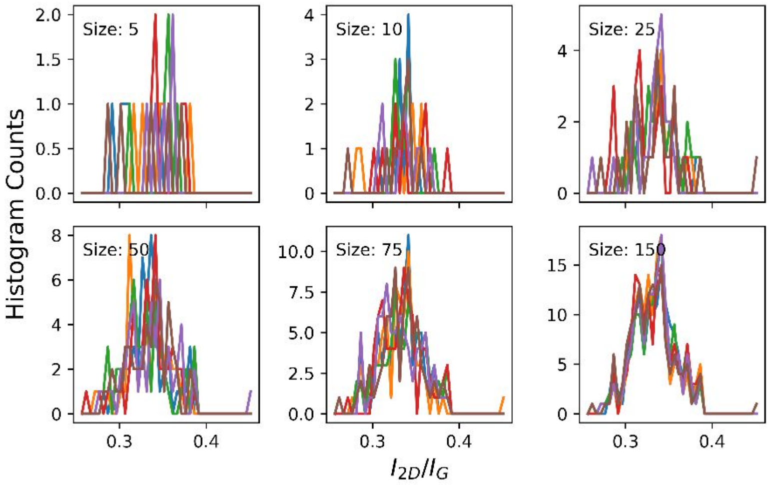

Figure S6.1.2: Panel showing bootstrap analysis of the starting graphite. *Each panel shows six examples, shown in different colours, of distributions of I_{2D}/I_G produced from these sub-sets. These show the convergence to a uniform distribution as the sub-set size increases.*

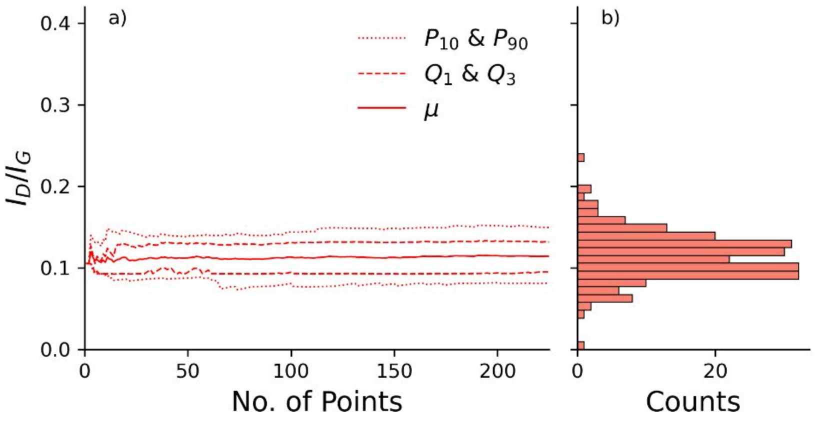

Figure S6.1.3: Convergence plot of the starting graphite. *a) the change in summary statistics representing the distribution of I_D/I_G values as more data points are added. b) The final distribution shown as a horizontal histogram for reference.*

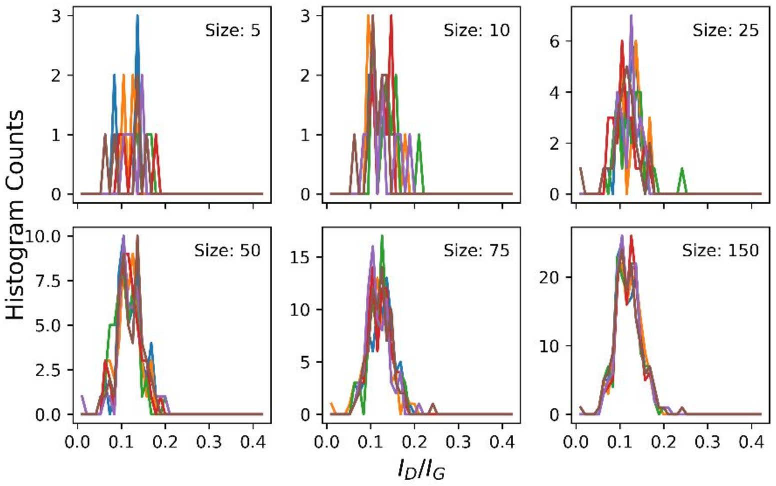

Figure S6.1.4: Panel showing bootstrap analysis of the starting graphite. *Each panel shows six examples, shown in different colours, of distributions of I_D/I_G produced from these sub-sets. These show the convergence to a uniform distribution as the sub-set size increases.*

6.2 CPN

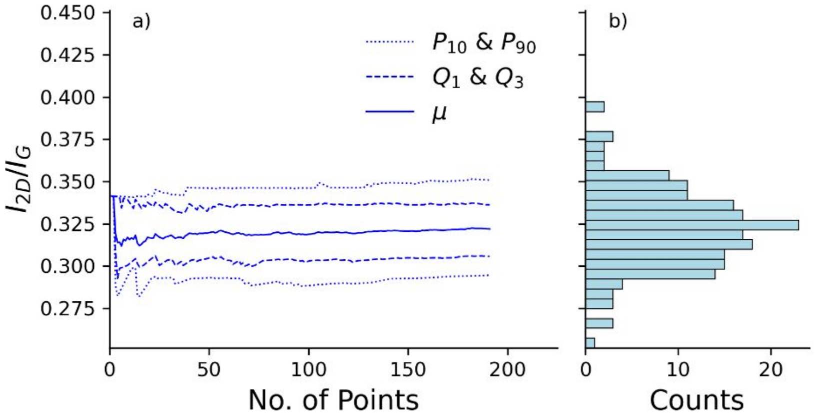

Figure S6.2.1: Convergence plot of the CPN exfoliated graphene. *a) the change in summary statistics representing the distribution of I_{2D}/I_G values as more data points are added. b) The final distribution shown as a horizontal histogram for reference.*

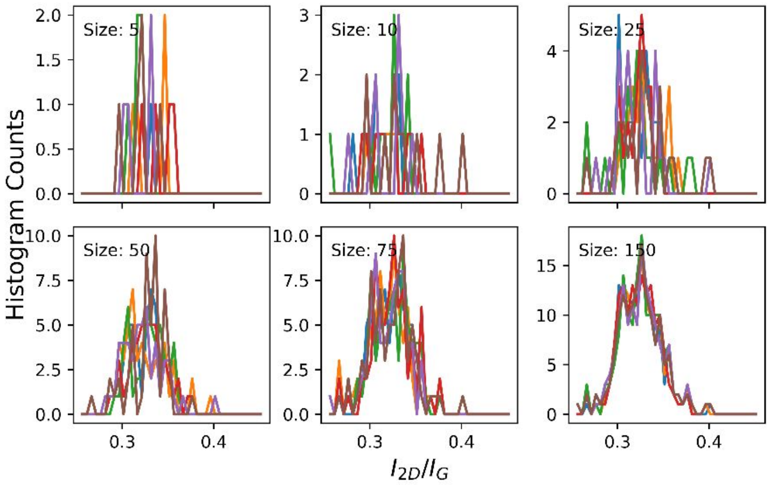

Figure S6.2.2: Panel showing bootstrap analysis of the CPN exfoliated graphene. *Each panel shows six examples, shown in different colours, of distributions of I_{2D}/I_G produced from these sub-sets. These show the convergence to a uniform distribution as the sub-set size increases.*

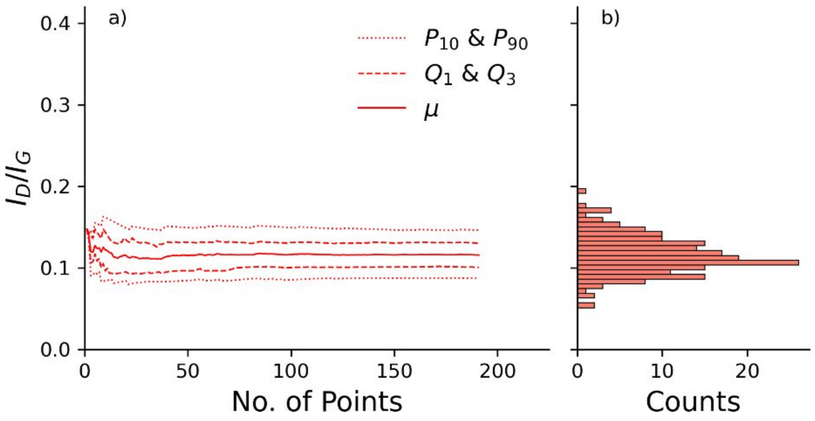

Figure S6.2.3: Convergence plot of the CPN exfoliated graphene. *a) the change in summary statistics representing the distribution of I_D/I_G values as more data points are added. b) The final distribution shown as a horizontal histogram for reference.*

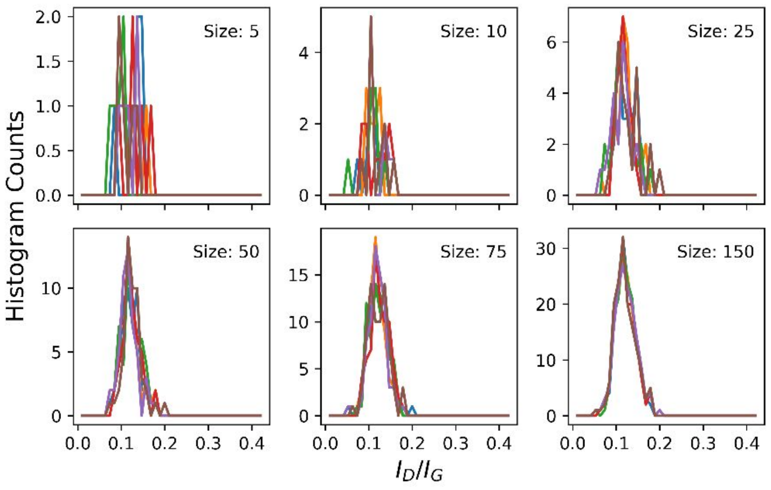

Figure S6.2.4: Panel showing bootstrap analysis of the CPN exfoliated graphene. *Each panel shows six examples, shown in different colours, of distributions of I_D/I_G produced from these sub-sets. These show the convergence to a uniform distribution as the sub-set size increases.*

6.3 DMF

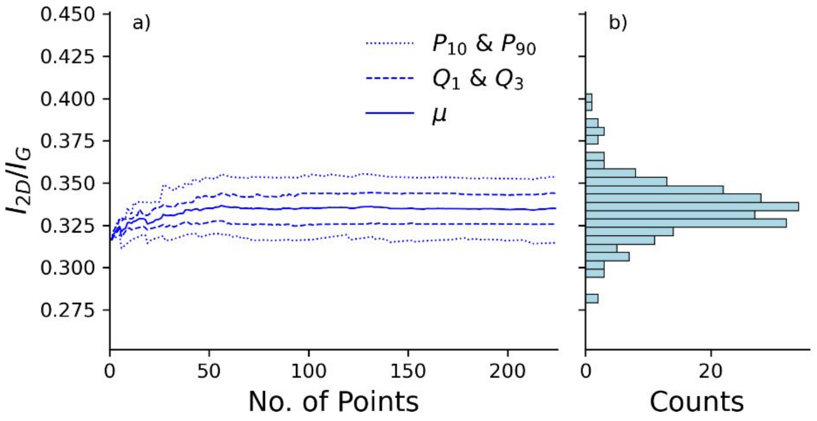

Figure S6.3.1: Convergence plot of the DMF exfoliated graphene. *a) the change in summary statistics representing the distribution of I_{2D}/I_G values as more data points are added. b) The final distribution shown as a horizontal histogram for reference.*

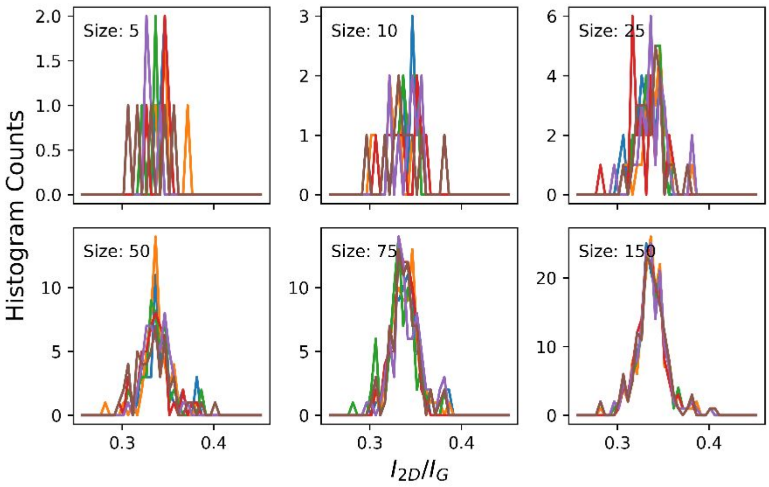

Figure S6.3.2: Panel showing bootstrap analysis of the DMF exfoliated graphene. *Each panel shows six examples, shown in different colours, of distributions of I_{2D}/I_G produced from these sub-sets. These show the convergence to a uniform distribution as the sub-set size increases.*

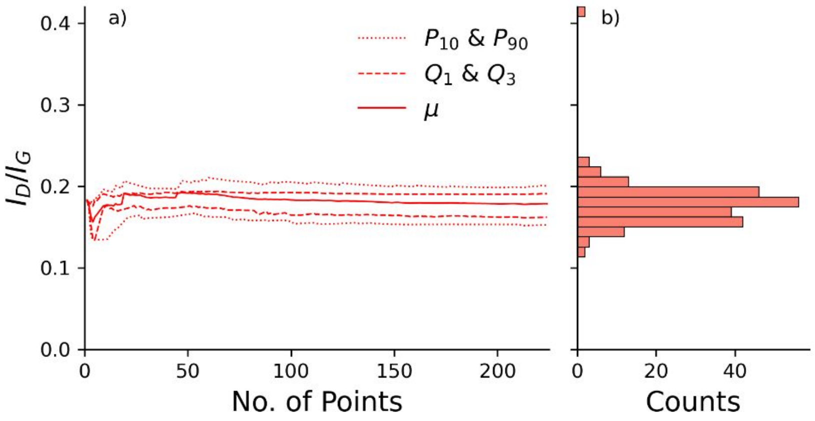

Figure S6.3.3: Convergence plot of the DMF exfoliated graphene. *a) the change in summary statistics representing the distribution of I_D/I_G values as more data points are added. b) The final distribution shown as a horizontal histogram for reference.*

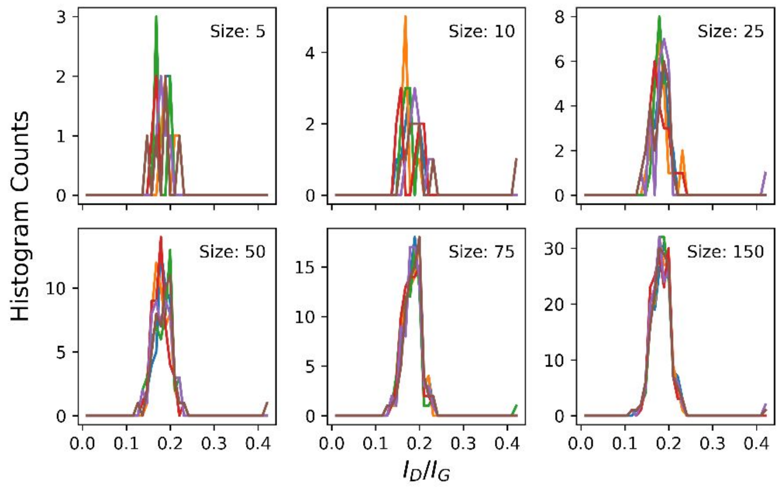

Figure S6.3.4: Panel showing bootstrap analysis of the DMF exfoliated graphene. *Each panel shows six examples, shown in different colours, of distributions of I_D/I_G produced from these sub-sets. These show the convergence to a uniform distribution as the sub-set size increases.*

6.4 DMI

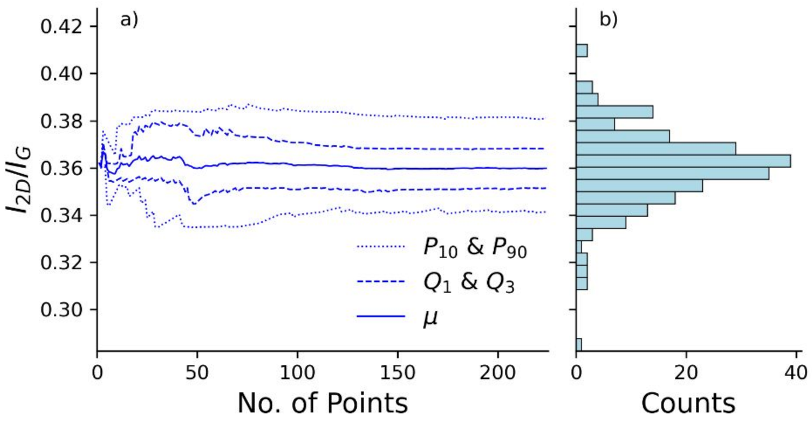

Figure S6.4.1: Convergence plot of the DMI exfoliated graphene. *a) the change in summary statistics representing the distribution of I_{2D}/I_G values as more data points are added. b) The final distribution shown as a horizontal histogram for reference.*

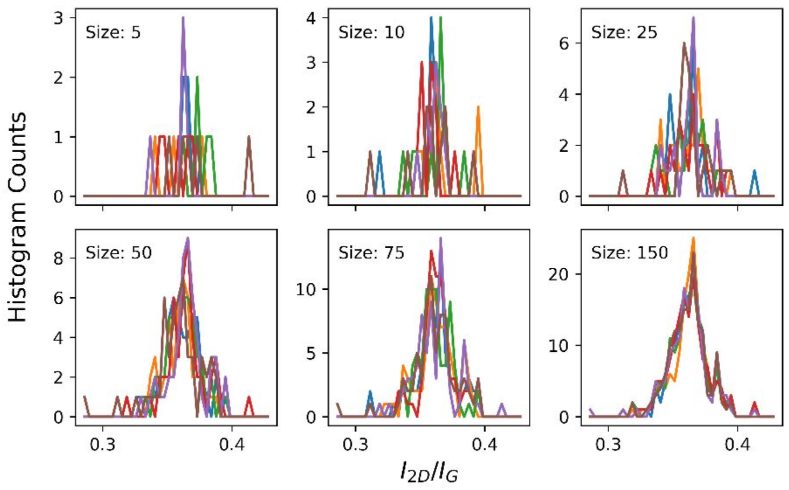

Figure S6.4.2: Panel showing bootstrap analysis of the DMI exfoliated graphene. *Each panel shows six examples, shown in different colours, of distributions of I_{2D}/I_G produced from these sub-sets. These show the convergence to a uniform distribution as the sub-set size increases.*

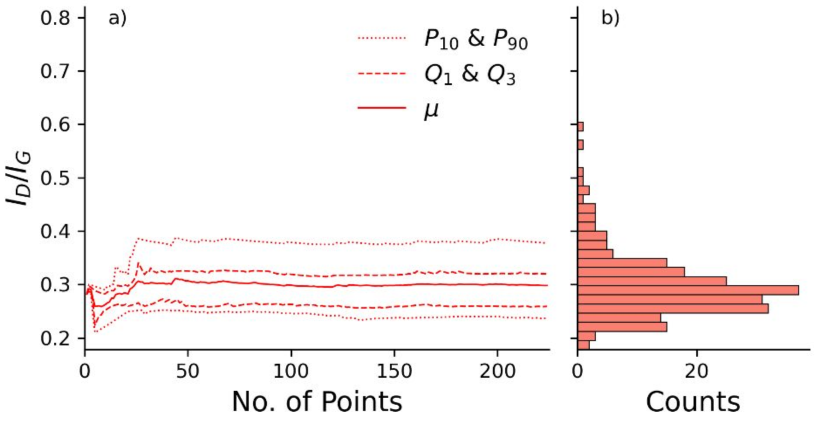

Figure S6.4.3: Convergence plot of the DMI exfoliated graphene. *a) the change in summary statistics representing the distribution of I_D/I_G values as more data points are added. b) The final distribution shown as a horizontal histogram for reference.*

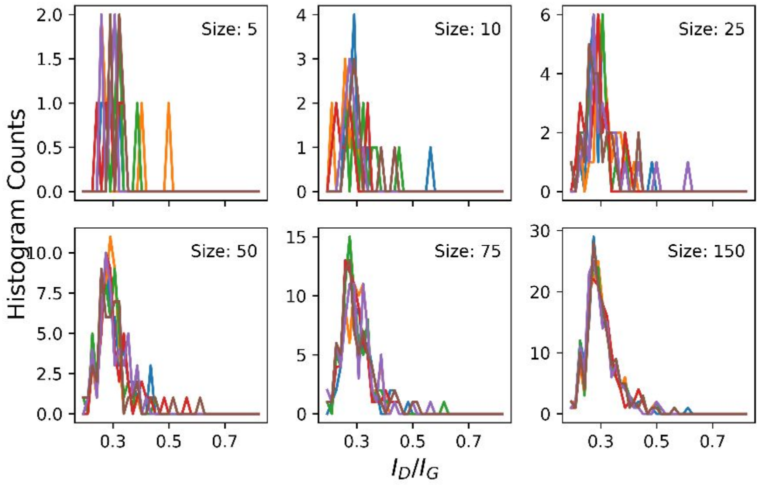

Figure S6.4.4: Panel showing bootstrap analysis of the DMI exfoliated graphene. *Each panel shows six examples, shown in different colours, of distributions of I_D/I_G produced from these sub-sets. These show the convergence to a uniform distribution as the sub-set size increases.*

6.5 NMP

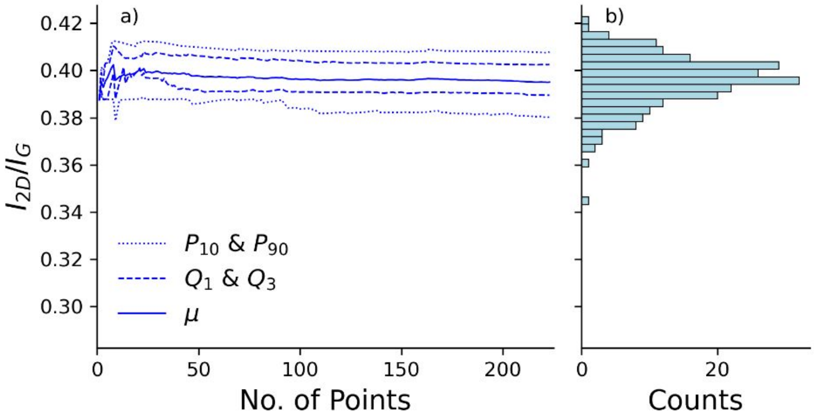

Figure S6.5.1: Convergence plot of the NMP exfoliated graphene. *a) the change in summary statistics representing the distribution of I_{2D}/I_G values as more data points are added. b) The final distribution shown as a horizontal histogram for reference.*

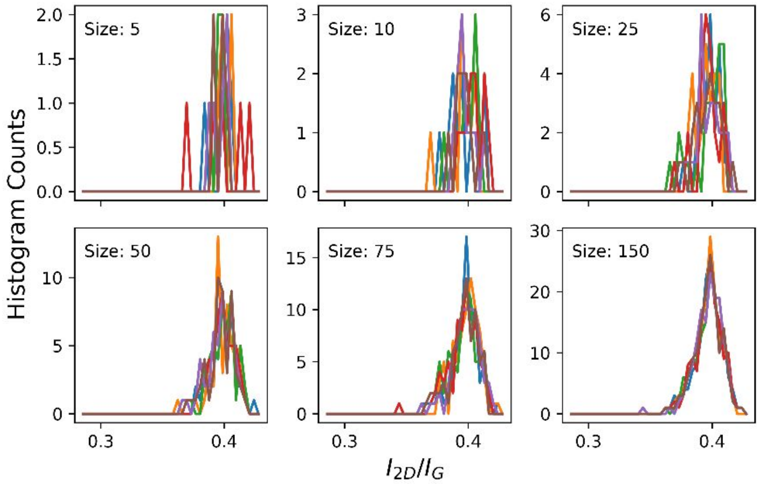

Figure S6.5.2: Panel showing bootstrap analysis of the NMP exfoliated graphene. *Each panel shows six examples, shown in different colours, of distributions of I_{2D}/I_G produced from these sub-sets. These show the convergence to a uniform distribution as the sub-set size increases.*

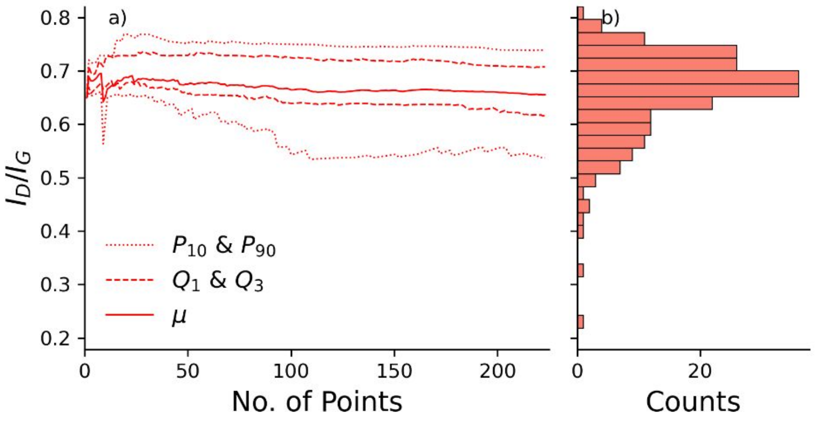

Figure S6.5.3: Convergence plot of the NMP exfoliated graphene. *a) the change in summary statistics representing the distribution of I_D/I_G values as more data points are added. b) The final distribution shown as a horizontal histogram for reference.*

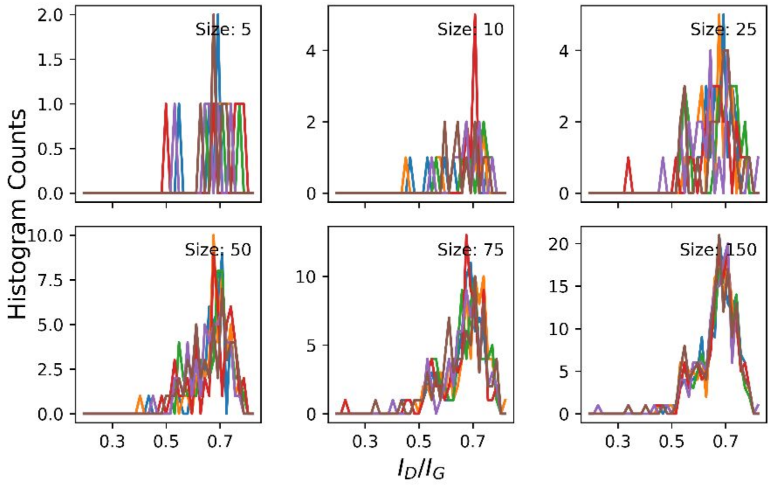

Figure S6.5.4: Panel showing bootstrap analysis of the NMP exfoliated graphene. *Each panel shows six examples, shown in different colours, of distributions of I_D/I_G produced from these sub-sets. These show the convergence to a uniform distribution as the sub-set size increases.*

6.6 PRL

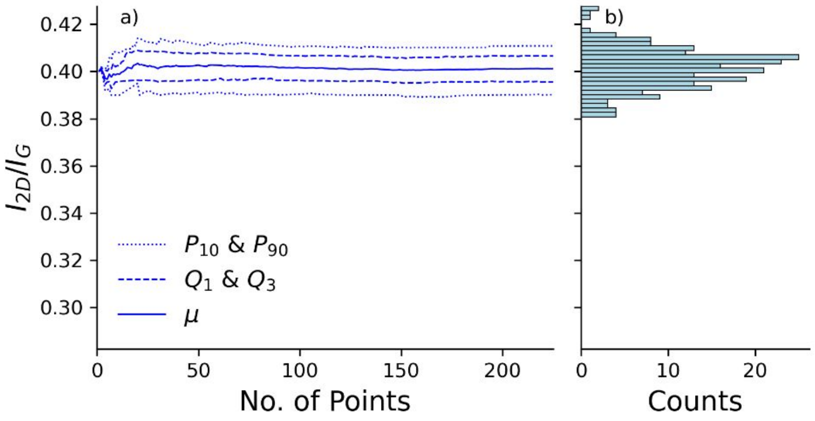

Figure S6.6.1: Convergence plot of the PRL exfoliated graphene. *a) the change in summary statistics representing the distribution of I_{2D}/I_G values as more data points are added. b) The final distribution shown as a horizontal histogram for reference.*

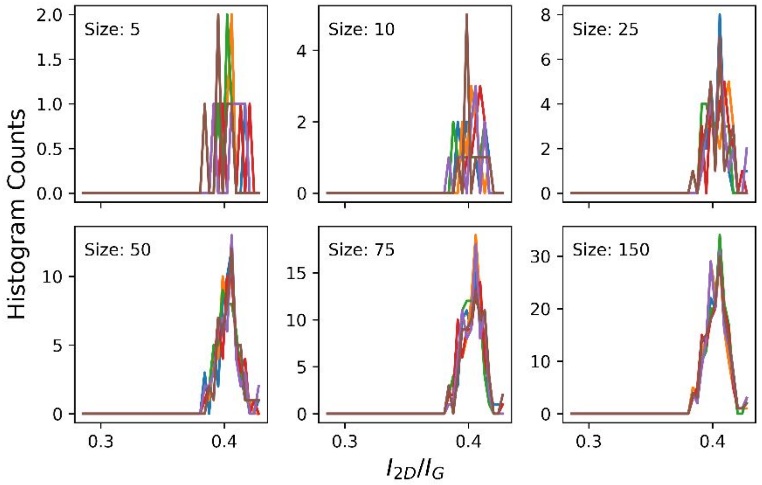

Figure S6.6.2: Panel showing bootstrap analysis of the PRL exfoliated graphene. *Each panel shows six examples, shown in different colours, of distributions of I_{2D}/I_G produced from these sub-sets. These show the convergence to a uniform distribution as the sub-set size increases.*

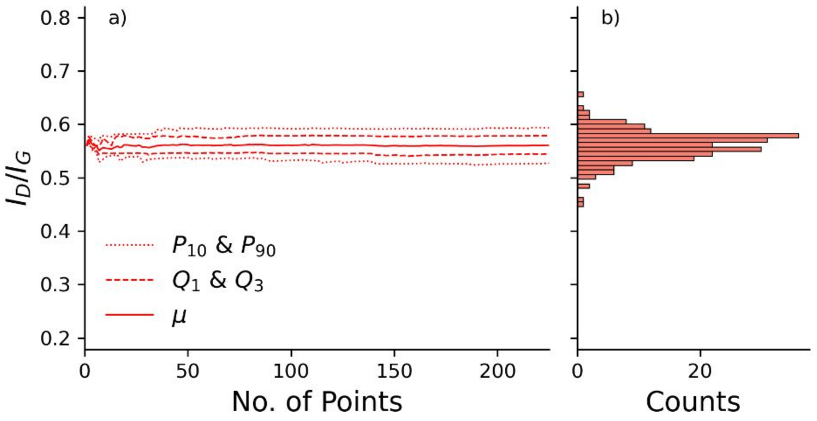

Figure S6.6.3: Convergence plot of the PRL exfoliated graphene. *a) the change in summary statistics representing the distribution of I_D/I_G values as more data points are added. b) The final distribution shown as a horizontal histogram for reference.*

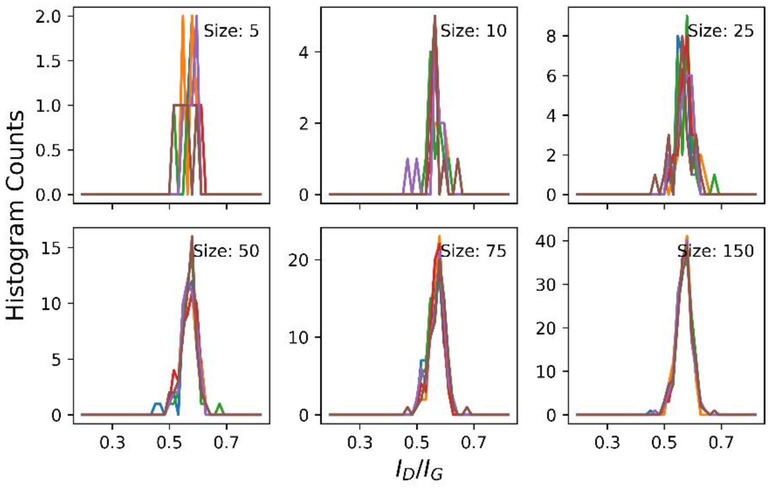

Figure S6.6.4: Panel showing bootstrap analysis of the PRL exfoliated graphene. *Each panel shows six examples, shown in different colours, of distributions of I_D/I_G produced from these sub-sets. These show the convergence to a uniform distribution as the sub-set size increases.*

References

(1) Schiettecatte, P.; Rousaki, A.; Vandenabeele, P.; Geiregat, P.; Hens, Z. Liquid-Phase Exfoliation of Rhenium Disulfide by Solubility Parameter Matching. *Langmuir* **2020**, *36* (51), 15493–15500. https://doi.org/10.1021/acs.langmuir.0c02517.

(2) Hernandez, Y.; Lotya, M.; Rickard, D.; Bergin, S. D.; Coleman, J. N. Measurement of Multicomponent Solubility Parameters for Graphene Facilitates Solvent Discovery. *Langmuir* **2010**, *26* (5), 3208–3213. https://doi.org/10.1021/la903188a.

(3) Yaws, Carl. L. *Thermophysical Properties of Chemicals and Hydrocarbons*. Elsevier: Norwich, NY, 2009.

(4) Habrdová, K.; Hovorka, Š.; Bartovská, L. Concentration Dependence of Surface Tension for Very Dilute Aqueous Solutions of Organic Nonelectrolytes. *Journal of Chemical & Engineering Data* **2004**, *49* (4), 1003–1007. https://doi.org/10.1021/je049955d.

(5) Hansen, C. *Hansen Solubility Parameters: A User's Handbook*, Second.; CRC Press: Boca Raton, Fla, 2007.

(6) Tkachev, S.; Monteiro, M.; Santos, J.; Placidi, E.; Hassine, M. ben; Marques, P.; Ferreira, P.; Alpuim, P.; Capasso, A. Environmentally Friendly Graphene Inks for Touch Screen Sensors. *Advanced Functional Materials* **2021**, *31* (33), 2103287. https://doi.org/https://doi.org/10.1002/adfm.202103287.

(7) Paton, K. R.; Varrla, E.; Backes, C.; Smith, R. J.; Khan, U.; O'Neill, A.; Boland, C.; Lotya, M.; Istrate, O. M.; King, P.; Higgins, T.; Barwich, S.; May, P.; Puczkarski, P.; Ahmed, I.; Moebius, M.; Pettersson, H.; Long, E.; Coelho, J.; O'Brien, S. E.; McGuire, E. K.; Sanchez, B. M.; Duesberg, G. S.; McEvoy, N.; Pennycook, T. J.; Downing, C.; Crossley, A.; Nicolosi, V.; Coleman, J. N. Scalable Production of Large Quantities of Defect-Free Few-Layer Graphene by Shear Exfoliation in Liquids. *Nature Materials* **2014**, *13* (6), 624–630. https://doi.org/10.1038/nmat3944.

(8) Tran, T. S.; Park, S. J.; Yoo, S. S.; Lee, T.-R.; Kim, T. High Shear-Induced Exfoliation of Graphite into High Quality Graphene by Taylor–Couette Flow. *RSC Adv.* **2016**, *6* (15), 12003–12008. https://doi.org/10.1039/C5RA22273G.

(9) Liu, L.; Shen, Z.; Yi, M.; Zhang, X.; Ma, S. A Green, Rapid and Size-Controlled Production of High-Quality Graphene Sheets by Hydrodynamic Forces. *RSC Adv.* **2014**, *4* (69), 36464–36470. https://doi.org/10.1039/C4RA05635C.

(10) Wasim Akhtar, M.; Park, C. W.; Kim, Y. S.; Kim, J. S. Facile Large Scale Production of Few-Layer Graphene Sheets by Shear Exfoliation in Volatile Solvent. *Journal of Nanoscience and Nanotechnology* **2015**, *15* (12), 9624–9629. https://doi.org/10.1166/jnn.2015.11119.

(11) Phiri, J.; Gane, P.; Maloney, T. C. High-Concentration Shear-Exfoliated Colloidal Dispersion of Surfactant–Polymer-Stabilized Few-Layer Graphene Sheets. *Journal of Materials Science* **2017**, *52* (13), 8321–8337. https://doi.org/10.1007/s10853-017-1049-y.

(12) Khanam, Z.; Liu, J.; Song, S. High-Concentration Graphene Dispersions Prepared via Exfoliation of Graphite in PVA/H2O Green Solvent System Using High-Shear Forces. *Journal of Nanoparticle Research* **2021**, *23* (8), 170. https://doi.org/10.1007/s11051-021-05294-2.

(13) Lund, S.; Kauppila, J.; Sirkiä, S.; Palosaari, J.; Eklund, O.; Latonen, R.-M.; Smått, J.-H.; Peltonen, J.; Lindfors, T. Fast High-Shear Exfoliation of Natural Flake Graphite with Temperature Control and High Yield. *Carbon N Y* **2021**, *174*, 123–131. https://doi.org/https://doi.org/10.1016/j.carbon.2020.11.094.

(14) del Río, F.; Boado, M. G.; Rama, A.; Guitián, F. A Comparative Study on Different Aqueous-Phase Graphite Exfoliation Methods for Few-Layer Graphene Production and Its Application in Alumina Matrix Composites. *J Eur Ceram Soc* **2017**, *37* (12), 3681–3693. https://doi.org/https://doi.org/10.1016/j.jeurceramsoc.2017.04.029.

(15) Simon, D. A.; Bischoff, E.; Buonocore, G. G.; Cerruti, P.; Raucci, M. G.; Xia, H.; Schrekker, H. S.; Lavorgna, M.; Ambrosio, L.; Mauler, R. S. Graphene-Based Masterbatch Obtained via Modified Polyvinyl Alcohol Liquid-Shear Exfoliation and Its Application in Enhanced Polymer Composites. *Materials & Design* **2017**, *134*, 103–110. https://doi.org/https://doi.org/10.1016/j.matdes.2017.08.032.

(16) Liang, B.; Liu, K.; Liu, P.; Qian, L.; Zhao, G.; Pan, W.; Chen, C. Organic Salt-Assisted Liquid-Phase Shear Exfoliation of Expanded Graphite into Graphene Nanosheets. *Journal of Materiomics* **2021**, *7* (6), 1181–1189. https://doi.org/https://doi.org/10.1016/j.jmat.2021.03.007.

(17) Stafford, J.; Uzo, N.; Farooq, U.; Favero, S.; Wang, S.; Chen, H.-H.; L'Hermitte, A.; Petit, C.; Matar, O. K. Real-Time Monitoring and Hydrodynamic Scaling of Shear Exfoliated Graphene. *2D Materials* **2021**, *8* (2), 25029. https://doi.org/10.1088/2053-1583/abdf2f.

(18) Varrla, E.; Paton, K. R.; Backes, C.; Harvey, A.; Smith, R. J.; McCauley, J.; Coleman, J. N. Turbulence-Assisted Shear Exfoliation of Graphene Using Household Detergent and a Kitchen Blender. *Nanoscale* **2014**, *6* (20), 11810–11819. https://doi.org/10.1039/C4NR03560G.

(19) Diasio, M. A.; Green, D. L. The Effect of Solvent Viscosity on Production of Few-Layer Graphene from Liquid-Phase Exfoliation of Graphite. *MRS Advances* **2019**, *4* (3), 241–247. https://doi.org/10.1557/adv.2019.13.

(20) Huang, J.; MacKerell Jr, A. D. CHARMM36 All-Atom Additive Protein Force Field: Validation Based on Comparison to NMR Data. *Journal of Computational Chemistry* **2013**, *34* (25), 2135–2145. https://doi.org/https://doi.org/10.1002/jcc.23354.

(21) Vanommeslaeghe, K.; Raman, E. P.; MacKerell, A. D. Automation of the CHARMM General Force Field (CGenFF) II: Assignment of Bonded Parameters and Partial Atomic Charges. *Journal of Chemical Information and Modeling* **2012**, *52* (12), 3155–3168. https://doi.org/10.1021/ci3003649.

(22) Brooks, B. R.; Brooks III, C. L.; Mackerell Jr., A. D.; Nilsson, L.; Petrella, R. J.; Roux, B.; Won, Y.; Archontis, G.; Bartels, C.; Boresch, S.; Caflisch, A.; Caves, L.; Cui, Q.; Dinner, A. R.; Feig, M.; Fischer, S.; Gao, J.; Hodoscek, M.; Im, W.; Kuczera, K.; Lazaridis, T.; Ma, J.; Ovchinnikov, V.; Paci, E.; Pastor, R. W.; Post, C. B.; Pu, J. Z.; Schaefer, M.; Tidor, B.; Venable, R. M.; Woodcock, H. L.; Wu, X.; Yang, W.; York, D. M.; Karplus, M. CHARMM: The Biomolecular Simulation Program. *Journal of Computational Chemistry* **2009**, *30* (10), 1545–1614. https://doi.org/https://doi.org/10.1002/jcc.21287.

(23) Yesselman, J. D.; Price, D. J.; Knight, J. L.; Brooks III, C. L. MATCH: An Atom-Typing Toolset for Molecular Mechanics Force Fields. *Journal of Computational Chemistry* **2012**, *33* (2), 189–202. https://doi.org/https://doi.org/10.1002/jcc.21963.

(24) Bernal, J. D.; Bragg, W. L. The Structure of Graphite. *Proceedings of the Royal Society of London. Series A, Containing Papers of a Mathematical and Physical Character* **1924**, *106* (740), 749–773. https://doi.org/10.1098/rspa.1924.0101.

(25) Abraham, M. J.; Murtola, T.; Schulz, R.; Páll, S.; Smith, J. C.; Hess, B.; Lindahl, E. GROMACS: High Performance Molecular Simulations through Multi-Level Parallelism from Laptops to Supercomputers. *SoftwareX* **2015**, *1–2*, 19–25. https://doi.org/10.1016/j.softx.2015.06.001.

(26) Degiacomi, M. T.; Tian, S.; Greenwell, H. C.; Erastova, V. DynDen: Assessing Convergence of Molecular Dynamics Simulations of Interfaces. *Computer Physics Communications* **2021**, *269*, 108126. https://doi.org/10.1016/j.cpc.2021.108126.

(27) Erastova, V.; Degiacomi, M. T.; G. Fraser, D.; Greenwell, H. C. Mineral Surface Chemistry Control for Origin of Prebiotic Peptides. *Nature Communications* **2017**, *8* (1), 2033. https://doi.org/10.1038/s41467-017-02248-y.

(28) Goldie, S. J.; Bush, S.; Cumming, J. A.; Coleman, K. S. A Statistical Approach to Raman Analysis of Graphene-Related Materials: Implications for Quality Control. *ACS Applied Nano Materials* **2020**, *3* (11), 11229–11239. https://doi.org/10.1021/acsanm.0c02361.

(29) Pollard, A. J.; Paton, K. R.; Clifford, C. A.; Legge, E. *Characterisation of the Structure of Graphene*, Practice Guide 145.; National Physical Laboratory (NPL): London, 2017.

(30) Hernandez, Y.; Nicolosi, V.; Lotya, M.; Blighe, F. M.; Sun, Z.; De, S.; T., McGovernI.; Holland, B.; Byrne, M.; Gun'Ko, Y. K.; Boland, J. J.; Niraj, P.; Duesberg, G.; Krishnamurthy, S.; Goodhue, R.; Hutchison, J.; Scardaci, V.; Ferrari, A. C.; Coleman, J. N. High-Yield Production of Graphene by Liquid-Phase Exfoliation of Graphite. *Nat Nano* **2008**, *3* (9), 563–568. https://doi.org/10.1038/nnano.2008.215.

(31) Farhan, A. M.; Awwad, A. M. Densities, Viscosities, and Excess Molar Enthalpies of 2-Pyrrolidone + Butanol Isomers at T = (293.15, 298.15, and 303.15) K. *Journal of Chemical & Engineering Data* **2009**, *54* (7), 2095–2099. https://doi.org/10.1021/je801006q.

(32) Živković, N. v; Šerbanović, S. S.; Kijevčanin, M. Lj.; Živković, E. M. Volumetric and Viscometric Behavior of Binary Systems 2-Butanol + PEG 200, + PEG 400, + Tetraethylene Glycol Dimethyl Ether, and + N-Methyl-2-Pyrrolidone. *Journal of Chemical & Engineering Data* **2013**, *58* (12), 3332–3341. https://doi.org/10.1021/je400486p.
